# Bioactivity and Metabolome Mining of Deep-Sea Sediment-Derived Microorganisms Reveal New Hybrid PKS-NRPS Macrolactone from *Aspergillus versicolor* PS108-62

**DOI:** 10.3390/md21020095

**Published:** 2023-01-28

**Authors:** Florent Magot, Gwendoline Van Soen, Larissa Buedenbender, Fengjie Li, Thomas Soltwedel, Laura Grauso, Alfonso Mangoni, Martina Blümel, Deniz Tasdemir

**Affiliations:** 1 GEOMAR Centre for Marine Biotechnology (GEOMAR-Biotech), Research Unit Marine Natural Products Chemistry, GEOMAR Helmholtz Centre for Ocean Research Kiel, Am Kiel-Kanal 44, 24106 Kiel, Germany; 2 Centro Interdisciplinar de Química e Bioloxía (CICA), Facultade de Ciencias, Universidade de Coruña, 15071 Coruna, Spain; 3 Section Deep-Sea Ecology and Technology, Helmholtz Center for Polar and Marine Research, Alfred Wegener Institute, Am Handelshafen 12, 27570 Bremerhaven, Germany; 4 Dipartimento di Agraria, Università degli Studi di Napoli Federico II, Via Università 100, 80055 Portici, Italy; 5 Dipartimento di Farmacia, Università degli Studi di Napoli Federico II, Via Domenico Montesano 49, 80131 Napoli, Italy; 6 Faculty of Mathematics and Natural Sciences, Kiel University, Christian-Albrechts-Platz 4, 424118 Kiel, Germany

**Keywords:** Arctic, deep-sea sediment, *Aspergillus*, OSMAC, antimicrobial activity, *Candida albicans*, untargeted metabolomics, molecular networking, GNPS, hybrid PKS-NRPS macrolactone

## Abstract

Despite low temperatures, poor nutrient levels and high pressure, microorganisms thrive in deep-sea environments of polar regions. The adaptability to such extreme environments renders deep-sea microorganisms an encouraging source of novel, bioactive secondary metabolites. In this study, we isolated 77 microorganisms collected by a remotely operated vehicle from the seafloor in the Fram Strait, Arctic Ocean (depth of 2454 m). Thirty-two bacteria and six fungal strains that represented the phylogenetic diversity of the isolates were cultured using an One-Strain-Many-Compounds (OSMAC) approach. The crude EtOAc extracts were tested for antimicrobial and anticancer activities. While antibacterial activity against methicillin-resistant *Staphylococcus aureus* (MRSA) and *Enterococcus faecium* was common for many isolates, only two bacteria displayed anticancer activity, and two fungi inhibited the pathogenic yeast *Candida albicans*. Due to bioactivity against *C. albicans* and rich chemical diversity based on molecular network-based untargeted metabolomics, *Aspergillus versicolor* PS108-62 was selected for an in-depth chemical investigation. A chemical work-up of the SPE-fractions of its dichloromethane subextract led to the isolation of a new PKS-NRPS hybrid macrolactone, versicolide A (**1**), a new quinazoline (−)-isoversicomide A (**3**), as well as three known compounds, burnettramic acid A (**2**), cyclopenol (**4**) and cyclopenin (**5**). Their structures were elucidated by a combination of HRMS, NMR, [α]_D_, FT-IR spectroscopy and computational approaches. Due to the low amounts obtained, only compounds **2** and **4** could be tested for bioactivity, with **2** inhibiting the growth of *C. albicans* (IC_50_ 7.2 µg/mL). These findings highlight, on the one hand, the vast potential of the genus *Aspergillus* to produce novel chemistry, particularly from underexplored ecological niches such as the Arctic deep sea, and on the other, the importance of untargeted metabolomics for selection of marine extracts for downstream chemical investigations.

## 1. Introduction

Marine sediments are formed by sinking planktonic matter, which are continually alimented from the water column and represent the largest source of organic carbon on earth [1,2,3]. The seafloor represents a heterogeneous surface, where microbial cell counts are 10 to 10,000 times higher than in productive ocean-surface waters [4]. The seafloor sediment composition depends on a number of factors including primary production, nutrient status, currents, and sedimentation rate [5], thus providing a diverse array of different substrates for microbial growth. Consequently, the seafloor holds a colossal microbial diversity, which, however, is only partially described [4]. Microorganisms thriving in deep-sea sediments (>1000 m depth) experience high pressure, low temperatures and poor nutrient levels [6]. Those harsh conditions impact gene expression and the metabolism of microorganisms, making them promising sources for the discovery of new secondary metabolites [7,8]. Deep-sea sediment communities are technically more challenging to access and thus have remained mostly understudied compared to microorganisms that inhabit shallow and coastal sediments. Hence, microorganisms from deep-sea sediments hold an underexplored potential for biodiscovery [9,10].

With 4708 compounds described in 2018, marine fungi nowadays represent the third largest source of marine natural products (MNPs) [11]. The MarinLit database [12] counts 7608 compounds alone for the phylum Ascomycota, the dominant fungal phylum in the marine realm, a large portion (approx. 25%) being reported from the genus *Aspergillus*. Although it is one of the most studied fungal genera, this genus remains a very prolific source of new metabolites [9,10,11,13]. Taxonomically, *Aspergillus* is closely related to the genus *Penicillium* (both belonging to family Aspergillaceae in the fungal class Eurotiomycetes)*,* another heavily investigated fungal genus. Based on a recent re-evaluation, the genus *Aspergillus* now encompasses 446 species [14], of which many, e.g., *A. sydowi, A. versicolor, A. niger* and *A. westerdijkiae* originate from the marine realm including the deep sea, mangroves, or marine macro-organisms [15,16,17]. Due to its wide prevalence in nature and the high culturability under laboratory conditions, *Aspergillus* continues to be a dominant genus in the discovery of new natural products from different classes, such as alkaloids, peptides, polyketides, terpenes, sterols and cerebrosides [17].

As deep-sea microorganisms represent a largely untapped reservoir for biodiscovery, we collected sediments (−2454 m) from a station in the long-term ecological research (LTER) observatory HAUSGARTEN, in the Fram Strait [18], in order to establish a deep-sea sediment culture collection. An OSMAC (One-Strain-Many-Compounds) approach [19] was conducted to assess the impact of different culture conditions on the bioactivity and the chemical space of the isolates. The fungus *A. versicolor* PS108-62 revealed interesting antimicrobial activity and contained several unknown molecular clusters in untargeted UPLC-MS/MS metabolomics analysis, and thus, it was prioritized for in-depth chemical analyses. Chromatographic separation of the SPE fractions obtained from the dichloromethane subextract of this fungus led to the isolation and characterization of a new macrolide, versicolide A (**1**), a new quinazoline (−)-isoversicomide A (**3**) and the known metabolites burnettramic acid A (**2**), cyclopenol (**4**) and cyclopenin (**5**). In this study, we describe the diversity, cultivation, bioactivity and chemical profiling of the microbial community isolated from Arctic deep-sea sediment followed by in-depth chemical investigation of the selected strain *A. versicolor* PS108-62. 

## 2. Results

### 2.1. Diversity and Bioactivity of Microorganisms Derived from Arctic Deep-Sea Sediments

**Figure 1:**
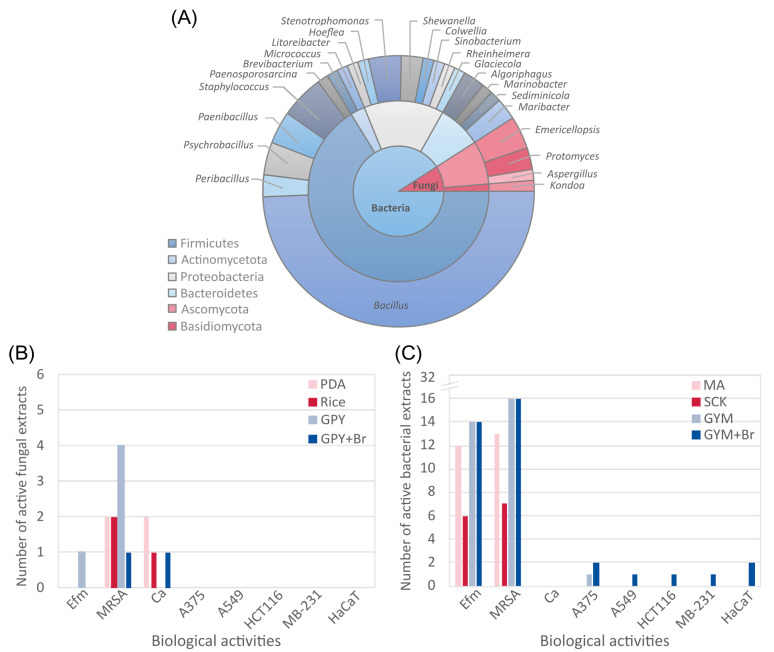
Taxonomic diversity of the isolates (**A**). Bioactive potential (>80% inhibition at 100 µg/mL) of crude extracts from 6 representative deep-sea fungi cultivated in 4 media (**B**) and 32 representative deep-sea bacteria grown in 4 media (**C**). Test organisms: Efm: *Enterococcus faecium*, MRSA: methicillin-resistant *Staphylococcus aureus,* Ca: *Candida albicans*. Cell lines: A-375: human malignant melanoma, HCT-116: human colon cancer, A-549: human lung adenocarcinoma, MDA-MB-231: human breast adenocarcinoma, HaCaT: human non-cancerous keratinocytes. Culture media for fungi: potato dextrose agar (PDA), rice medium (Rice), glucose peptone yeast (GPY) and GPY supplemented with KBr (GPY+Br); culture media for bacteria: marine agar (MA), starch casein KNO_3_ (SCK), glucose yeast malt (GYM) and GYM supplemented with KBr (GYM+Br).

An Arctic deep-sea sediment sampling campaign by a remotely operated vehicle (ROV), followed by inoculation and purification, allowed us to isolate 70 bacterial and 7 fungal strains. Sanger sequencing of the 16S rRNA gene and the ITS1-5.8S rRNA gene-ITS2 region classified the isolates into four bacterial and two fungal phyla, respectively (Figure 1A). Fifty-one strains belonged to the bacterial phylum Firmicutes, of which 38 were identified as *Bacillus* spp.. The other bacterial strains were affiliated to the phyla Proteobacteria (eleven isolates), Bacteroidetes (six isolates), and Actinomycetota (two isolates) (Figure 1A). For the fungi, six isolates were affiliated with the phylum Ascomycota, of which three were of the genus *Emericellopsis*, two *Protomyces* and one *Aspergillus*; the single isolate of the phylum Basidiomycota belonged to the genus *Kondoa* (Figure 1A). The taxonomical classification of all bacteria and fungi are shown in Supplementary Table S1. 

Due to redundancy of several species based on the sequencing results (i.e., identical sequence), especially in the highly abundant bacterial genus *Bacillus*, we selected 32 bacteria and 6 fungi representing the taxonomic diversity for a small-scale cultivation campaign by an OSMAC approach and assessed their biotechnological potential. In order to expand the chemical diversity of the selected isolates, each fungal and bacterial strain was cultured in three different solid media that varied in carbon and nitrogen source(s) and salt concentrations. The fungi were grown on potato dextrose (PDA), rice (Rice), and glucose peptone yeast (GPY) agar, while the bacterial isolates were cultivated on marine agar (MA), starch casein KNO_3_ (SCK) and glucose yeast malt (GYM) agar. Additionally, GPY (bacteria) and GYM (fungi) media were supplemented with potassium bromide, because bromide is a common salt ion in seawater, and many marine metabolites contain bromine substitution [20]. Therefore, we added potassium bromide (KBr) to the culture media to induce bromination of the biosynthesized compounds. As bromine can have toxic effects on microbes [21], the KBr addition was restricted to two media, GYM+Br and GPY+Br. 

Of a total of 152 cultures (32 bacteria and 6 fungi each on 4 media), 136 cultures were successfully cultivated and subsequently extracted with EtOAc, while SCK medium did not support the growth of 14 bacterial isolates, and GPY+Br did not provide an adequate substrate for another two bacterial isolates. The EtOAc crude extracts were tested against (i) a set of infectious human pathogens: the ESKAPE panel that is composed of *Enterococcus faecium,* methicillin-resistant *Staphylococcus aureus* (MRSA*), Klebsiella pneumoniae, Acinetobacter baumannii, Pseudomonas aeruginosa,* and *Escherichia coli*; (ii) two opportunistic fungal pathogens, the yeast *Candida albicans* and the yeast-like *Cryptococcus neoformans*; and (iii) four human cancer cell lines (malignant melanoma cell line A375, the colon cancer cell line HCT116, the lung adenocarcinoma cell line A549, the breast adenocarcinoma line MDA-MB-231) plus the non-cancerous human keratinocyte cell line HaCaT. In total, high activity (defined as growth inhibition of ≥80% at test concentration of 100 µg/mL) was observed for 70 extracts in at least one assay. Activities against Gram-positive bacteria (MRSA and *E. faecium*) were most frequent (Figure 1B,C); yet, none of the extracts inhibited the growth of Gram-negative bacteria. For the fungal isolates, the highest number of bioactive extracts was derived from GPY cultures; four extracts exhibited activity against MRSA and one against *E. faecium* (Figure 1B, Table S2). High activity against the pathogenic yeast *C. albicans* was observed in PDA, Rice and GPY+Br extracts of *Aspergillus versicolor* PS108-62 and of *Emericellopsis maritima* PS108-61 only when cultured on PDA (Figure 1B). As for the bacteria, the cultivation medium had again a clear impact on the bioactivity profile of the extracts (Figure 1C). Cultures grown in SCK medium produced the least number of bioactive extracts with only six showing activity against *E. faecium* and seven against MRSA, while almost double that amount of MA extracts (13 against MRSA and 12 against *E. faecium*) showed activities against the Gram-positive pathogens (Figure 1C). There was no difference in antibacterial activity of GYM and GYM+Br extracts; notably, only the GYM+Br extracts of two bacterial isolates showed high anticancer activities, namely, *Rheinheimera perlucida* PS108-96 (against A-375 cells) and *Bacillus licheniformis* PS108-67a (against all tested cell lines, Figure 1C). Supplementary Tables S2 and S3 outline the in vitro activity of all bacterial and fungal crude extracts.

### 2.2. Strain Selection for Large Scale Chemical Investigation 

In order to select the most suitable strains for detailed chemical investigations, we employed a three-tier selection approach based on (i) unique biological activities, (ii) diverse chemical profiles and (iii) extract yields. For the first tier, activity against Gram-positive human pathogens was common among the deep-sea isolates (Figure 1B,C). Inhibitory activity against *C. albicans* was unique to two fungi, and cancer cell lines were only inhibited by two bacterial isolates. Consequently, the bacterial strain *B. licheniformis* PS108-67a was pre-selected because two of its four crude extracts inhibited the growth of multiple cell lines. More precisely, the GYM+Br medium extract of *B. licheniformis* showed strong inhibition (≥80%) of all human cancer cell lines and the non-cancerous human keratinocyte cell line HaCaT (Table 1). Its GYM extract also showed moderate (46%) to high (94%) activity against all cell lines. *Aspergillus versicolor* PS108-62 was the second pre-selected (fungal) strain, mainly due to its strong inhibitory activity against *C. albicans* observed in its PDA, Rice and GPY+Br extracts, plus the moderate anticancer and anti-MRSA activities exhibited by its PDA extract (Table 1). 

**Figure 2:**
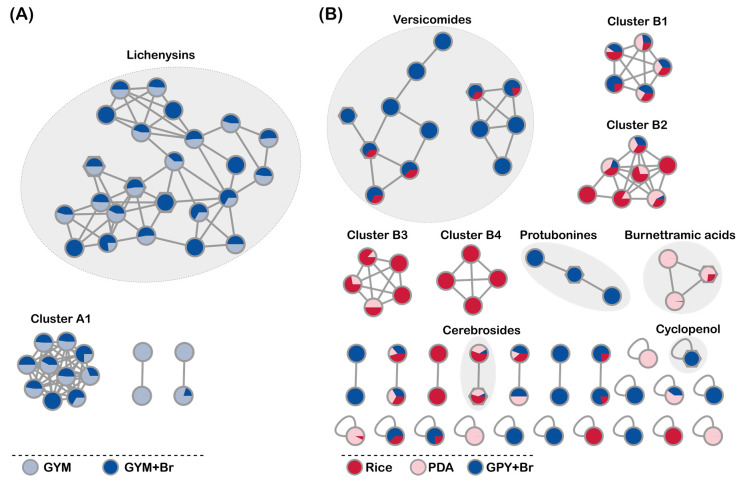
Molecular networks (MNs) displaying the effect of culture media on the chemical space of pre-selected organisms. (**A**) GYM and GYM+Br media extracts of *B. licheniformis* PS108-67a, (**B**) Rice, PDA and GPY+Br extracts of *A. versicolor* PS108-62. Annotated nodes are indicated by hexagonal shapes.

Next, the effects of culture media on the chemical space of the bioactive crude extracts of the two pre-selected strains (*B. licheniformis* PS108-67a and *A. versicolor* PS108-62) were investigated via a UPLC-QToF-MS/MS-based molecular networking (MN) approach [22]. No nodes in the networks could unambiguously be identified via automated annotation through the GNPS platform; however, manual dereplication (Tables S4 and S5) permitted the annotation of several molecular families (highlighted by eclipses) in the MNs shown in Figure 2.

The bacterial isolate *B. licheniformis* PS108-67a displayed a limited diversity of molecular families with only two clusters with more than two nodes in the MN (Figure 2A) to which the GYM and GYM+Br extracts contributed fairly evenly (GYM: 31 nodes; GYM+Br: 36 nodes). Three nodes belonging to the main cluster in the MN could be manually annotated to the known compound class of lichenysins [23] (Figure 2A). The large cluster consisted of nodes of the protonated and doubly charged ion species of the identified compounds, such as lichenysin G8a (C_54_H_97_N_8_O_12_ *m*/*z* 1049.7234 [M + H]^+^/525.3660 [M + 2H]^2+^), lichenysin G13 (C_51_H_91_N_8_O_12_ *m*/*z* 1007.6754 [M + H]^+^/504.3421 [M + 2H]^2+^), lichenysin G14 (C_52_H_93_N_8_O_12_ *m*/*z* 1021.6910 [M + H]^+^/511.3489 [M + 2H]^2+^) and some unknown lichenysin derivatives (Table S4, Figures S1–S5). Furthermore, lichenysin G15 (C_53_H_95_N_8_O_12_ *m*/*z* 1035.7070 [M + H]^+^) was dereplicated from the raw UPLC-MS/MS data; however, the compound did not form a node in the MN. Lichenysins are well-known potent anionic cyclic lipoheptapeptide biosurfactants produced by *B. licheniformis* with reported bioactivities, including cytotoxicity to several cell lines [24], thus, they are most likely also responsible for the observed cytotoxicity including that against the non-cancerous HaCaT cell line. The remaining nodes in this cluster could not be dereplicated and thus could potentially represent new lichenysin analogues. The nodes found in the unannotated cluster A1 had molecular weights above 1000 Da, suggesting that these compounds are also of peptidic or polymeric nature. Due to several facts, i.e., our primary interest in drug-like small molecules (200–600 Da), the previous reports on cytotoxicity of lichenysins [24], and the herein observed general, non-selective toxicity of the *B. licheniformis* PS108-67a crude extract towards non-cancerous cell line HaCaT, this deep-sea strain was not prioritized for further chemical analyses.

The MN for *A. versicolor* PS108-62 indicated a much broader chemical diversity with eight different molecular clusters containing more than two nodes (Figure 2B). The PDA medium extract contributed the lowest number of nodes in the network (26 nodes), while 42 nodes were derived from the Rice extract and 45 from the GPY+Br extract. No nodes in the network could be identified via automated annotation, while manual dereplication associated the known compounds with seven nodes. The chemical structures of the annotated compounds and their MS/MS spectra are shown in Figures S6 and S7–S13, respectively. The single node (C_17_H_15_N_2_O_4_ *m*/*z* 311.1033 [M + H]^+^) from the GPY+Br extract was annotated as cyclopenol, a benzodiazepine alkaloid [25]. The two-node cerebroside cluster, including flavuside B (C_43_H_79_NO_9_Na *m*/*z* 776.5644 [M + Na]^+^) [26,27], was expressed in all media extracts, i.e., Rice, PDA and GPY+Br. One of the three-node clusters was attributed to burnettramic acids that are complex linear PKS-NRPS hybrids. They were predominantly produced in the PDA medium, but they were also observed in the Rice medium extract. Burnettramic acid A (C_41_H_72_NO_12_ *m*/*z* 770.5042 [M + H]^+^) [28,29] was the only annotated compound in this cluster. The biosynthesis of the pyrroloindole alkaloids protubonines appeared to only be induced in the GPY+Br medium. This second small cluster with three nodes was represented by protubonine A (C_19_H_24_N_3_O_4_ *m*/*z* 358.1768 [M + H]^+^) [30,31]. The quinazoline alkaloids produced by the Rice and GPY+Br medium extracts were clustered in two different, larger networks (Figure 2B). Due to the clear differences observed in the experimental MS/MS spectra (Figures S8–10), versicomide A (C_19_H_26_N_3_O_3_ *m*/*z* 344.1975 [M + H]^+^) was clustered in the smaller network (right), while versicomides B (C_19_H_24_N_3_O_3_ *m*/*z* 342.1818 [M + H]^+^) and D (C_19_H_26_N_3_O_4_ *m*/*z* 360.1923 [M + H]^+^) [32] were in the larger network (left). The nodes of the remaining clusters B1–B4 and all other two-node clusters did not return any hits in multiple databases and, thus, may present putatively new compounds. As shown in Figure 2B, the unannotated cluster B4 was unique to the Rice extract, whereas the clusters B1-B3 shared nodes among all media extracts (Rice, PDA and GPY+Br). Notably, the crude extract of *A. versicolor* PS108-62 grown on GPY+Br medium was devoid of any compounds with the characteristic isotopic pattern of a bromine atom(s).

Due to the observed bioactivity and chemical diversity discussed above, we decided to select *A. versicolor* PS108-62 for in-depth chemical studies. The yields of crude *A. versicolor* PS108-62 media extracts (each derived from 10 agar plates) varied significantly (Table 1). When grown on Rice medium, 102.8 mg of extract was recovered; this was four times higher than that obtained from the PDA medium (27.6 mg) and ten times higher than that from the GPY+Br medium extract (10.5 mg). Based on their bioactivity and overall chemical diversity, GYP+Br and Rice extracts both presented good candidates for a large-scale cultivation. However, considering the extraction yields and its contribution to unannotated MN clusters, Rice medium was selected for a large-scale cultivation experiment of *A. versicolor* PS108-62 for subsequent purification and chemical characterization of its metabolites.

### 2.3. Compound Isolation and Structure Elucidation

*Aspergillus versicolor* PS108-62 cultivation was upscaled on 500 plates of rice medium for 21 days, at 22 °C, and subsequently extracted with EtOAc. The crude EtOAc extract was submitted to a modified Kupchan partition scheme [33] to yield three subextracts; aqueous MeOH (Km), DCM (Kd), and *n*-hexane (Kh). Further chemical work focused on the Kd subextract for the following reasons: The limited amount obtained for the Km subextract (34.9 mg) and its high salt content did not permit chemical investigation of this fraction. Additionally, no further work was performed on the Kh (6.355 g) subextract, due to its high lipid content, as indicated by its ^1^H NMR spectrum (Figure S14). The Kd subextract was fractionated on a C18-SPE cartridge to yield 12 fractions, which were tested against MRSA and *C. albicans*. The antimicrobial activity against *C. albicans* and MRSA was tracked to the later eluting fractions (Frs. 8–10). Fr. 8 was the most active SPE fraction with 100% and 93% inhibition at 50 µg/mL against MRSA and *C. albicans,* respectively.

**Figure 3:**
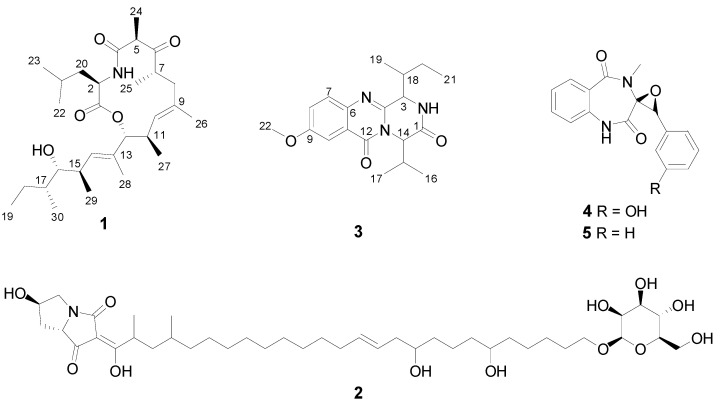
Chemical structures of the compounds **1**–**5** isolated from *A. versicolor* PS108-62. In **3**, alkyl groups at C-3 and C-14 are *trans* to each other.

The new compound **1**, versicolide A (Figure 3), was isolated from the bioactive SPE Fr.9 as a colorless film. The molecular formula C_29_H_49_NO_5_ was deduced from the pseudo-molecular ion at *m*/*z* 514.3510 [M + Na]^+^, requiring 6 degrees of unsaturation. The FT-IR spectrum of **1** contained absorption bands characteristic for amide (*ν*_max_ 3388 cm^−1^), ester (*ν*_max_ 1726 cm^−1^) and keto (*ν*_max_ 1659 cm^-1^) functional groups, which were readily confirmed by the carbonyl resonances observed in the ^13^C NMR spectrum at *δ*_C_ 170.3, 172.1 and 212.8, respectively. Four additional olefinic carbon resonances (*δ*_C_ 134.9, 134.7, 133.2 and 130.6) indicated the presence of two double bounds within **1** (Table 2). Altogether these data account for five degrees of unsaturation; thus, **1** had to be monocyclic. The ^1^H NMR and DEPT-HSQC spectra pinpointed to the presence of 10 methyl groups: one being primary (H_3_-19), seven secondary (H_3_-22, H_3_-23, H_3_-24, H_3_-25, H_3_-27, H_3_-29, H_3_-30) and two olefinic (H_3_-26, H_3_-28). Moreover, the presence of three diastereotopic methylene protons (H_2_-8, H_2_-18 and H_2_-20), two olefinic methine protons (H-10, H-14), two oxymethine protons (H-12, H-16), and seven methine protons were evidenced (Table 2).

**Figure 4:**
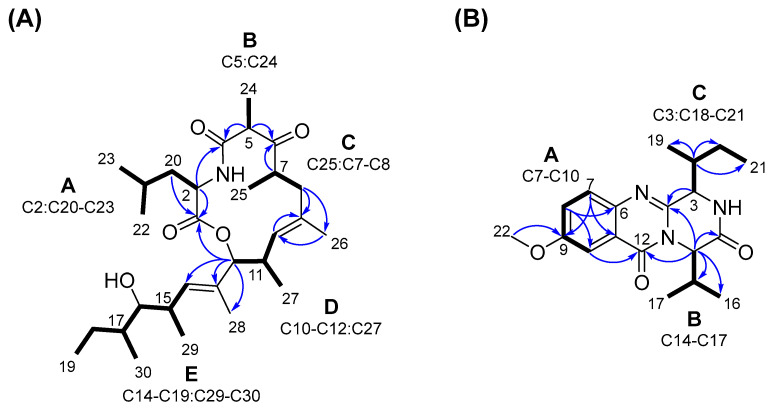
Key COSY (bold) and HMBC (blue arrows) correlations observed (**A**) for compound **1** and **(B)** for compound **3**. The letters **A**–**E** depict COSY spin systems.

Five spin systems (**A**–**E**) were identified in the ^1^H-^1^H COSY spectrum of **1** (Figure 4A). The ^1^H and ^13^C chemical shifts of C-2 (*δ*_H_ 4.44 dd, *J* = 8.4, Hz; *δ*_C_ 51.5) were characteristic of an alpha-carbon of an amino acid. Based on the COSY correlations between H-2/H_2_-20, H_2_-20/H-21, H-21/H_3_-22 and H_3_-21/H_3_-23, the spin system **A** was assigned as leucine, a branch-chained amino acid. The smallest spin system **B** was composed of H-5 (*δ*_H_ 3.73 q, *J* = 7.1 Hz) and H_3_-24 (*δ*_H_ 1.29 d, *J* = 7.1 Hz). The third proton network **C** included H_3_-25 (*δ*_H_ 1.07 d, *J* = 7.0 Hz) that coupled to H-7 (*δ*_H_ 3.24 ddq, *J* = 11.3, 2.2, 7.0 Hz), which in turn coupled with H-8 (*δ*_H_ 1.84 d, *J* = 13.2/*δ*_H_ 2.54 dd, *J* = 13.2, 11.3 Hz). Spin system **D** was formed by the olefinic methine H-10 (*δ*_H_ 4.80 d, *J* = 9.2 Hz) and H-11 (*δ*_H_ 2.69 ddq, *J* = 10.3, 9.2, 6.9 Hz), which further coupled with the oxymethine proton H-12 (*δ*_H_ 4.51 d, *J* = 10.3 Hz) and H_3_-27 (*δ*_H_ 0.76 d, *J* = 6.8 Hz). The last and the largest spin system **E** mainly included the protons of the linear side chain. It consisted of the primary methyl group H_3_-19 (*δ*_H_ 0.87 t, *J* = 7.3 Hz) that coupled to H_2_-18 (*δ*_H_ 1.12/1.42, m). The latter showed a spin coupling with H-17 (*δ*_H_ 1.44, m), which, based on the COSY correlation, was linked to H_3_-30 (*δ*_H_ 0.91 d, *J* = 6.4 Hz) and the oxymethine proton H-16 (*δ*_H_ 3.20 t, *J* = 5.6 Hz). The spin system further continued through H-15 (*δ*_H_ 2.64 m, *J* = 6.8 Hz) that coupled to H-16, H-14 (*δ*_H_ 5.51 d, *J* = 9.8 Hz) and H_3_-29 (*δ*_H_ 0.94 d, *J* = 6.8 Hz) (Figure 4A).

The COSY fragments (**A**–**E**) were linked together by key HMBC cross-peaks allowing the assembly of the 13-membered macrocyclic core structure (Figure 4A). The segments **A** and **B** had to be connected based on the ^1^H-^13^C correlations of both H-2 and H-5 with the amide carbonyl (C-4). Fragments **B** and **C** were easily attached by the long-range couplings between H-5/C-6 and H-7/C-6. The connection of the segments **C** and **D** was based on the correlations between H-8/C10 and H_3_-26/C-10, and vice versa, by H-10/C-8 and H-10/C-26. The macrocyclic ring was fixed by the HMBC correlations of H-2, H_2_-20 (segment **A**) and H-12 (segment **D**) with C-1 carbonyl. Final HMBC correlations from H-12 to C-13, C-14 and C-28; from H_3_-28 to C-12, C-13 and C-14; and from H-14 to C-12, C-15, C-16 and C-28 readily attached the side chain (segment **E**) to macrocyclic core at C12, completing the gross structure of **1**.

**Figure 5:**
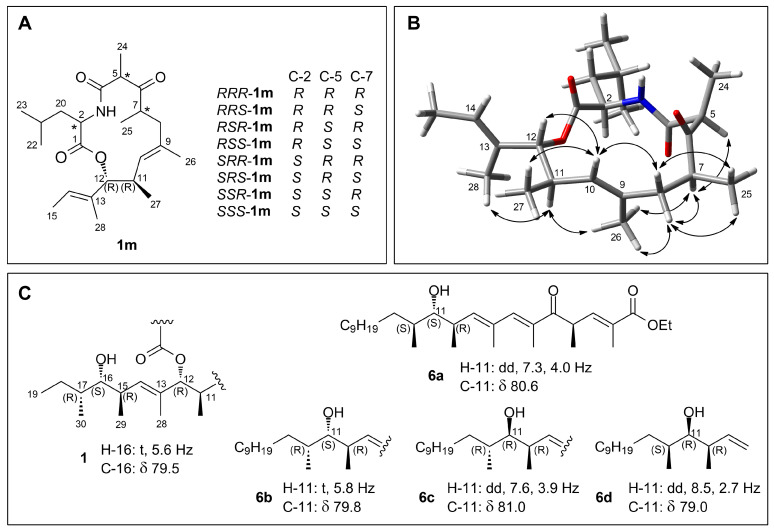
(**A**) The eight diastereomers of the model compound **1m** of the macrocyclic moiety of versicolide A (**1**) used for DFT calculations. Atom numbering is the same as in **1**. (**B**) The NOESY correlations observed for versicolide A (**1**) are fully consistent with the lower-energy conformer of model compound *RSS*-**1m**. (**C**) The synthetic analogues of khafrefungin **6a**–**6d** used to assign the relative configuration of the side chain of **1**, with diagnostic NMR data. For clarity, compounds **6b** and **6c** are represented as the enantiomers of the compounds actually synthesized. Examination of the original ^1^H NMR spectrum of compound **6c** present in the Supporting Information of [35] revealed that wrong values of the coupling constants were reported for **6c** in the main paper; the correct values are reported here.

Configuration of versicolide A (**1**) was determined by DFT-NMR studies and comparison with synthetic model compounds from the literature. Versicolide A (**1**) contains eight stereocenters, giving as many as 128 possible pairs of enantiomers. Therefore, the problem was simplified by first studying the configuration of the 13-membered lactone ring. The simplified model compounds **1m** (Figure 5A) was used for these calculations. Compound **1m** contains five stereocenters. The *RR* or *SS* relative configuration of the contiguous C-11 and C-12 was determined using NMR data, namely, the large coupling constant between H-11 and H-12, showing their *anti* relationship, and the NOESY correlations of H_3_-27 with H_3_-28 and H-14, showing the C-27 methyl group and the C-13/C-19 side chain to be *gauche* oriented (see also Figure 5B). The absolute configuration at this position was assumed to be 11*R*,12*R* on biosynthetic grounds, by analogy with closely related fungal metabolites thermolides [34] that are likely to have a related biosynthesis. Therefore, models of the eight possible diastereomers of **1m** with the 11*R*,12*R* configuration (*RRR*-**1m**, *RRS*-**1m**, *RSR*-**1m**, and so on; see Figure 5A) were the object of DFT studies.

Following our usual protocol [36], a conformational search on these model compounds was performed by molecular dynamics (see Section 4.7 for details), and for each diastereomer, 8 to 19 conformers with population >1% at 298 K were identified (Table S6). These were used for prediction of the ^1^H and ^13^C NMR chemical shifts. The isotropic shielding values, calculated at the mPW1PW91/6-311+G(2d,p)/PCM(MeOH) level, were converted to chemical shift using the linear regression parameters determined by Pierens [37] and available on the CHESHIRE website (http://cheshirenmr.info/). The results of these calculations, summarized in Table S6, univocally identified *RSS*-**1m** as the stereoisomer best-fitting experimental data, because it showed by far the best RMSD for ^1^H and ^13^C chemical shifts (0.091 ppm and 2.09 ppm, respectively) and showed a 99.98% probability in the DP4+ analysis [38] (Table S7). Computational results were fully supported by NOESY data. The eight lowest-energy conformers of *RSS*-**1m**, accounting for 96% of the conformer population, showed a very similar conformation of the macrocycle and only differed in the conformation of the isobutyl and 2-buten-2-yl groups linked at C-2 and C-12, respectively. All the cross-peaks observed in the NOESY spectrum were fully consistent with this conformation (Figure 5B). These results defined the 2*R*,5*S*,7*S*,11*R*,12*R* configuration of the macrocyclic part compound **1**.

The relative configuration of the side chain of versicolide A (**1**) was assigned as (15R*,16S*,17R*) by comparison of NMR data reported for compounds **6a**–**6d**, four diastereomeric synthetic analogues of khafrefungin [35] (Figure 5C): the chemical shift of the carbinol carbon atom C-16 (δ 79.5) of versicolide A (**1**) and the multiplicity of the corresponding proton H-16 (t, *J* = 5.6 Hz) matched very well the data reported for the diastereomer **6b**, having the *RSR* configuration.

The relative configuration of the side chain to the macrocycle was studied by DFT prediction of NMR parameters of the whole molecule. The two possible stereoisomers (2*R*,5*S*,7*S*,11*R*,12*R*,15*R*,16*S*,17*R*, denoted as **1** in the following text, and 2*R*,5*S*,7*S*,11*R*,12*R*,15*S*,16*R*,17*S*, denoted as tris-*epi*-**1** in the following text) were examined. Rather than performing a conformational search of **1** and tris-*epi*-**1** from scratch (which would be long and difficult because the size and flexibility of **1** would result in a huge number of possible conformers), we took advantage of existing conformational information. Therefore, 20 conformers were generated by linking in a combinatorial approach the 5 lowest energy conformers of *RSS*-**1m** (all conformers above 5%, accounting for 88.2% of population) with the four lowest-energy conformers of the *RSR* side chain (or their respective mirror images) generated in an independent calculation (all conformers above 5%, accounting for 90.1% of population). The two sets of 20 conformers were optimized by DFT at the B3LYP/6-31G(d,p)/SMD level, and no remarkable conformational change during optimization was observed for any of them, thus showing that the macrocyclic part of the molecule does not affect much the conformational space of the side chain, and vice versa, and therefore that the combinatorial approach chosen was appropriate. The ^1^H and ^13^C NMR chemical shifts of the two stereoisomers were calculated as described above (Table S8), and the results clearly pointed to compound **1** as the correct stereoisomer, in that it showed better RMSD (^1^H: 0.092 ppm, ^13^C: 1.88 ppm) than tris-*epi*-**1** (^1^H: 0.108 ppm, ^13^C: 2.07 ppm), while the DP4+ probability of **1** was calculated as 99.93% (Table S9). Therefore, configuration of versicolide A (**1**) was fully determined as 2*R*,5*S*,7*S*,11*R*,12*R*,15*R*,16*S*,17*R*.

Burnettramic acid A (**2**) was the main constituent of the highly active SPE Fr.8. Its structure was unambiguously confirmed by comparison of its HRMS, NMR and specific rotation data with those reported in the literature [28,29]. Burnettramic acid A (**2**) has previously been isolated from a terrestrial *Aspergillus* sp., *A. burnettii* [28] and from the co-culture of *A. versicolor* with *A. chevalier* [29]. Due to low amounts, no compounds could be purified from the SPE Fr.10.

The molecular network (Figure 2) revealed further unannotated clusters with undescribed chemistry; therefore, whenever enough amounts were available, other SPE fractions of the Kd subextract were also considered for compound isolation and for confirmation of the initial dereplication efforts. Compound **3** was isolated as a colorless film from SPE Fr.7. Its HRMS data (*m*/*z* 344.1979 [M + H]^+^, C_19_H_26_N_3_O_3_), as well as ^1^H and ^13^C NMR resonances (Table 2) were identical with those of versicomide A, a quinazoline-type metabolite previously reported from a crab-associated *A. versicolor* sampled from a hydrothermal vent [32], resulting in two possibilities, either compound **3** and versicomide A are the same compounds or they are enantiomers. The specific rotation of **3** (${\left[\alpha \right]}_{D}^{20}$ −25, *c* 0.1, MeOH) that is opposite to the reported data (${\left[\alpha \right]}_{D}^{20}$ +50.6, *c* 0.1, MeOH) [32] apparently indicated that **3** is a new compound with an identical planar structure to versicomide A and with opposite stereochemistry. To confirm the structure of compound **3**, we acquired the full set of 1D and 2D NMR experiments. The molecular formula of compound **3** corresponded to nine degrees of unsaturation, while its molecular weight indicates the presence of one (or an odd number of) nitrogen atom(s). The ^13^C NMR spectrum included resonances typical for two amide carbonyl groups (*δ*_C_ 170.6 and *δ*_C_ 162.7) and seven sp^2^ carbons (*δ*_C_ 149.6, 143.0, 130.3, 126.0, 160.3, 107.1 and 121.9). Detailed analysis of the ^1^H-NMR and DEPT-HSQC spectra showed the presence of a typical aromatic ABX system (H-7 *δ* 7.64 d, *J* = 8.9 Hz; H-8 *δ* 7.44 dd, *J* = 8.9, 2.9 Hz, and H-10 *δ* 7.62 d, *J* = 2.9 Hz). Additionally, we observed an aromatic methoxyl group (*δ*_H_ 3.92, s, H_3_-22), four methyl groups, three of which are secondary (H_3_-16 *δ* 0.98 d, *J* = 6.8 Hz; H_3_-17 *δ* 1.12 d, *J* = 6.7 Hz, and H_3_-19 *δ* 1.20 d, *J* = 7.2 Hz) and one primary (H_3_-21 *δ* 0.96 t, *J* = 7.5 Hz), one diastereotopic methylene group (H_2_-20, *δ* 1.49, m and *δ*_H_ 1.36, m), and finally four methine protons, namely, H-3 (*δ* 4.72 d, *J* = 2.2 Hz), H-14 (*δ* 5.17 d, *J* = 8.3 Hz), H-15 (*δ* 2.32, m) and H-18 (*δ* 2.73, m) (Table 2). The COSY spectrum of **3** indicated the whole proton network to be composed of three spin systems (**A**–**C**) as shown in Figure 4B. As discussed above, fragment **A** was easily deduced from COSY spectrum and *J*-value analysis to belong to a 1,3,4-trisubstituted aromatic system, including the protons of H-7, H-8 and H-10. The spin system **B** was assigned to an isopropyl unit associated with a valine unit (H-14 to H_3_-17), while the spin system **C** was attributed to a sec-butyl moiety of an isoleucine (H-3 to H_3_-21). The three spin systems could be connected via HMBC correlations. The methoxy group H_3_-22 was attached at C-9 of the aromatic ring based on its correlation with C-9. The HMBC cross-peak between H-10/C-12 placed the first amide carbonyl at C-12 that correlated with H-14 of the valine moiety. H-14 further correlated with C-4 (*δ*_C_ 149.7) and C-1, the second amide carbonyl (*δ*_C_ 170.6). Final key HMBC correlations between H-3/C-4 concluded the presence of a Val–Ile-2,5-diketopiperazine unit in **3**. So far, this structure accounted for 7 degrees of unsaturation. Based on the molecular weight and the remaining degrees of unsaturation, **3** had to contain a quinazolinone moiety concluding the planar structure of **3** that was identical to versicomide A [32]. However, the hypothesis that compound **3** is the enantiomer of (+)-versicomide A required H-3 and H-14 to be on the same side of the ring. H-3 lacked an NOE cross-peak with H-14, but exhibited a strong NOE with H-15. The NOE between H-3 and H-15 is diagnostic for a *cis* relationship between H-3 and the isopropyl group of the valine unit at C-14, placing H-3 and H-14 on the opposite sides of the ring. This means that compound **3** must be a diastereomer of (+)-versicomide A, in which the two amino acids have opposite configuration at the α carbon.

This prompted us to re-examine the original (+)-versicomide A literature [32]. In this paper, the configuration of the chiral centers relied on chiral phase HPLC analyses of the acid hydrolysate of the compound. Based on the retention time comparison, the two chiral amino acid units were identified as d-Val and d-*allo*-Ile. However, the reported evidence does not seem entirely solid because, as shown in Supplementary Figure S73 of reference [32], the retention times of the individual amino acids are close, and additional peaks that could match different stereoisomers are present in the chromatogram. Curiously, then, the published structure of (+)-versicomide A [32] contains l-Val and l-Ile (see our Figure S30) adding further discrepancy to the configuration of the stereocenters in this compound. Unfortunately, the authors did not report any NOESY data for versicomide A, to allow us to draw a comparison with our data. The opposite (+) specific rotation value to that of versicomide A, and the NOE data indicated that **3** is definitely a new compound, which we called (−)-isoversicomide A. The identical 1D NMR data of **3** and versicomide A in the same solvent (Table S10) are not impossible in principle, even if they are diastereomers, but it is also possible that the relative configuration of (+)-versicomide A also needs to be revised and that they are in fact enantiomers. We were unable to perform additional experiments to identify the configuration of **3,** due to minute amounts of compound isolated, and hence, we left the stereocenters of **3** unassigned, as shown in Figure 3.

The SPE Fr.4 and Fr.5 were also subjected to RP-HPLC separation and yielded the known benzodiazepine alkaloids cyclopenol (**4**) and cyclopenin (**5**), respectively. Interestingly cyclopenin, which is highly related to cylopenol, was not detected in MN, possibly because of poor ionization. The structures of both compounds were confirmed by comparison of their HRMS and NMR data to the literature data [25,39,40,41], and the stereochemistry was confirmed by comparison of the specific rotation values of **4** (${\left[\alpha \right]}_{D}^{20}$ −170.4, *c* 0.06, MeOH) with that reported in the literature ${\left[\alpha \right]}_{D}^{23}$ −220 (*c* 0.03 MeOH), and **5** (${\left[\alpha \right]}_{D}^{20}$ −195, *c* 0.1, MeOH, reported: ${\left[\alpha \right]}_{D}^{26}\;$−208, *c* 2.4, MeOH). These compounds were originally reported from a terrestrial *Penicillium* sp. [41,42], but later isolated from marine *Aspergillus* spp. [32,43].

### 2.4. Bioactivity Results of Purified Compounds

Due to low isolation yields, only compound **2** and **4** could be tested in vitro for their antimicrobial activity against C. albicans and MRSA. Compound **2** was inactive against MRSA but inhibited the growth of C. albicans with an IC_50_ value of 7.2 µg/mL (positive control nystatin had an IC_50_ value of 2.2 µg/mL). This result is coherent with the previously reported activity of **2** towards C. albicans (IC_50_ value 0.5 µg/mL, MIC value 1 µg/mL) in the literature [28,29]. No antimicrobial activity was detected for compound **4** at the test concentration of 100 µg/mL. Considering the initial anti-Candida activity of the crude fungal extract, it is possible that burnettramic acid A (**2**) contributes to the anti-yeast activity of A. versicolor PS108-62.

## 3. Discussion

The rapid development of resistance towards currently available antibiotics and antifungals urges the search for novel drugs in new ecological niches [32]. Due to access difficulties, the Arctic deep sea has remained largely underexplored for biodiscovery efforts in comparison to other environments [44]. Yet, several studies on psychrophiles and psychrotrophs have shown great potential for biodiscovery. A total of 352 new metabolites have been reported from organisms living in cold environments, between 2006 and 2016 [9]. In this study, we set out to (i) cultivate microorganisms from Arctic deep-sea sediments, (ii) assess their bioactivity and metabolomes and (iii) to isolate new, bioactive compounds.

The culturable microbial fraction associated with the sampled Arctic deep-sea sediments consisted of 70 bacterial isolates, of which over 50% belonged to the genus *Bacillus.* Microbial communities associated with deep-sea sediments vary considerably in the available literature. For instance, Jroundi et al. [45] also found *Bacillus* to be the most abundant genus in Mediterranean deep-sea sediments by both culture-dependent and independent techniques, while several others reported high abundance of Proteobacteria in deep-sea sediments [46]. In the present cultivation-based study, Proteobacteria made up less than 15% of the bacterial isolates. Far fewer studies have looked at the culturable diversity of deep-sea fungi. In most cases, the majority of fungal isolates belonged to genera known from terrestrial environments, i.e., being facultative marine fungi. Here, we were able to recover six isolates belonging to the phylum Ascomycota and one isolate was a basidiomycetous yeast. To the best of our knowledge, we provide the first accounts of cultured *Protomyces* and *Kondoa* from deep-sea sediments, while *Emericellopsis* have previously been isolated from deep-sea sediments of the northern Porcupine Bank in the Atlantic Ocean [47]. *Aspergillus* spp. are commonly reported in deep-sea sediments all over the globe [48], where they have been described as facultative marine fungi that were originally washed in from the land, but have adapted to thrive under extreme conditions [49,50]. Laboratory experiments demonstrated that spores of *A. sydowii* can germinate and grow under high hydrostatic pressures that were equivalent to deep-sea pressure of up to 5000 m and temperatures of 5 °C [51].

Our Arctic deep-sea microbial isolates depicted great potential to produce antimicrobial compounds, and were particularly active against Gram-positive human pathogens. Almost 85% of the isolates showed moderate to high anti-MRSA activity, and 71% showed activity against *E. faecium*. Anti-yeast activity was rare, with only two isolates showing high activity against *C. albicans* and five isolates exhibiting moderate activity against *C. neoformans*. Culture media had a considerable effect on the observed bioactivities. The results obtained from the applied OSMAC approach suggest that the nutrient-rich media GYM/GYM+Br as well as GPY/GPY+Br are favoring the production of bioactive metabolites, as crude extracts originating from these media showed the highest antibiotic activities against MRSA and *E. faecium*. Antibiotic production has frequently been reported in marine microbes when glucose was used as the main carbon source (e.g., [52,53,54]). With regard to the addition of bromide, we did not observe the production of additional brominated compounds in the UPLC-MS profiles of the bioactive extracts through the enrichment of the GYM medium with KBr. However, amongst these extracts were the only ones that showed anticancer and cytotoxic activities. Several studies have previously reported an induction of anticancer compounds after media substitution with bromine salt, as reviewed by Romano et al. [55]; for instance, *Aspergillus flavipes* PJ03-11 produced two new anticancer compounds, flavichalasine N and O, in a rice medium substituted with NaBr [56]. Hence, also in our study, bromine supplementation may have triggered the production of cytotoxic compounds in some isolates, i.e., *Bacillus* PS108-67a and *Rheinheimera* PS108-96. Additionally, the selected fungal isolate *A. versicolor* PS108-62 displayed differential bioactivities after addition of bromine. While the GPY extracts were devoid of any biological activities, the GPY+KBr extract showed potent antifungal activity against *C. albicans.* Through molecular networking, we were able to visualize the effect of the culture media on the strain’s chemical space and demonstrated that the biosynthesis of specific molecular families was media-dependent. The initial dereplication enabled annotation of a few of these molecular families, but a large portion of the metabolome remained unidentified. Still, the MN-based untargeted metabolomics approach proved to be a powerful approach complementary to bioactivity data for selection and work-up of microbial extracts for their new, bioactive chemical constituents.

Genes coding for type I and type III polyketide synthases (PKSs), nonribosomal peptide synthetases (NRPSs), and terpene synthases (TPSs) are common in fungi, including deep-sea fungi [57,58], being responsible for biosynthesis of a vast diversity of metabolites [17,59]. From deep-sea *Aspergillus* spp., numerous polyketides, peptides and terpenoids have been reported [59,60,61]. The fusion of different biosynthetic pathways results in enhanced structural diversity, which is often associated with potent bioactive properties [62]. A range of fungal PKS-NRPS hybrids have been reported that are of linear nature (e.g., burnettramic acid A) [28,63], while macrocyclic PKS-NRPS hybrids, resulting from intramolecular Diels–Alder reactions, are less common [62]. The new versicolide A (**1**) is a unique macrocyclic PKS-NRPS hybrid formed by a polyketide chain linked via leucine. Only very few examples of similar hybrid PKS-NRPS macrolactones have been reported. These include thermolides from *Talaromyces thermophilus* [34] from the fungal class Eurotiomycetes that also includes the genus *Aspergillus*; georatusin from *Geomyces auratus* (class: Leotiomycetes) [64]; metacridamides in *Metarhizium acridum* (class: Sordariomycetes) [65]; and tricholides from cyanobacteria [66]. In contrast to classical PKS-NRPS hybrids that are linked via C-C bonds, these mixed biosynthesis macrolactones have an ester bond linkage between the polyketide and the non-ribosomal peptide units [34]. Due to integration of different amino acids and post-translational modifications, these molecular families can be very diverse; for instance, 13 different thermolide analogues have been reported with varying amino acid substitutions [67]. A retrospective MN analysis indicated that versicolide A (**1**) is part of the unannotated cluster B1, which is composed of five nodes. This suggests that there are more versicolide analogues to be described from *A. versicolor* PS108-62. In the future, we will optimize the fermentation process to pursue isolation of further analogues and determine their bioactivities.

In conclusion, the isolated Arctic deep-sea sediment culture collection provides a great resource for biodiscovery of antimicrobial metabolites, in particular against the Gram-positive pathogens, MRSA or *E. faecium*, while activity against the fungal pathogens was less common among the 77 isolates and only observed in two deep-sea fungi. Furthermore, this study demonstrates that the biotechnological potential of the well-studied fungal genus *Aspergillus* is far from being exhausted, and in particular, isolates from extreme environments, such as the Arctic deep sea, are a promising source of new antimicrobial metabolites. The MN-based untargeted metabolomics and initial bioactivity results allowed us to prioritize this prolific fungus for a chemical work-up, resulting in the isolation of the new PKS-NRPS macrolide versicolide A (**1**). During the isolation process, we also isolated and characterized (−)-isoversicomide A (**3**), a new member of the rare versicomide family. Versicomides are a small group of quinazolines reported from one marine and one terrestrial *Aspergillus* sp. [32,68]. The present study may suggest a higher chemical and stereochemical diversity and broader distribution of this small, but intriguing class of quinazolines in the fungal kingdom.

## 4. Materials and Methods

### 4.1. General Procedures

Specific rotations of the compounds were measured on a monochromatic light source in MeOH at 20 °C on a Jasco P-2000 polarimeter (Jasco, Pfungstadt, Germany). The NMR spectra were acquired on a Bruker AV 600 spectrometer (600 and 150 MHz for ^1^H-NMR and ^13^C-NMR, respectively, Bruker®, Billerica, MA, USA) equipped with a Z-gradient triple resonance cryo-probehead (Bruker®, Billerica, MA, USA) or on a Bruker Avance III spectrometer (500 MHz for ^1^H-NMR). The residual solvent signals were used as internal references: δ_H_ 3.31/δ_C_ 49.0 ppm (MeOD), and δ_H_ 7.24/δ_C_ 77.0 ppm (CDCl_3_), 4-Dimethyl-4-silapentane-1-sulfonic acid (DSS) served as the internal standard. UPLC separations were performed on an Acquity UPLC equipped with a HSS T3 column (High-Strength Silica C18, 1.8 µm, 100 × 2.1 mm, Waters, Milford, MA, USA). HRMS/MS were recorded on an Acquity UPLC I-Class System coupled to a Xevo G2-XS QToF Mass Spectrometer (Waters®, Milford, MA, USA) in positive mode at a mass range of *m*/*z* 190–1200 Da. SPE fractionation was performed on C18 Chromabond (Macherey-Nagel GmbH & Co, Düren, Germany). Preparative HPLC was performed on a Luna 5µ C18(2) column (250 × 21.2 mm, Phenomenex, Torrance, CA, USA) attached to a LaPrep system consisting of a P110 pump (VWR International, Allison Park, PA, USA) with a Dynamic Mixing Chamber (Knauer, Germany), P311 UV/VIS and Labocol Vario-2000 Fraction Collector (Labomatic, Switzerland). HPLC separations were performed using a VWR Hitachi Chromaster system (VWR International, Allison Park, PA, USA) consisting of a 5310 column oven, a 5260 autosampler, a 5110 pump, and a 5430 diode array detector connected in parallel with a VWR Evaporative Light Scattering Detector (ELSD 90, VWR International, Allison Park, PA, USA). The HPLC purifications were achieved using a Luna 5μ C18 column (250 × 4.6 mm, Phenomenex, Torrance, CA, USA) or an X select HSS T3 column (High Strength Silica C18, 2.5 µm, 150 × 4.6mm, Waters, Milford, MA, USA). The water used was MilliQ-water produced by in-house Arium® Water Purification Systems (Sartorius, Germany). EtOAc, *n*-hexane, MeOH and MeCN were purchased from VWR International GmbH (Hannover, Germany). ULC/MS grade MeCN and H_2_O were purchased from Biosolve BV (Dieuze, France). NMR solvents (CD_3_OD and CDCl_3_) were from Roth GmbH (Karlsruhe, Germany).

### 4.2. Strain Isolation and Identification

Four sediment samples were collected by a remotely operated vehicle (ROV) at a water depth of 2454 m from the Farm Strait (geographical position: 79° 04.50’ N, 04° 07.75’ E), in the Arctic Ocean in August 2017 during cruise PS108 of the German research vessel POLARSTERN [69]. Sediment samples were inoculated for 21 days, at room temperature, on four solid media, i.e., marine agar (3.74% marine broth, 1.5% agar), tryptic soy agar (tryptic soy broth 0.3%, NaCl 1%, agar 1.5%), potato dextrose agar (0.4% potato infusion, 2% glucose, agar 1.5%, pH 5.6) and modified Wickerham medium (glucose monohydrate 1.0%, peptone from soymeal 0.5%, malt extract 0.3%, yeast extract 0.3%, NaCl 3%, agar 1.5%) and then stored, at 4 °C. Isolation based on phenotypic differences yielded 70 bacterial and seven fungal isolates. Identification of the fungal isolates was based on internal transcribed spacer (ITS1-5.8S-ITS2) fragment sequences and 16S rRNA gene sequences for bacteria, respectively. For DNA extraction, a freeze-and-thaw protocol was used for bacterial isolates, while a mechanical lysis was used in fungi, as previously described in [70].

PCR amplification was performed using primers ITS1F and ITS4R for fungi [71] and primers Eub27f and 1387r for bacteria [72], in a total reaction volume of 25 µL, consisting of 1 µL of template DNA, 1 µL of each primer (concentration: 10 µM), 12.5 µL of Dream Taq Master mix (ThermoFisherScientific, Schwerte, Germany), and 9.5 µL DNA free water (ThermoFisher Scientific, Schwerte, Germany). The following PCR protocol was used for amplification of fungal DNA: initial denaturation for 85 s, at 94 °C, followed by 30 cycles of denaturation of DNA, at 95 °C, for 35 s, primer annealing, at 55 °C, for 55 s, and elongation for 3 min, at 72 °C. A final elongation step for 10 min, at 72 °C, completed the PCR. For bacterial DNA, PCR was conducted as follows: 30 cycles of each 30 s of DNA denaturation, at 92 °C, primer annealing, at 55 °C, and elongation, at 72 °C. A final elongation step for 5 min, at 72 °C, completed the PCR. The correct length of PCR products was checked by gel electrophoresis on a 1% agarose gel run for 20 min at a voltage of 120 V in 1× TBE buffer, taking 100 bp plus DNA ladder (Thermo Scientific™ GeneRuler™, Sunnyvale, CA, USA) as length standard. PCR products were submitted for Sanger sequencing to the company LGC Genomics GmbH (Berlin, Germany), applying the same primers as used for PCR. The obtained sequences were checked for quality and trimmed using ChromasPro V1.33 (Technelysium Pty Ltd., South Brisbane, Australia). The sequences were then compared to the NCBI Genbank (https://www.ncbi.nlm.nih.gov/genbank/, accessed on 11 November 2022) using the Nucleotide BLAST function. Obtained isolate sequences were deposited in GenBank, assigned accession numbers are given in Table S1**.** All isolates were cryopreserved, at −80 °C, using the Microbank TM system (PRO-LAB Diagnostics, Richmond Hill, Canada).

### 4.3. Initial Cultivation and Extraction for Bioactivity Screening

The fungal strains were pre-cultured for seven days on PDA agar, and the bacterial strains were grown for four days on MA agar, both at 22 °C, in the dark. The bacterial pre-cultures were then used to inoculate the main cultures on four different media, namely, MA (3.74% marine broth, 1.2% agar), GYM (0.4% glucose, 0.4% yeast extract, 0.4% malt extract, 0.2% CaCO_3_, 3% sea salt, 1.2% agar pH 7.2), GYM+Br (0.4% glucose, 0.4% yeast extract, 0.4% malt extract, 0.2% CaCO_3_, 1.8% sea salt, 1.2% KBr, 1.2% agar, pH 7.2) and SCK (1% starch, 0.2% KNO_3_, 0.03% casein, 0.002% CaCO_3_, 0.001% FeSO_4_.7H_2_O, 0.015% betaine, 1.2% agar, pH 7.2). The fungal pre-cultures were used to inoculate main cultures on the four media PDA (0.4% potato infusion, 2% glucose, agar 1.2%, pH 5.6), rice medium (6% rice flour, 3% sea salt, 1.2% agar, pH 7.2), GPY (0.1% glucose, 0.05% peptone, 0.01% yeast extract, 3% sea salt, 1.2% agar, pH 7.2) and GPY+Br (0.1% glucose, 0.05% peptone, 0.01% yeast extract, 1.8% sea salt, 1.2% KBr, 1.2% agar, pH 7.2). For extraction, the cultures were cut into small pieces and mixed by an Ultra-Turrax at 19000 rpm, and were finally extracted twice with EtOAc (2 × 300 mL). The extracts were collected in a separatory funnel and washed twice with 200 mL of Milli-Q^®^ water to remove residual salts and water-soluble compounds. The organic phases were collected and evaporated under reduced pressure (200 bar, 150 rpm, at 40 °C). Each dried extract was re-dissolved in MeOH and filtered through a 13 mm syringe filter with a 0.2 µm PTFE membrane (VWR International, Darmstadt, Germany), dried under nitrogen stream and weighted.

### 4.4. Extraction and Compound Isolation

After 21 days of growth, the rice medium cultures (500 plates) of *A. versicolor* PS108-62 were cut into pieces, homogenized by an Ultra-Turrax at 19000 rpm and extracted with EtOAc (2 × 400 mL per 20 plates). The extracts were collected in a separatory funnel and washed twice with 200 mL of Milli-Q^®^ water to remove salts and water-soluble compounds. The organic phases were combined and evaporated under reduced pressure (200 bar, 150 rpm, at 40 °C). The crude extract (6.51 g) was subjected to a modified Kupchan partition scheme [33] to yield three subextracts, namely, *n*-hexane (Kh, 6.355 g), DCM (Kd, 217.9 mg) and aqueous MeOH (Km, 34.9 mg). A portion of Kd subextract (87.9 mg) was fractionated by C18 SPE cartridge (45 mL/5000mg); each fraction of the SPE was eluted with 50 mL of solvent starting from 100% water (Fr.0) to 100% methanol (Fr.10) with 10% increments of methanol in water. The last fraction (Fr.11) was eluted with a MeOH/DCM mixture (1:9). Fr.9 was subjected to RP-HPLC on a Luna 5μ C18 column (250 × 4.6 mm) with a gradient of MeCN in water from 30:70 to 20:80 in 8 min then to 0:100 in 6 minutes at a flow rate of 1 mL/min to yield an impure fraction. This fraction was re-chromatographed on a X select HSS T3 column (High-Strength Silica C18, 2.5 µm, 150 × 4.6mm) to yield the new compound versicolide A (**1**, 0.5 mg, t_R_ 11.3–12.0 min). A second portion of Kd (130.0 mg) was directly chromatographed on a Luna 5μ C18(2) column (250 × 21.2 mm) and eluted with a H_2_O:MeCN gradient (from 95:5 to 0:100 in 25 min). The fraction at the retention time 23–24 min afforded burnettramic acid A (**2**, 5.3 mg) in a pure state. Remaining compounds were purified from SPE fractions using a Luna 5μ C18 column (250 × 4.6 mm, a flow rate of 1 mL/min) using various H_2_O:MeCN gradients. (−)-Isoversicomide A (**3**, 0.4 mg) was obtained from SPE Fr.7 (gradient: 50:50 to 40:60 in 12 min, t_R_ 8.4–9.0 min). The known metabolites cyclopenol (**4**, 2.5 mg) was derived from Fr.4 (gradient: 80:20 to 38:62 in 14 min, t_R_ 9.2–10.0 min), and cyclopenin (**5**, 0.5 mg) from Fr.5 (gradient: 67:33 to 57:43 in 10 min then to 0:100 in 2 min, t_R_ 8.8–10.5 min).

Versicolide A (**1**): Colorless film; ${\left[\alpha \right]}_{D}^{20}$ −12 (*c* 0.1, MeOH); *v*_max_ 3388, 1726 and 1659 cm^−1^; ^1^H NMR (MeOD, 600 MHz) and ^13^C NMR data (MeOD, 150 MHz), Table 2; (+)-HRESIMS *m*/*z* 514.3510 [M + Na]^+^, C_29_H_49_NO_5_Na (calculated for *m*/*z* 514.3508).

(−)-Isoversicomide A (**3**): Colorless film; ${\left[\alpha \right]}_{D}^{20}$ −25 (*c* 0.1, MeOH). ^1^H NMR (MeOD, 600 MHz) and ^13^C NMR data (MeOD, 150 MHz), Table 2; (+)-HRESIMS *m*/*z* 344.1979 [M + H]^+^, C_19_H_26_N_3_O_3_ (calculated for *m*/*z* 344.1974).

### 4.5. UPLC-QToF-MS Analysis

Chromatograms were acquired with a UPLC I-Class System coupled to a Xevo G2-XS QToF Mass Spectrometer (Waters®, Milford, MA, USA) using a modified protocol described previously [73]. Samples were injected at a concentration of 0.1 mg/mL with an injection volume of 1 µL. The separation was performed using a HSS T3 column, at 40 °C, and at a flow rate of 0.6 mL/min. A gradient of H_2_O + 0.1% formic acid (FA) for the mobile phase A, and MeCN + 0.1% FA, for the mobile phase B. The solvent system was as follows: from 99:1 to 0:100 in 11.5 min followed by washing and reconditioning of the column over 3.5 min. The mass range was set from *m*/*z* 190 to 1200, the capillary voltage was 3.0 kV, the cone and the desolvation gas flow were at 50 and 1200 L/h, respectively. The source temperature was 150 °C and the desolvation temperature 550 °C, and the sampling cone and source offset were set at 40 V and 80 V, respectively. Data-dependent acquisition (DDA) mode was used to select the five most intense precursor ions of each MS spectrum (excluding the peaks corresponding to the isotopic contribution of the ^13^C). Those ions were then fragmented using a collision energy (CE) ramp: low CE from 15–25 eV to high CE of 40–60 eV. Solvent (MeOH) and non-inoculated media extracts were injected under the same conditions and used as controls.

### 4.6. Molecular Network

The raw data were converted to mzXML file format using MSConvert (version 3.0.19059, Vanderbilt University, Nashville, TN, USA). For classical molecular networking of the small-scale culture extracts (strain *B. licheniformis* PS108-67a*:* https://gnps.ucsd.edu/ProteoSAFe/status.jsp?task=a81c111412ec4f6482acb3f72b49139b and strain *A. versicolor* PS108-62: https://gnps.ucsd.edu/ProteoSAFe/status.jsp?task=6a874cb754af46bfba3cae3d80aa6de2 the data was filtered by removing all MS/MS fragment ions within +/− 17 Da of the precursor *m*/*z*. MS/MS spectra were window filtered by choosing only the top 6 fragment ions in the +/− 50 Da window throughout the spectrum. The precursor ion mass tolerance was set to 0.5 Da and a MS/MS fragment ion tolerance of 0.5 Da. A network was then created where edges were filtered to have a cosine score above 0.7 and more than 5 matched peaks. Further, edges between two nodes were kept in the network if and only if each of the nodes appeared in each other’s respective top 10 most similar nodes. Finally, the maximum size of a molecular family was set to 100, and the lowest scoring edges were removed from molecular families until the molecular family size was below this threshold. The spectra in the network were then searched against GNPS’ spectral libraries. The library spectra were filtered in the same manner as the input data. All matches kept between network spectra and library spectra were required to have a score above 0.7 and at least 5 matched peaks. Solvent and media blanks in group G6 were filtered before networking. The networks were visualized in Cytoscape^®^ (version 3.5.1, Institute for SystemsBiology, Seattle, WA, USA).

### 4.7. Computational Details

The eight diastereomeric model compounds **1m** (Figure 5A) of the macrocyclic moiety of versicolide A (**1**) were generated using the Builder module in the INSIGHT II/Discover package (BIOVIA, 5005 Wateridge Vista Drive, San Diego, CA 92121, USA). The conformational space of each diastereomer was explored using high-temperature molecular dynamics (MD) [74]. A series of 10 ns MD runs at 600 K in the CFF91 force field were performed for each diastereomer, using the INSIGHT II/Discover package, and the coordinates were saved every 5 ps, and the resulting 2000 structures from each run were minimized in the same force field.

All the unique (non-duplicated) structures from MD within 5 kcal/mol from the lowest-energy conformer of each stereoisomer were used as input structures for density functional theory (DFT) calculations (Table S6). DFT calculations were performed using the program Gaussian 16 (Revision C.01, Gaussian Inc., Wallingford CT, USA). All conformers were optimized by DFT at the B3LYP/6-31G(d,p) level of theory, and the energy of the structures within 3.5 kcal/mol from the lowest-energy conformer were re-evaluated at the same level of theory using the SMD continuous model for the solvent, MeOH. All the conformers populated more than 1% (roughly those within 2.0 kcal/mol from the lowest-energy conformer) were used for prediction of the ^1^H and ^13^C NMR chemical shifts. The Cartesian coordinates of these conformers are available as Supplementary Information in a file called “compounds1m.txt”.

The isotropic shielding values were calculated using the Gauge Invariant Atomic Orbitals (GIAO) method at the mPW1PW91/6-311+G(2d,p) level and the PCM model for the solvent (MeOH). Average isotropic shieldings for each stereoisomer were calculated using Boltzmann statistics (T = 300 K). Average isotropic shieldings were converted to chemical shift using the linear regression parameters available in the literature for this level of theory [37] (Table S7).

The ^1^H and ^13^C NMR chemical shifts of the two alternative 2*R*,5*S*,7*S*,11*R*,12*R*,15*R*,16*S*,17*R* and 2*R*,5*S*,7*S*,11*R*,12*R*,15*S*,16*R*,17*S* stereoisomers of the full molecule of versicolide A (**1**) were predicted using the same DFT methods and level of theory as described above (Tables S8 and S9). The Cartesian coordinates of the conformers used for prediction are available as Supplementary Information in a file called “compounds1.txt”.

### 4.8. Bacterial and Fungal Bioactivity Assays

The crude extracts were tested against methicillin-resistant *Staphylococcus aureus* DSM 18827*, Escherichia coli* DSM 1576, *Pseudomonas aeruginosa* DSM 1128, *Klebsiella pneumoniae* DSM 30104, *Acinetobacter baumannii* DSM 30007, *Enterococcus faecium* DSM 20477, the yeast-like fungus *Cryptococcus neoformans* DSM 6973 and the yeast *Candida albicans* DSM 1386. All bacterial test strains were cultivated in TSB medium (1.2% tryptic soy broth, 0.5% NaCl), except for *E. faecium*, which was cultivated in M92 medium (3% trypticase soy broth, 0.3% yeast extract, pH 7.0–7.2). M186 medium (1% glucose, 0.5% peptone from soybeans, 0.3% yeast extract, 0.3% malt extract) was used for cultivation of *C. albicans* and *C. neoformans*. Overnight cultures of the test organisms were prepared and diluted to an optical density (600 nm) of 0.01–0.03. To prepare the assay, the crude extracts and fractions (20 mg/mL DMSO stock solution) were dissolved in medium and transferred into a 96-well microtiter plate, and 200 µL of the cell suspension cultures was added to each well. The final test concentration was 100 µg/mL. The inoculated microplates were incubated for 5 h, at 37 °C and 200 rpm (*E. faecium* without shaking), and *C. neoformans* for 7 h, at 28 °C and 200 rpm. The detection of inhibitory effects was performed by adding 10 µL of a resazurin solution (0.3 mg/mL in phosphate-buffered saline) to each well and incubating again for 5–60 min before the fluorescence signal (560 nm/590 nm) was read by the microplate reader (Tecan Infinite M200) as described [75]. For *E. faecium*, the pH indicator bromocresol purple was used. For *C. neoformans* the absorbance at 600 nm was measured to detect cell growth inhibition. The resulting values were compared to a positive antibiotic control (chloramphenicol for *S. aureus*, *E. coli*, *K. pneumoniae*, ampicillin for *E. faecium*, polymyxin B for *P. aeruginosa*, doxycycline for *A. baumannii* and amphotericin B for *C. neoformans*) and a negative control (no compound) on the same plate. For determination of IC_50_ values of pure compounds, a dilution series was prepared and tested as described for crude extracts and fractions. The IC_50_ values were calculated using MS Excel (Microsoft Corporation) as the concentration showing 50% inhibition of the viability on the basis of a negative control (no compound) and compared with the positive control (nystatin).

### 4.9. Anticancer and Cytotoxicity Bioactivity Assays

Four human cancer cell lines, namely, malignant melanoma cell line A375 (CLS, Eppelheim, Germany), colon cancer cell line HCT-116 (DSMZ, Braunschweig, Germany), lung carcinoma cell line A549 (CLS, Eppelheim, Germany), breast cancer line MDA-MB-231 (CLS, Eppelheim, Germany) and the non-cancerous human keratinocyte line HaCaT (CLS, Eppelheim, Germany) were used to evaluate the activity of the crude extracts by monitoring the metabolic activity using the CellTiterBlue Cell Viability Assay (Promega, Mannheim, Germany). HaCaT cells were cultivated in RPMI medium, A549 and MDA-MB-231 cells in DMEM: Ham’s F12 medium (1:1) supplemented with 15 mM HEPES and A375 and HCT116 cells in DMEM medium supplemented with 4.5 g/L of d-Glucose and 110 mg/L of sodium pyruvate. All media were supplemented with L-Glutamine, 10% fetal bovine serum, 100 U/mL of penicillin and 100 mg/mL of streptomycin. Cell cultures were maintained, at 37 °C, under a humidified atmosphere and 5% CO_2_. The cell lines were transferred every 3 or 4 days into fresh medium. For the experimental procedure, cells were seeded in 96-well plates at a concentration of 10.000 cells per well in RPMI. After 24 h incubation, the medium was removed, and 100 µL of the test sample, adjusted to final concentrations of 100 µg/mL by diluting in growth medium, was added to the cells. Doxorubicin, as a standard therapeutic anticancer drug, was used as a positive control. Following addition of the crude extracts, plates were incubated for 24 h, at 37 °C. Afterwards, the assay was performed according to the manufacturer’s instructions (Promega, Mannheim, Germany) and fluorescence was measured using the microplate reader Tecan Infinite M200 at excitation 560 nm and emission of 590 nm.

## 5. Conclusions

The microbiota associated with Arctic deep-sea sediments still remain underexplored for their biotechnological potential. Here, we isolated and cultivated bacteria and fungi from the Arctic deep-sea floor (−2454 m) and investigated their bioactivity. The chemical composition of the two bioactive, pre-selected strains were profiled by molecular network based untargeted metabolomics, which led to the prioritization of *A. versicolor* PS108-62 for downstream chemical work. From this strain, we isolated a new hybrid PKS-NRPS macrolactone, versicolide A, a new quinazoline (−)-isoversicomide A, as well as three known compounds. Our investigation confirms, one more time, the vast potential of polar sediment associated microorganisms for marine biodiscovery efforts, especially when combined with modern methodology such as metabolomics and broad-panel bioactivity screening. This study also highlights the fact that the heavily studied fungal genus *Aspergillus* possesses an enormous talent for producing new compounds with intriguing chemical scaffolds and bioactivity. Our future studies will continue unlocking the biomedical potential of polar deep-sea microorganisms.

## Figures and Tables

**Table 1:** Bioactivities (% growth inhibition at 100 µg/mL) of the crude extracts of the two pre-selected microorganisms, *B. licheniformis* PS108-67a and *A. versicolor* PS108-62. Extract yields derive from cultivation on 10 agar plates. MA: marine agar, GYM: glucose yeast malt, GYM+Br: glucose yeast malt supplemented with KBr, SCK: starch casein KNO_3_, PDA: potato dextrose agar, Rice: rice medium, GPY: glucose peptone yeast, GPY+Br: glucose peptone yeast supplemented with KBr. Cell lines: A-375: human malignant melanoma, HCT-116: human colon cancer, A-549: human lung adenocarcinoma, MDA-MB-231: human breast adenocarcinoma, HaCaT: human non-cancerous keratinocytes. Test organisms: Efm: *E. faecium*, MRSA: Methicillin-resistant *S. aureus*, Kp: *K. pneumoniae*, Ab: *A. baumannii*, Psa: *P. aeruginosa*, Ec: *E. coli*, Ca: *C. albicans*, Cn: *C. neoformans*. Positive controls: doxorubicin (anticancer activity) and chloramphenicol (antimicrobial activity).

Strain	Medium	Yield (mg)	Cell Culture					ESKAPE Panel						Fungi	
			A-375	A-549	MDA-MB-231	HCT-116	HaCaT	Efm	MRSA	Kp	Ab	Psa	Ec	Ca	Cn
*B. licheniformis* PS108-67a	MA	3.2	-	-	-	-	-	-	59	-	-	-	-	-	52
	GYM	6.6	94	46	74	70	79	-	-	-	-	-	-	-	33
	GYM+Br	9.0	99	80	99	99	99	-	40	-	-	-	-	-	-
	SCK	6.1	-	-	-	-	-	100	100	-	-	-	-	-	-
*A. versicolor* PS108-62	PDA	27.6	60	20	43	29	45	-	76	-	-	-	-	95	21
	Rice	102.8	-	-	-	-	-	-	53	-	-	-	-	89	-
	GPY	8.0	-	-	-	-	-	-	-	-	-	-	-	26	-
	GPY+Br	10.5	41	35	48	33	38	-	44	-	-	-	-	89	-
Positive control			76	91	84	95	67	95	94	99	98	100	96	100	96

**Table 2:** NMR data of the new compounds **1** and **3** in CD_3_OD (^1^H 600 MHz, ^13^C 150 MHz).

	Versicolide A (1)		(−)-Isoversicomide A (3)		
Position	*δ*_H_, Mult. (*J* in Hz)	*δ*_C_	Position	*δ*_H_, Mult. (*J* in Hz)	*δ*_C_
1	-	172.1, C	1	-	170.6, C
2	dd (8.4, 6.6)	51.5, CH	2	-	-
3	-	-	3	4.72, d (2.2)	59.7, CH
4	-	170.3, C	4	-	149.6, C
5	3.73, q (7.1)	56.9, CH	5	-	-
6	-	212.8, C	6	-	143.0, C
7	3.24, ddq (11.3, 2.2, 7.0)	46.2, CH	7	7.64, d (8.9)	130.3, CH
8a	2.54, dd (13.2, 11.3)	44.7, CH_2_	8	7.44, dd (8.9, 2.9)	126.0, CH
8b	1.84, d (13.2)		9	-	160.3, C
9	-	134.9, C	10	7.62, d (2.9)	107.1, CH
10	4.80, d (9.2)	130.6, CH	11	-	121.9, C
11	2.69, ddq (10.3, 9.2, 6.9)	35.9, CH	12	-	162.7, C
12	4.51, d (10.3)	87.0, CH	13	-	-
13	-	133.2, C	14	5.17, d (8.3)	62.2, CH
14	5.51, d (9.8)	134.7, CH	15	2.32, m	32.6, CH
15	2.64, m (6.8)	36.5, CH	16	0.98, d (6.8)	20.3, CH_3_
16	3.20, t (5.6)	79.5, CH	17	1.12, d (6.7)	19.3, CH_3_
17	1.44, m	38.7, CH	18	2.73, m	37.7, CH
18a	1.42, m	27.3, CH_2_	19	1.20, d (7.2)	15.6, CH_3_
18b	1.12, m		20a	1.49, m,	24.6, CH_2_
19	0.87, t (7.3)	11.8, CH_3_	20b	1.36, m	
20a	1.61, m	40.1, CH_2_	21	0.96, t (7.5)	12.8, CH_3_
20b	1.52, m		22	3.92, s	56.3, CH_3_
21	1.53, m	26.0, CH			
22	0.91, d (6.4)	23.0, CH_3_			
23	0.88, d (6.3)	22.5, CH_3_			
24	1.29, d (7.1)	14.8, CH_3_			
25	1.07, d (7.0)	18.8, CH_3_			
26	1.68, s	16.1, CH_3_			
27	0.76, d (6.8)	17.4, CH_3_			
28	1.61, s	11.8, CH_3_			
29	0.94, d (6.8)	17.9, CH_3_			
30	0.91, d (6.4)	14.5, CH_3_			

## Data Availability

The sequencing data are openly available in GenBank at NCBI (accession numbers: OP807022-OP807028, OP808238-OP808307). The metabolomics data presented in this study are available on request from the corresponding author.

## Supplementary Materials

The following supporting information can be downloaded at: https://www.mdpi.com/article/10.3390/md21020095/s1, Table S1: Taxonomic classification of all bacteria and fungi derived from Arctic deep-sea sediment. Tables S2–S3: In vitro activity of the crude extracts of the selected microorganisms. Tables S4–S5: Dereplication of *B. licheniformis* PS108-67a and *A. versicolor* PS108-62. Figure S1: Structures of dereplicated compounds from *B. licheniformis* PS108-67a. Figures S2–S5: MS/MS spectra of dereplicated lichenysins. Figure S6: Structures of dereplicated compounds from *A. versicolor* PS108-62. Figures S7–S13: MS/MS spectra of dereplicated compounds from *A. versicolor* PS108-62. Figure S14: ^1^H NMR spectrum of the Kh subextract of *A. versicolor* PS108-62. Figures S15–S23: Spectral data of versicolide A (**1**). Figures S24–S29: Spectral data of (−)-isoversicomide A (**3**). The two text files compounds1m.txt and compounds1.txt contain the Cartesian coordinates of all the structures used for DFT-NMR calculations. Refs in supplementary materials are cited in [23,25,26,27,28,29,30,31,32].

## References

[1] Baker D.D., Alvi K. (2004). Small-molecule natural products: New structures, new activities. Curr. Opin. Biotechnol..

[2] Petro C., Starnawski P., Schramm A., Kjeldsen K.U. (2017). Microbial community assembly in marine sediments. Aquat. Microb. Ecol..

[3] Hedges J.I., Keil R.G. (1995). Sedimentary organic matter preservation: An assessment and speculative synthesis. Mar. Chem..

[4] Jørgensen B.B., Boetius A. (2007). Feast and famine—Microbial life in the deep-sea bed. Nat. Rev. Microbiol..

[5] Roman S., Ortiz-Álvarez R., Romano C., Casamayor E.O., Martin D. (2019). Microbial community structure and functionality in the deep sea floor: Evaluating the causes of spatial heterogeneity in a submarine canyon system (NW Mediterranean, Spain). Front. Mar. Sci..

[6] Skropeta D., Wei L. (2014). Recent advances in deep-sea natural products. Nat. Prod. Rep..

[7] Tortorella E., Tedesco P., Palma Esposito F., January G.G., Fani R., Jaspars M., De Pascale D. (2018). Antibiotics from deep-sea microorganisms: Current discoveries and perspectives. Mar. Drugs.

[8] Deming J.W. (1998). Deep ocean environmental biotechnology. Curr. Opin. Biotechnol..

[9] Soldatou S., Baker B.J. (2017). Cold-water marine natural products, 2006 to 2016. Nat. Prod. Rep..

[10] Giordano D. (2020). Bioactive molecules from extreme environments. Mar. Drugs.

[11] Carroll A.R., Copp B.R., Davis R.A., Keyzers R.A., Prinsep M.R. (2020). Marine natural products. Nat. Prod. Rep..

[12] MarinLit: A Database of the Marine Natural Products Literature. http://pubs.rsc.org/marinlit/.

[13] Blunt J.W., Carroll A.R., Copp B.R., Davis R.A., Keyzers R.A., Prinsep M.R. (2022). Marine natural products. Nat. Prod. Rep..

[14] Houbraken J., Kocsubé S., Visagie C.M., Yilmaz N., Wang X.-C., Meijer M., Kraak B., Hubka V., Bensch K., Samson R.A. (2020). Classification of *Aspergillus*, *Penicillium*, *Talaromyces* and related genera (Eurotiales): An overview of families, genera, subgenera, sections, series and species. Stud. Mycol..

[15] Imhoff J.F. (2016). Natural products from marine fungi—Still an underrepresented resource. Mar. Drugs.

[16] Wang Y.-T., Xue Y.-R., Liu C.-H. (2015). A brief review of bioactive metabolites derived from deep-sea fungi. Mar. Drugs.

[17] Lee Y.M., Kim M.J., Li H., Zhang P., Bao B., Lee K.J., Jung J.H. (2013). Marine-derived *Aspergillus* species as a source of bioactive secondary metabolites. Mar. Biotechnol..

[18] Soltwedel T., Bauerfeind E., Bergmann M., Bracher A., Budaeva N., Busch K., Cherkasheva A., Fahl K., Grzelak K., Hasemanna C. (2016). Natural variability or anthropogenically induced variation. Insights from 15 years of multidisciplinary observations at the Arctic marine LTER site HAUSGARTEN. Ecol. Indic..

[19] Bode H.B., Bethe B., Höfs R., Zeeck A. (2002). Big effects from small changes: Possible ways to explore nature’s chemical diversity. ChemBioChem.

[20] Gribble G.W. (2015). Biological activity of recently discovered halogenated marine natural products. Mar. Drugs.

[21] Bajpai P. (2015). The control of microbiological problems. Pulp Pap. Ind..

[22] Wang M., Carver J.J., Phelan V.V., Sanchez L.M., Garg N., Peng Y., Nguyen D.D., Watrous J., Kapono C.A., Luzzatto-Knaan T. (2016). Sharing and community curation of mass spectrometry data with Global Natural Products Social Molecular Networking. Nat. Biotechnol..

[23] Grangemard I., Bonmatin J.-M., Bernillon J., Das B.C., Peypoux F. (1999). Lichenysins G, a novel family of lipopeptide biosurfactants from *Bacillus licheniformis* IM 1307: Production, isolation and structural evaluation by NMR and mass spectrometry. J. Antibiot..

[24] Madslien E.H., Rønning H.T., Lindbäck T., Hassel B., Andersson M.A., Granum P.E. (2013). Lichenysin is produced by most *Bacillus licheniformis* strains. J. Appl. Microbiol..

[25] Mohammed Y.S., Luckner M. (1963). The structure of cyclopenin and cyclopenol, metabolic products from *Penicillium cyclopium* Westling and *Penicillium viridicatum* Westling. Tetrahedron Lett..

[26] Jiang T., Li T., Li J., Fu H.-Z., Pei Y.-H., Lin W.-H. (2004). Cerebroside analogues from marine-derived fungus *Aspergillus flavipes*. J. Asian Nat. Prod. Res..

[27] Yang G., Sandjo L., Yun K., Leutou A.S., Kim G.-D., Choi H.D., Kang J.S., Hong J., Son B.W. (2011). Flavusides A and B, antibacterial cerebrosides from the marine-derived fungus *Aspergillus flavus*. Chem. Pharm. Bull..

[28] Li H., Gilchrist C.L.M., Lacey H.J., Crombie A., Vuong D., Pitt J.I., Lacey E., Chooi Y.-H., Piggott A.M. (2019). Discovery and heterologous biosynthesis of the burnettramic acids: Rare PKS-NRPS-derived bolaamphiphilic pyrrolizidinediones from an Australian fungus, *Aspergillus burnettii*. Org. Lett..

[29] Li J., Chen M., Hao X., Li S., Li F., Yu L., Xiao C., Gan M. (2019). Structural revision and absolute configuration of burnettramic acid A. Org. Lett..

[30] Lee S.U., Asami Y., Lee D., Jang J.-H., Ahn J.S., Oh H. (2011). Protuboxepins A and B and protubonines A and B from the marine-derived fungus *Aspergillus* sp. SF-5044. J. Nat. Prod..

[31] Lorenzo P., Álvarez R., de Lera A.R. (2014). Total synthesis and structural revision of (–)-protubonine a and (–)-protubonine B. European J. Org. Chem..

[32] Pan C., Shi Y., Chen X., Chen C.-T.A., Tao X., Wu B. (2017). New compounds from a hydrothermal vent crab-associated fungus *Aspergillus versicolor* XZ-4. Org. Biomol. Chem..

[33] Kupchan S.M., Britton R.W., Ziegler M.F., Sigel C.W. (1973). Bruceantin, a new potent antileukemic simaroubolide from *Brucea antidysenterica*. J. Org. Chem..

[34] Guo J.-P., Zhu C.-Y., Zhang C.-P., Chu Y.-S., Wang Y.-L., Zhang J.-X., Wu D.-K., Zhang K.-Q., Niu X.-M. (2012). Thermolides, potent nematocidal PKS-NRPS hybrid metabolites from thermophilic fungus *Talaromyces thermophilus*. J. Am. Chem. Soc..

[35] Wakabayashi T., Mori K., Kobayashi S. (2001). Total synthesis and structural elucidation of khafrefungin. J. Am. Chem. Soc..

[36] Grauso L., Li Y., Scarpato S., Shulha O., Rárová L., Strnad M., Teta R., Mangoni A., Zidorn C. (2020). Structure and conformation of zosteraphenols, tetracyclic diarylheptanoids from the seagrass *Zostera marina*: An NMR and DFT Study. Org. Lett..

[37] Pierens G.K. (2014). ^1^H and ^13^C NMR scaling factors for the calculation of chemical shifts in commonly used solvents using density functional theory. J. Comput. Chem..

[38] Grimblat N., Zanardi M.M., Sarotti A.M. (2015). Beyond DP4: An Improved probability for the stereochemical assignment of isomeric compounds using quantum chemical calculations of NMR shifts. J. Org. Chem..

[39] Teixeira T.R., Rangel K.C., Tavares R.S.N., Kawakami C.M., Santos G.S., Maria-Engler S.S., Colepicolo P., Gaspar L.R., Debonsi H.M. (2021). In Vitro Evaluation of the Photoprotective Potential of Quinolinic Alkaloids Isolated from the Antarctic Marine Fungus Penicillium echinulatum for Topical Use. Mar Biotechnol..

[40] Sohn J.H., Lee Y.-R., Lee D.-S., Kim Y.-C., Oh H. (2013). PTP1B inhibitory secondary metabolites from marine-derived fungal strains *Penicillium* spp. and *Eurotium* sp.. J. Microbiol. Biotechnol..

[41] Li J., Wang J., Jiang C.-S., Li G., Guo Y.-W. (2014). (+)-Cyclopenol, a new naturally occurring 7-membered 2, 5-dioxopiperazine alkaloid from the fungus *Penicillium sclerotiorum* endogenous with the Chinese mangrove *Bruguiera gymnorrhiza*. J. Asian Nat. Prod. Res..

[42] Bracken A., Pocker A., Raistrick H. (1954). Studies in the biochemistry of microorganisms. 93. Cyclopenin, a nitrogen-containing metabolic product of *Penicillium cyclopium* Westling. Biochem. J..

[43] Wang L., Li M., Lin Y., Du S., Liu Z., Ju J., Suzuki H., Sawada M., Umezawa K. (2020). Inhibition of cellular inflammatory mediator production and amelioration of learning deficit in flies by deep sea *Aspergillus*-derived cyclopenin. J. Antibiot. (Tokyo).

[44] Poli A., Finore I., Romano I., Gioiello A., Lama L., Nicolaus B. (2017). Microbial diversity in extreme marine habitats and their biomolecules. Microorganisms.

[45] Jroundi F., Martinez-Ruiz F., Merroun M.L., Gonzalez-Muñoz M.T. (2020). Exploring bacterial community composition in Mediterranean deep-sea sediments and their role in heavy metal accumulation. Sci. Total Environ..

[46] Franco N.R., Giraldo M.Á., López-Alvarez D., Gallo-Franco J.J., Dueñas L.F., Puentes V., Castillo A. (2020). Bacterial composition and diversity in deep-sea sediments from the Southern Colombian Caribbean Sea. Diversity.

[47] Marchese P., Garzoli L., Young R., Allcock L., Barry F., Tuohy M., Murphy M. (2021). Fungi populate deep-sea coral gardens as well as marine sediments in the Irish Atlantic Ocean. Environ. Microbiol..

[48] Vargas-Gastélum L., Riquelme M. (2020). The mycobiota of the deep sea: What omics can offer. Life.

[49] Damare S., Raghukumar C., Raghukumar S. (2006). Fungi in deep-sea sediments of the Central Indian Basin. Deep Sea Res. Part I Oceanogr. Res. Pap..

[50] Mouton M., Postma F., Wilsenach J., Botha A. (2012). Diversity and characterization of culturable fungi from marine sediment collected from St. Helena Bay, South Africa. Microb. Ecol..

[51] Raghukumar C., Raghukumar S., Sheelu G., Gupta S.M., Nath B.N., Rao B.R. (2004). Buried in time: Culturable fungi in a deep-sea sediment core from the Chagos Trench, Indian Ocean. Deep Sea Res. Part I Oceanogr. Res. Pap..

[52] Saha M., Ghosh D., Garai D., Jaisankar P., Sarkar K.K., Dutta P.K., Das S., Jha T., Mukherjee J. (2005). Studies on the production and purification of an antimicrobial compound and taxonomy of the producer isolated from the marine environment of the Sundarbans. Appl. Microbiol. Biotechnol..

[53] Darabpour E., Ardakani M.R., Motamedi H., Ronagh M.T., Najafzadeh H. (2012). Purification and optimization of production conditions of a marine-derived antibiotic and ultra-structural study on the effect of this antibiotic against MRSA. Eur. Rev. Med. Pharmacol. Sci..

[54] Sujatha P., Raju K.B., Ramana T. (2005). Studies on a new marine streptomycete BT-408 producing polyketide antibiotic SBR-22 effective against methicillin resistant *Staphylococcus aureus*. Microbiol. Res..

[55] Romano S., Jackson S.A., Patry S., Dobson A.D.W. (2018). Extending the “one strain many compounds”(OSMAC) principle to marine microorganisms. Mar. Drugs.

[56] Si Y., Tang M., Lin S., Chen G., Feng Q., Wang Y., Hua H., Bai J., Wang H., Pei Y. (2018). Cytotoxic cytochalasans from *Aspergillus flavipes* PJ03-11 by OSMAC method. Tetrahedron Lett..

[57] Rédou V., Navarri M., Meslet-Cladière L., Barbier G., Burgaud G. (2015). Species richness and adaptation of marine fungi from deep-subseafloor sediments. Appl. Environ. Microbiol..

[58] Quemener M., Dayras M., Frotté N., Debaets S., Le Meur C., Barbier G., Edgcomb V., Mehiri M., Burgaud G. (2021). Highlighting the biotechnological potential of deep oceanic crust fungi through the prism of their antimicrobial activity. Mar. Drugs.

[59] Zain ul Arifeen M., Ma Y.-N., Xue Y.-R., Liu C.-H. (2019). Deep-sea fungi could be the new arsenal for bioactive molecules. Mar. Drugs.

[60] Yang L.-J., Peng X.-Y., Zhang Y.-H., Liu Z.-Q., Li X., Gu Y.-C., Shao C.-L., Han Z., Wang C.-Y. (2020). Antimicrobial and antioxidant polyketides from a deep-sea-derived fungus *Aspergillus versicolor* SH0105. Mar. Drugs.

[61] Tian Y., Qin X., Lin X., Kaliyaperumal K., Zhou X., Liu J., Ju Z., Tu Z., Liu Y. (2015). Sydoxanthone C and acremolin B produced by deep-sea-derived fungus *Aspergillus* sp. SCSIO Ind09F01. J. Antibiot..

[62] Boettger D., Hertweck C. (2013). Molecular diversity sculpted by fungal PKS–NRPS hybrids. ChemBioChem.

[63] Wenke J., Anke H., Sterner O. (1993). Pseurotin A and 8-O-demethylpseurotin A from *Aspergillus fumigatus* and their inhibitory activities on chitin synthase. Biosci. Biotechnol. Biochem..

[64] Shi Y.-M., Richter C., Challinor V.L., Grun P., Girela del Rio A., Kaiser M., Schuffler A., Piepenbring M., Schwalbe H., Bode H.B. (2018). Georatusin, a specific antiparasitic polyketide–peptide hybrid from the fungus *Geomyces auratus*. Org. Lett..

[65] Krasnoff S.B., Englich U., Miller P.G., Shuler M.L., Glahn R.P., Donzelli B.G.G., Gibson D.M. (2012). Metacridamides A and B, macrocycles from conidia of the entomopathogenic fungus *Metarhizium acridum*. J. Nat. Prod..

[66] Bertin M.J., Roduit A.F., Sun J., Alves G.E., Via C.W., Gonzalez M.A., Zimba P.V., Moeller P.D.R. (2017). Tricholides A and B and unnarmicin D: New hybrid PKS-NRPS macrocycles isolated from an environmental collection of *Trichodesmium thiebautii*. Mar. Drugs.

[67] Zhang J.-M., Wang H.-H., Liu X., Hu C.-H., Zou Y. (2020). Heterologous and engineered biosynthesis of nematocidal polyketide–nonribosomal peptide hybrid macrolactone from extreme thermophilic fungi. J. Am. Chem. Soc..

[68] Wang N.N., Liu C.Y., Wang T., Li Y.L., Xu K., Lou H.X. (2021). Two new quinazoline derivatives from the moss endophytic fungus *Aspergillus* sp. and their anti-inflammatory activity. Nat. Prod. Bioprospect..

[69] Alfred-Wegener-Institut Helmholtz-Zentrum für Polar- und Meeresforschung (2017). Polar research and supply vessel POLARSTERN operated by the Alfred-Wegener-Institute. JLSRF.

[70] Utermann C., Echelmeyer V.A., Blümel M., Tasdemir D. (2020). Culture-dependent microbiome of the *Ciona intestinalis* tunic: Isolation, bioactivity profiling and untargeted metabolomics. Microorganisms.

[71] White T.J., Bruns T., Lee S., Taylor J. (1990). Amplification and direct sequencing of fungal ribosomal RNA genes for phylogenetics. PCR Protocol: A Guide to Methods and Application..

[72] Lane D.J. (1991). 1 16S/23S rRNA sequencing. Nucleic acid Tech. Bact. Syst..

[73] Buedenbender L., Kumar A., Blümel M., Kempken F., Tasdemir D. (2021). Genomics-and metabolomics-based investigation of the deep-sea sediment-derived yeast, *Rhodotorula mucilaginosa* 50-3-19/20B. Mar. Drugs.

[74] Costantino V., Fattorusso E., Mangoni A., Perinu C., Teta R., Panza E., Ianaro A. (2012). Tedarenes A and B: Structural and stereochemical analysis of two new strained cyclic diarylheptanoids from the marine sponge *Tedania ignis*. J. Org. Chem..

[75] Schneemann I., Nagel K., Kajahn I., Labes A., Wiese J., Imhoff J.F. (2010). Comprehensive investigation of marine actinobacteria associated with the sponge *Halichondria panicea*. Appl. Environ. Microbiol..

