# Supplementary material for: Bioactivity and Metabolome Mining of Deep-Sea Sediment-Derived Microorganisms Reveal New Hybrid PKS-NRPS Macrolactone from Aspergillus versicolor PS108-62
Source: Mar Drugs. 2023 Jan 28;21(2):95. doi: 10.3390/md21020095 (PMC9961484; doi:10.3390/md21020095)
Supplement: Supplementary file 1 [file marinedrugs-21-00095-s001.zip › ArcticAspergillus_Supplementary_Revision.pdf]

Supplementary Information

Bioactivity and metabolome mining of deep-sea sediment derived microorganisms reveal new hybrid PKS-NRPS macrolactone from *Aspergillus versicolor* PS108-62

Florent Magot [1], Gwendoline Van Soen [1], Larissa Buedenbender [1,2], Fengjie Li [1], Thomas Soltwedel [3], Laura Grauso [4], Alfonso Mangoni [5], Martina Blümel [1] and Deniz Tasdemir [1,6*]

[1] GEOMAR Centre for Marine Biotechnology (GEOMAR-Biotech), Research Unit Marine Natural Products Chemistry, GEOMAR Helmholtz Centre for Ocean Research Kiel, Am Kiel-Kanal 44, 24106 Kiel, Germany

[2] Current address: CICA - Interdisciplinary Centre for Chemistry and Biology, University of A Coruña, 15071 A Coruña, Spain

[3] Alfred Wegener Institute, Helmholtz Centre for Polar and Marine Research, Am Handelshafen 12, 27570 Bremerhaven, Germany

[4] Dipartimento di Agraria, Università degli Studi di Napoli Federico II, via Università 100, 80055 Portici (NA), Italy

[5] Dipartimento di Farmacia, Università degli Studi di Napoli Federico II, via Domenico Montesano 49, 80131 Napoli, Italy

[6] Faculty of Mathematics and Natural Sciences, Kiel University, Christian-Albrechts-Platz 4, 424118 Kiel, Germany

* Correspondence: dtasdemir@geomar.de; Tel.: +49-431-600-4430

Supplementary Tables

Table S1. Taxonomic classification of 70 bacteria and 7 fungi derived from Arctic deep-sea sediment.
Table S2. In vitro activity of the crude extracts of the six selected fungi grown in four media.
Table S3. In vitro activity of the crude extracts of 32 selected bacteria grown on four media.
Table S4. Manual dereplication of the main peaks from the UPLC-MS/MS chromatogram of the GYM and GYM+Br media extracts of *B. licheniformis* PS108-67a.
Table S5. Dereplication of the main peaks from the UPLC-MS/MS chromatogram of the GPY+Br, PDA and rice media extracts of *A. versicolor* PS108-62.
Table S6. Results of DFT-NMR studies of model compounds **1m**
Table S7. Predicted NMR chemical shift and DP4+ analysis for diastereomeric model compounds **1m**.
Table S8. Results of DFT-NMR studies of compounds **1** and tris-epi-**1**.
Table S9. Predicted NMR chemical shift and DP4+ analysis for compounds **1** and tris-*epi*-**1**.
Table S10. Comparison of the 1D NMR data of **3** and (+)-versicomide A (both in CD_3OD)

Supplementary Figures

Figure S1. Structures of lichenysins annotated from the GYM and GYM+Br media extracts *Bacillus licheniformis* PS108-67a.
Figure S2. MS/MS spectrum of lichenysin G8a ($C_{54}H_{97}N_8O_{12}$ *m/z* 1049.7234 $[M+H]^+$).
Figure S3. MS/MS spectrum of lichenysin G13 ($C_{51}H_{91}N_8O_{12}$ *m/z* 1007.6754 $[M+H]^+$).
Figure S4. MS/MS spectrum of lichenysin G14 ($C_{52}H_{93}N_8O_{12}$ *m/z* 1021.691 $[M+H]^+$).
Figure S5. MS/MS spectrum of lichenysin G15 ($C_{53}H_{95}N_8O_{12}$ *m/z* 1035.707 $[M+H]^+$).
Figure S6. Structures of the compounds annotated from the GPY+Br, PDA and rice media extrac and SPE fractions of the strain *A. versicolor* PS108-62.
Figure S7. MS/MS spectrum of cyclopenol ($C_{17}H_{15}N_2O_4$ *m/z* 311.1033 $[M+H]^+$).
Figure S8. MS/MS spectrum of (iso)versicomide A ($C_{19}H_{26}N_3O_3$ *m/z* 344.1975 $[M+H]^+$).
Figure S9. MS/MS spectrum of versicomide B ($C_{19}H_{24}N_3O_3$ *m/z* 342.1818 $[M+H]^+$).
Figure S10. MS/MS spectrum of versicomide D ($C_{19}H_{26}N_3O_4$ *m/z* 360.1923 $[M+H]^+$).
Figure S11. MS/MS spectrum of burnettramic acid A ($C_{41}H_{72}NO_{12}$ *m/z* 770.5042 $[M+H]^+$).
Figure S12. MS/MS spectrum of cerebroside derivative ($C_{43}H_{79}NO_9$ *m/z* 776.5644 $[M+Na]^+$).
Figure S13. MS/MS spectrum of protubonine A ($C_{19}H_{24}N_3O_4$ m/z 358.1768 $[M+H]^+$).
Figure S14. ^1H NMR spectrum of the Kh subextract of *A. versicolor* PS108-62 ($CDCl_3$, 500 MHz).
Figure S15. HR-ESIMS data of versicolide A (**1**).
Figure S16. MS/MS spectrum of versicolide A (**1**).
Figure S17. FT-IR spectrum of versicolide A (**1**).
Figure S18. ^1H NMR spectrum of versicolide A (**1**) (MeOD, 600 MHz).
Figure S19. ^{13}C NMR spectrum of versicolide A (**1**) (MeOD, 150 MHz).
Figure S20. DEPT-HSQC spectrum of versicolide A (**1**) (MeOD, 600/150 MHz).
Figure S21. ^1H-^1H COSY NMR spectrum of versicolide A (**1**) (MeOD, 600 MHz).
Figure S22. HMBC spectrum of versicolide A (**1**) (MeOD, 600/150 MHz).
Figure S23. NOESY spectrum of versicolide A (**1**) (MeOD, 600MHz).
Figure S24. ^1H NMR spectrum of (-)-isoversicomide A (**3**) (MeOD, 600 MHz).
Figure S25. ^{13}C NMR spectrum of (-)-isoversicomide A (**3**) (MeOD, 150 MHz).
Figure S26. ^1H-^1H COSY NMR spectrum of (-)-isoversicomide A (**3**) (MeOD, 600 MHz).
Figure S27. DEPT-HSQC spectrum of (-)-isoversicomide A (**3**) (MeOD, 600/150 MHz).
Figure S28. HMBC spectrum of (-)-isoversicomide A (**3**) (MeOD, 600/150 MHz).
Figure S29. NOESY spectrum of (-)-isoversicomide A (**3**) (MeOD, 600 MHz).
Figure S30. Detailed analysis of (+)-versicomide A drawn by Pan et al. [10] for its amino acid constituents.

Supplementary References

Table S1. Taxonomic classification of 70 bacteria and 7 fungi derived from Arctic deep-sea sediment. Identification was based on the 16S rRNA gene for bacteria and ITS1-2 region for fungi. Sequences were compared to the NCBI GenBank using nucleotide BLAST (https://www.ncbi.nlm.nih.gov/genbank/ accessed November 2018). Representative strains selected for initial bioactivity screening are highlighted in blue.

Isolate nam	Isolation medium	Amplicon	Length (Bp	Accession number	Closest related species NCBI GenBank (sequence similarity) [accession number]	Taxonomic classification
PS108-08	TSB3	16S	524	OP808238	*Bacillus simplex* (100%) [CP017704.1]<br>*Bacillus simplex* (100%) [NR_114919.1]<br>*Bacillus simplex* (100%) [NR_112726.1]	*Bacillus simplex*
PS108-10	TSB3	16S	308	OP808239	*Bacillus simplex* (99%) [CP017704.1]<br>*Bacillus simplex* (99%) [NR_114919.1]<br>*Bacillus simplex* (99%) [NR_112726.1]	*Bacillus simplex*
PS108-16	TSB3	16S	527	OP808240	*Bacillus simplex (99%) [CP017704.1]*<br>*Bacillus simplex (99%) [NR_114919.1]*<br>*Bacillus simplex (99%) [NR_112726.1]*	*Bacillus simplex*
PS108-18	TSB3	16S	458	OP808241	*Bacillus simplex* (100%) [CP017704.1]<br>*Bacillus simplex* (100%) [NR_114919.1]<br>*Bacillus simplex* (100%) [NR_112726.1]	*Bacillus simplex*
PS108-27	TSB3	16S	632	OP808242	*Bacillus simplex* (100%) [CP017704.1]<br>*Bacillus simplex* (100%) [NR_114919.1]<br>*Bacillus simplex* (100%) [NR_112726.1]	*Bacillus simplex*
PS108-36a	TSB3	16S	662	OP808243	*Bacillus simplex* (99%) [CP017704.1]<br>*Bacillus simplex* (99%) [NR_114919.1]<br>*Bacillus simplex* (99%) [NR_112726.1]	*Bacillus simplex*
PS108-38	TSB3	16S	1087	OP808244	*Bacillus muralis* (100%) [NR_042083.1]<br>*Bacillus simplex* (99%) [CP017704.1]<br>*Bacillus simplex* (99%) [NR_114919.1]	*Bacillus* sp.
PS108-39	TSB3	16S	1094	OP808245	*Bacillus simplex* (99%) [CP017704.1]<br>*Bacillus simplex* (99%) [NR_114919.1]<br>*Bacillus simplex* (99%) [NR_112726.1]	*Bacillus simplex*
PS108-40	TSB3	16S	565	OP808246	*Bacillus simplex* (99%) [CP017704.1]<br>*Bacillus simplex* (99%) [NR_114919.1]<br>*Bacillus simplex* (99%) [NR_112726.1]	*Bacillus simplex*

PS108-46	TSB3	16S	610	OP808247	*Bacillus simplex* (100%) [CP017704.1]<br>*Bacillus simplex* (100%) [NR_114919.1]<br>*Bacillus simplex* (100%) [NR_112726.1]	*Bacillus simplex*
PS108-48a	TSB3	16S	873	OP808248	*Bacillus muralis* (100%) [NR_042083.1]<br>*Bacillus simplex* (99%) [CP017704.1]<br>*Bacillus simplex* (99%) [NR_114919.1]	*Bacillus* sp.
PS108-51	MB	16S	582	OP808249	*Bacillus simplex* (100%) [CP017704.1]<br>*Bacillus simplex* (100%) [NR_114919.1]<br>*Bacillus simplex* (100%) [NR_112726.1]	*Bacillus simplex*
PS108-56	MB	16S	585	OP808250	*Bacillus simplex* (99%) [CP017704.1]<br>*Bacillus simplex* (99%) [NR_114919.1]<br>*Bacillus simplex* (99%) [NR_112726.1]	*Bacillus simplex*
PS108-63	MB	16S	554	OP808251	*Bacillus simplex* (100%) [CP017704.1]<br>*Bacillus simplex* (100%) [NR_114919.1]<br>*Bacillus simplex* (100%) [NR_112726.1]	*Bacillus simplex*
PS108-64b	MB	16S	373	OP808252	*Bacillus simplex* (100%) [CP017704.1]<br>*Bacillus simplex* (100%) [NR_114919.1]<br>*Bacillus simplex* (100%) [NR_112726.1]	*Bacillus simplex*
PS108-69	MB	16S	514	OP808253	*Bacillus simplex* (99%) [CP017704.1]<br>*Bacillus simplex* (99%) [NR_114919.1]<br>*Bacillus simplex* (99%) [NR_112726.1]	*Bacillus simplex*
PS108-83	MB	16S	537	OP808254	*Peribacillus frigoritolerans* (100%) [MW578406.1]<br>*Peribacillus frigoritolerans* (99%) [OL824805.1]<br>*Peribacillus castrilensis* (99%) [OL61per9301.1]	*Peribacillus frigoritolerans*
PS108-87a	MB	16S	463	OP808255	*Bacillus simplex* (100%) [CP017704.1]<br>*Bacillus simplex* (100%) [NR_114919.1]<br>*Bacillus simplex* (100%) [NR_112726.1]	*Bacillus simplex*
PS108-87b	MB	16S	1026	OP808256	*Bacillus simplex* (100%) [CP017704.1]<br>*Brevibacterium frigoritolerans* (100%) [NR_117474.1]<br>*Bacillus simplex* (100%) [NR_114919.1]	*Bacillus simplex*
PS108-25	TSB3	16S	622	OP808257	*Bacillus pumilis* (100%) [LT906438.1]<br>*Bacillus pumilis* (100%) [KX261622.1]<br>*Bacillus pumilis* (100%) [NR_112637.1]	*Bacillus pumilis*

PS108-26	TSB3	16S	666	OP808258	*Bacillus xiamenensis* (100%) [NR_148244.1]<br>*Bacillus xiamenensis* (100%) [JX680066.1]<br>*Bacillus aerius* (100%) [NR_118439.1]	*Bacillus* sp.
PS108-32	TSB3	16S	607	OP808259	*Bacillus pumilis* (100%) [LT906438.1]<br>*Bacillus pumilis* (100%) [KX261622.1]<br>*Bacillus pumilis* (100%) [NR_112637.1]	*Bacillus pumilis*
PS108-42	TSB3	16S	637	OP808260	*Bacillus pumilis* (99%) [LT906438.1]<br>*Bacillus pumilis* (99%) [KX261622.1]<br>*Bacillus pumilis* (99%) [NR_112637.1]	*Bacillus pumilis*
PS108-53	MB	16S	465	OP808261	*Bacillus zhangzhouensis* (100%) [NR_148786.1]<br>*Bacillus zhangzhouensis* (100%) [JX680133.1]<br>*Bacillus pumilus* (99%) [LT906438.1]	*Bacillus* sp.
PS108-57	MB	16S	634	OP808262	*Bacillus xiamenensis* (100%) [NR_148244.1]<br>*Bacillus xiamenensis* (100%) [JX680066.1]<br>*Bacillus aerius* (100%) [NR_118439.1]	*Bacillus* sp.
PS108-33	TSB	16S	419	OP808263	*Bacillus mycoides* (100%) [CP009692.1]<br>*Bacillus mycoides* (100%) [NR_113990.1]<br>*Bacillus mycoides* (100%) [AM747229.1]	*Bacillus mycoides*
PS108-68	MB	16S	636	OP808264	*Bacillus mycoides* (100%) [CP009692.1]<br>*Bacillus mycoides* (100%) [NR_113990.1]<br>*Bacillus mycoides* (100%) [AM747229.1]	*Bacillus mycoides*
PS108-67a	MB	16S	417	OP808265	*Bacillus licheniformis* (100%) [MF461332.1]<br>*Bacillus sonorensis* (100%) [MF446619.1]<br>*Bacillus paralicheniformis* (100%) [KY694465.1]	*Bacillus licheniformis*
PS108-67b	MB	16S	1199	OP808266	*Bacillus licheniformis* (100%) [MF461332.1]<br>*Bacillus licheniformis* (100%) [KY034375.1]<br>*Bacillus paralicheniformis* (100%) [KY694465.1]	*Bacillus licheniformis*
PS108-54	MB	16S	512	OP808267	*Bacillus hwajinpoensis* (100%) [NR_025264.1]<br>*Bacillus hemicentroti* (99%) [NR_109010.1]<br>*Bacillus algicola* (99%) [NR_029077.1]	*Bacillus hwajinpoensis*
PS108-95	MB	16S	472	OP808268	*Bacillus hwajinpoensis* (100%) [NR_025264.1]<br>*Bacillus hemicentroti* (99%) [NR_109010.1]<br>*Bacillus algicola* (99%) [NR_029077.1]	*Bacillus hwajinpoensis*

PS108-70	MB	16S	368	OP808269	*Bacillus hemicentroti* (99%) [NR_109010.1]<br>*Bacillus hwajinpoensis* (99%) [NR_025264.1]	*Bacillus* sp.
PS108-93	MB	16S	512	OP808270	*Bacillus hwajinpoensis* (100%) [NR_025264.1]<br>*Bacillus hemicentroti* (99%) [NR_109010.1]<br>*Bacillus algicola (99%) [NR_029077.1]*	*Bacillus* sp.
PS108-37	TSB3	16S	648	OP808271	*Bacillus muralis* (100%) [NR_042083.1]<br>*Brevibacterium frigoritolerans* (99%) [NR_117474.1]<br>*Brevibacterium frigoritolerans* (99%) [NR_115064.1]	*Bacillus muralis*
PS108-78	MB	16S	601	OP808272	*Bacillus wiedmannii* (99%) [NR_152692.1]<br>*Bacillus thuringiensis* (99%) [CP020754.1]<br>*Bacillus thuringiensis* (99%) [CP021061.1]	*Bacillus* sp.
PS108-80	MB	16S	438	OP808273	*Bacillus pumilus* (100%) [LT906438.1]<br>*Bacillus pumilus* (100%) [KX261622.1]<br>*Bacillus pumilus* (100%) [NR_112637.1]	*Bacillus* sp.
PS108-82	MB	16S	412	OP808274	*Bacillus subtilis* (100%) [CP029465.1]<br>*Bacillus subtilis* (100%) [MH145363.1]<br>*Bacillus nakamurai* (100%) [NR_151897.1]	*Bacillus* sp.
PS108-85	MB	16S	521	OP808275	*Bacillus simplex* (99%) [CP017704.1]<br>*Bacillus simplex* (99%) [NR_114919.1]<br>*Bacillus simplex* (99%) [NR_112726.1]	*Bacillus* sp.
PS108-90	MB	16S	1144	OP808276	*Bacillus thioparans* (100%) [NR_043762.1]<br>*Bacillus foraminis* (99%) [NR_042274.1]<br>*Bacillus mediterraneensis* (99%) [LT161888.2]	*Bacillus* sp.
PS108-94	MB	16S	438	OP808277	*Bacillus aquimaris* (100%) [NR_025241.1]<br>*Bacillus marisflavi* (99%) [NR_118437.1]<br>*Bacillus vietnamensis* (99%) [NR_113995.1]	*Bacillus aquimaris*
PS108-30	TSB3	16S	509	OP808278	*Psychrobacillus soli* (99%) [NR_137244.1] *Psychrobacillu*<br>*soli* (99%) [KJ956929.1] *Psychrobacillus insolitus* (99%)<br>[NR_042709.1]	*Psychrobacillus* sp.
PS108-34	TSB3	16S	1045	OP808279	*Psychrobacillus soli* (99%) [NR_137244.1] *Psychrobacillu*<br>*soli* (99%) [KJ956929.1]<br>*Psychrobacillus psychrodurans* (99%) [KP219721.1]	*Psychrobacillus* sp.

PS108-36b	TSB3	16S	694	OP808280	*Psychrobacillus soli* (99%) [NR_137244.1] *Psychrobacillu*<br>*soli* (99%) [KJ956929.1]<br>*Psychrobacillus psychrotolerans* (99%) [NR_025409.1]	*Psychrobacillus* sp.
PS108-29a	TSB3	16S	1156	OP808281	*Paenibacillus turicensis* (98%) [NR_114626.1]<br>*Paenibacillus turicensis* (98%) [NR_114624.1]<br>*Paenibacillus turicensis* (97%) [NR_114625.1]	*Paenibacillus* sp.
PS108-48b	TSB3	16S	1067	OP808282	*Paenibacillus sinopodophylli* (99%) [NR_153706.1]<br>*Paenibacillus sinopodophylli* (99%) [KX009022.1]<br>*Paenibacillus endophyticus* (97%) [NR_135705.1]	*Paenibacillus* sp.
PS108-48c	TSB3	16S	563	OP808283	*Paenibacillus tundrae* (99%) [NR_044525.1]<br>*Paenibacillus xylanexedens* (99%) [NR_044524.1]<br>*Paenibacillus amylolyticus* (99%) [NR_025882.1]	*Paenibacillus* sp.
PS108-11b	TSB3	16S	1134	OP808284	*Staphylococcus pasteuri* (100%) [MG757632.1]<br>*Staphylococcus pasteuri* (100%) [NR_114435.1]<br>*Staphylococcus pasteuri* (100%) [NR_024669.1]	*Staphylococcus pasteuri*
PS108-11a	TSB3	16S	438	OP808285	*Staphylococcus pasteuri* (100%) [MG757632.1]<br>*Staphylococcus pasteuri* (100%) [NR_114435.1]<br>*Staphylococcus pasteuri* (100%) [NR_024669.1]	*Staphylococcus pasteuri*
PS108-24	TSB3	16S	476	OP808286	*Staphylococcus saprophyticus* (99%) [MG988385.1]<br>*Staphylococcus saprophyticus* (99%) [NR_074999.2]<br>*Staphylococcus edaphicus* (99%) [NR_156818.1]	*Staphyloccocus saprophyticus*
PS108-58	MB	16S	444	OP808287	*Staphylococcus warneri* (99%) [NR_025922.1]<br>*Staphylococcus pasteuri* (99%) [MG757632.1]<br>*Staphylococcus pasteuri* (99%) [NR_114435.1]	*Staphylococcus warneri*
PS108-17a	TSB3	16S	1088	OP808288	*Paenisporosarcina indica* (99%) [NR_108473.1]<br>*Sporosarcina antarctica* (99%) [NR_044122.1]<br>*Paenisporosarcina quisquiliarum* (99%) [NR_043720.1]	*Paenisporosarcina* sp.
PS108-13	TSB3	16S	494	OP808289	*Peribacillus simplex* (99%) [CP017704.1]<br>*Peribacillus frigoritolerans*(99%) [MW578406.1]<br>*Peribacillus frigoritolerans* (99%) [OL824805.1]	*Peribacillus* sp.
PS108-74	MB	16S	494	OP808290	*Micrococcus yunnanensis* (100%) [NR_116578.1]<br>*Micrococcus luteus* (99%) [LS483396.1]	*Micrococcus yunnanensis*

PS108-66	MB	16S	570	OP808291	*Litoreibacter ascidiaceicola* (99%) [NR_134068.1]<br>*Litoreibacter halocynthiae* (99%) [NR_118472.1]<br>*Litoreibacter albidus* (99%) [NR_112984.1]	*Litoreibacter* sp.
PS108-86	MB	16S	1107	OP808292	*Hoeflea alexandrii* (99%) [NR_042321.1]<br>*Hoeflea halophila* (99%) [NR_108835.1]<br>*Hoeflea marina* (98%) [NR_043007.1]	*Hoeflea alexandrii*
PS108-59	MB	16S	1146	OP808293	*Stenotrophomonas rhizophila* (100%) [NR_121739.1]<br>*Stenotrophomonas rhizophila* (100%) [CP007597.1]	*Stenotrophomonas rhizophila*
PS108-84a	MB	16S	1084	OP808294	*Stenotrophomonas rhizaphila* (100%) [NR_121739.1]<br>*Stenotrophomonas rhizaphila* (100%) [CP007597.1]	*Stenotrophomonas rhizophila*
PS108-84b	MB	16S	1158	OP808295	*Stenotrophomonas rhizaphila* (100%) [NR_121739.1]<br>*Stenotrophomonas rhizaphila* (100%) [CP007597.1]	*Stenotrophomonas rhizophila*
PS108-72	MB	16S	362	OP808296	*Shewanella morhuae* (98%) [NR_113968.1]<br>*Shewanella xiamenensis* (98%) [NR_116732.1]<br>*Shewanella morhuae* (98%) [NR_041299.1]	*Shewanella* sp.
PS108-91	MB	16S	571	OP808297	*Shewanella arctica* (99%) [NR_117528.1]<br>*Shewanella vesiculosa* (99%) [NR_042710.1]<br>*Shenawella livingstonensis* (99%) [NR_025443.1]	*Shewanella* sp.
PS108-60	MB	16S	413	OP808298	*Colwellia sediminilitoris* (99%) [NR_151990.1]<br>*Colwellia sediminilitoris* (99%) [KU696539.1]<br>*Colwellia aestuarii* (99%) [NR_043509.1]	*Colwellia* sp.
PS108-73	MB	16S	340	OP808299	*Sinobacterium norvegicum* (98%) [NR_144601.1]<br>*Sinobacterium norvegicum* (98%) [HG004165.1]<br>*Sinobacterium caligoides* (98%) [NR_109307.1]	*Sinobacterium norvegicum*
PS108-96	MB	16S	380	OP808300	*Arsukibacterium ikkense* (98%) [DQ112664.2]<br>*Alishewanella tabrizica* (98%) [NR_117388.1]<br>*Rheinheimera perlucida* (98%) [NR_042487.1]	*Rheinheimera perlucida*
PS108-98	MB	16S	394	OP808301	*Glaciecola polaris* (99%) [NR_025427.1]<br>*Glaciecola agarilytica* (98%) [NR_043956.1]<br>*Glaciecola chathamensis* (98%) [NR_041397.1]	*Glaciecola polaris*
PS108-77	MB	16S	485	OP808302	*Algoriphagus ratkowskyi* (99%) [NR_042219.1]<br>*Algoriphagus aquimarinus* (99%) [NR_025602.1]<br>*Algoriphagus antarcticus* (99%) [NR_025604.1]	*Algoriphagus ratkowskyi*

PS108-79	MB	16S	572	OP808303	*Algoriphagus ratkowskyi* (99%) [NR_042219.1]<br>*Algoriphagus aquimatinus* (99%) [NR_025602.1]<br>*Algoriphagus antarcticus* (99%) [NR_025604.1]	*Algoriphagus ratkowskyi*
PS108-65	MB	16S	1102	OP808304	*Marinobacter psychrophilus* (100%) [CP011494.1]<br>*Marinobacter psychrophilus* (99%) [NR_043513.1]<br>*Marinobacter antarcticus* (99%) [NR_108299.1]	*Marinobacter psychrophilus*
PS108-76	MB	16S	505	OP808305	*Sediminicola arcticus* (99%) [KM576847.1]<br>*Sediminicola luteus* (99%) [NR_113965.1]<br>*Sediminicola luteus* (99%) [NR_041301.1]	*Sediminicola arcticus*
PS108-75	MB	16S	539	OP808306	*Maribacter arcticus* (99%) [NR_043176.1]<br>*Maribacter orientalis* (99%) [NR_025749.1]	*Maribacter arcticus*
PS108-89	MB	16S	352	OP808307	*Maribacter arcticus* (100%) [NR_043176.1]<br>*Maribacter orientalis* (100%) [NR_025749.1]<br>*Maribacter arcticus* (99%) [NR_044515.1]	*Maribacter arcticus*
PS108-04	WSP30	ITS	547	OP807022	*Emericellopsis maritima* (99%) [NR_144919.1]<br>*Emericellopsis maritima* (99%) [AY632670.1]<br>*Emericellopsis stolkiae* (98%) [NR_156197.1]	*Emericellopsis maritima*
PS108-55	MB	ITS	442	OP807023	*Emericellopsis maritima* (96.5%) [MH871998.1]	*Emericellopsis* sp.
PS108-61	MB	ITS	354	OP807025	*Emericellopsis maritima* (100%) [NR_144919.1]<br>*Emericellopsis minima* (99%) [AY632669.1]<br>*Emericellopsis minima* (99%) [KC987173.1]	*Emericellopsis maritima*
PS108-05	WSP30	ITS	432	OP807026	*Protomyces inouyei* (97%) [NR_121206.1]<br>*Protomyces inouyei* (97%) [DQ497617.1]	*Protomyces* sp.
PS108-06	WSP30	ITS	507	OP807028	*Protomyces inouyei* (97%) [NR_121206.1]<br>*Protomyces inouyei* (97%) [DQ497617.1]	*Protomyces* sp.
PS108-62	MB	ITS	327	OP807024	*Aspergillus versicolor* (99%) [KU729039.1]<br>*Aspergillus versicolor* (99%) [NR_131277.1]<br>*Aspergillus protuberus* (99%) [NR_135353.1]	*Aspergillus versicolor*
PS108-03	PDA	ITS	314	OP807027	*Kondoa aeria* (99%) [KY103882.1]<br>*Kondoa aeria* (99%) [NR_073307.1]<br>*Kondoa malvinella* (96%) [DQ667165.1]	*Kondoa aeria*

Table S2. In vitro activity of the crude extracts of the six selected fungi grown in four media (% inhibition, tested at 100 μg/mL). The symbol (-) indicates inhibition level below 20%. A375: Malignant melanoma, A549: Lung carcinoma, MB231: Breast cancer, HCT116: Colon cancer, HaCaT: human keratinocyte cell line; Efm: *Enterococcus faecium*, MRSA: Methicillin-resistant *Staphylococcus aureus*, Ab: *Acinetobacter baumannii*, Kp: *Klebsiella pneumoniae*, Psa: *Pseudomonas aeruginosa*, Ec: *Escherichia coli;* Ca: *Candida albicans*, Cn: *Cryptococcus neoformans*.

Samples		Cell culture					ESKAPE						Yeasts	
Strains	Medium	A375	A549	MB-231	HCT116	HaCaT	Efm	MRSA	Kp	Ab	Psa	Ec	Ca	Cn
PS108-3	PDA	-	-	-	-	-	-	94	-	-	-	-	-	-
	Rice	-	-	-	-	-	-	100	-	-	-	-	-	-
	GPY	-	-	-	-	-	88	99	-	-	-	-	-	-
	GPY+KBr	-	-	-	-	-	-	52	-	-	-	-	-	26
PS108-4	PDA	-	-	-	-	-	-	82	-	-	-	-	-	-
	Rice	-	-	-	-	-	-	61	-	-	-	-	-	22
	GPY	-	-	-	-	-	-	79	-	-	-	-	-	-
	GPY+KBr	-	-	-	-	-	-	69	-	-	-	-	-	44
PS108-5	PDA	-	-	-	-	-	-	68	-	-	-	-	-	-
	Rice	-	-	-	-	-	51	99	-	-	-	-	-	-
	GPY	-	-	-	-	-	-	89	-	-	-	-	-	-
	GPY+KBr	-	-	-	-	-	-	90	-	-	-	-	-	-
PS108-55	PDA	-	-	-	-	-	-	78	-	-	-	-	-	-
	Rice	-	-	-	-	-	-	67	-	-	-	-	-	26
	GPY	-	-	-	-	-	-	88	-	-	-	-	-	-
	GPY+KBr	-	-	-	-	-	-	75	-	-	-	-	-	45
PS108-61	PDA	25	-	-	-	-	-	66	-	-	-	-	85	20
	Rice	-	-	-	-	-	-	72	-	-	-	-	-	38
	GPY	-	-	-	-	-	-	92	-	-	-	-	-	22
	GPY+KBr	-	-	-	-	-	-	75	-	-	-	-	-	46
PS108-62	PDA	60	20	43	29	45	-	76	-	-	-	-	95	21
	Rice	-	-	-	-	-	-	53	-	-	-	-	89	-
	GPY	-	-	-	-	-	-	-	-	-	-	-	26	-
	GPY+KBr	41	35	48	33	38	-	44	-	-	-	-	89	-
Positive control		76	67	76	66	85	83	98	99	99	99	97	96	96

Table S3. In vitro activity of the crude extracts of 32 selected bacteria grown on four media (%-inhibition, tested at 100 µg/mL). MA: marine agar, SCK: starch casein KNO_3, GYM: glucose yeast malt, GYM+Br: glucose yeast malt supplemented with KBr). The symbol (-) indicates inhibition level below 20%. A375: Malignant melanoma, A549: Lung carcinoma, MB231: Breast cancer, HCT116: Colon cancer, HaCaT: human keratinocyte cell line; Efm: *Enterococcus faecium*, MRSA: Methicillin-resistant *Staphylococcus aureus*, Ab: *Acinetobacter baumannii*, Kp: *Klebsiella pneumoniae,* Psa: *Pseudomonas aeruginosa*, Ec: *Escherichia coli;* Ca: *Candida albicans*, Cn: *Cryptococcus neoformans*.

Samples		Cell culture					ESKAPE						Fungi	
Strains	Media	A375	A549	MB231	HCT116	HaCaT	Efm	MRSA	Kp	Ab	Psa	Ec	Ca	Cn
PS108-13	MA	-	-	-	-	-	100	100	-	-	-	-	-	-
	GYM	-	-	-	-	-	80	53	-	-	-	-	-	-
	GYM+Br	-	-	-	-	-	100	100	-	-	-	-	-	-
	SCK	-	-	-	-	-	-	99	-	-	-	-	-	-
PS108-16	MA	-	-	-	-	-	100	100	-	-	-	-	-	-
	GYM	-	-	-	-	-	100	100	-	-	-	-	-	-
	GYM+Br	-	-	-	-	-	-	100	-	-	-	-	-	-
	SCK	-	-	-	-	-	100	100	-	-	-	-	-	-
PS108-25	MA	-	-	-	-	-	-	48	-	-	-	-	-	-
	GYM	-	-	-	-	-	-	100	-	-	-	-	-	-
	GYM+Br	-	-	-	-	-	-	100	-	-	-	-	-	-
	SCK	-	-	-	-	-	-	45	-	-	-	-	-	-
PS108-36b	MA	-	-	-	-	-	-	-	-	-	-	-	-	-
	GYM	-	-	-	24	-	-	-	-	-	-	-	-	-
	GYM+Br	-	-	-	-	-	-	-	-	-	-	-	-	-
	SCK	-	-	-	-	-	97	95	-	-	-	-	-	-
PS108-37	MA	-	-	-	-	-	100	97	-	-	-	-	-	-
	GYM	-	-	-	27	-	100	100	-	-	-	-	-	-
	GYM+Br	-	-	-	25	-	100	101	-	-	-	-	-	-
	SCK	-	-	-	-	-	88	83	-	-	-	-	-	-
PS108-48b	MA	-	-	-	-	-	-	-	-	-	-	-	-	-
	GYM	-	-	-	-	-	-	31	-	-	-	-	-	-
	GYM+Br	-	-	-	-	-	-	43	-	-	-	-	-	-
	SCK	-	-	-	-	-	-	-	-	-	-	-	-	-
PS108-48c	MA	-	-	-	-	-	-	-	-	-	-	-	-	-
	GYM	-	-	-	-	-	62	49	-	-	-	-	-	-
	GYM+Br	-	-	-	-	-	-	-	-	-	-	-	-	-
	SCK	-	-	-	-	-	-	-	-	-	-	-	-	-
PS108-33	MA	-	-	-	-	-	42	-	-	-	-	-	-	-
	GYM	-	-	-	20	-	100	100	-	-	-	-	-	-
	GYM+Br	-	-	-	22	-	100	99	-	-	-	-	-	-
	SCK	-	-	-	23	-	64	64	-	-	-	-	-	-

PS108-34	MA	-	-	-	-	-	57	26	-	-	-	-	-	-
	GYM	-	-	-	-	35	45	68	-	-	-	-	-	-
	GYM+Br	-	-	-	38	22	-	38	-	-	-	-	-	-
	SCK	-	-	-	25	-	46	71	-	-	-	-	-	-
PS108-39	MA	-	-	-	-	24	100	85	-	-	-	-	-	-
	GYM	-	-	-	20	26	100	100	-	-	-	-	-	-
	GYM+Br	-	-	-	21	-	100	98	-	-	-	-	-	-
	SCK	-	-	-	-	-	100	100	-	-	-	-	-	-
PS108-58	MA	-	-	-	-	-	-	-	-	24	-	-	-	-
	GYM	-	-	-	-	-	100	97	-	-	-	-	-	-
	GYM+Br	-	-	-	-	-	99	97	-	-	-	-	-	-
	SCK	-	-	-	-	-	91	71	-	-	-	-	-	-
PS108-70	MA	-	-	-	-	-	22	32	-	-	-	-	-	-
	GYM	23	-	-	-	-	53	66	-	-	-	-	-	-
	GYM+Br	25	-	-	-	-	44	68	-	-	-	-	-	-
	SCK	-	-	-	22	-	53	-	-	-	-	-	-	-
PS108-74	MA	-	-	-	-	-	34	-	-	-	-	-	-	-
	GYM	-	-	-	-	23	100	99	-	-	-	-	-	-
	GYM+Br	-	-	-	22	28	87	47	-	-	-	-	-	-
	SCK	-	-	-	23	-	77	26	-	-	-	-	-	-
PS108-80	MA	-	-	-	-	-	-	64	-	-	-	-	-	-
	GYM	-	-	-	-	29	27	74	-	-	-	-	-	-
	GYM+Br	-	-	-	20	36	52	75	-	-	-	-	-	-
	SCK	-	-	-	24	-	55	81	-	-	-	-	-	-
PS108-82	MA	-	-	-	-	-	60	56	-	-	-	-	-	-
	GYM	-	-	-	-	-	71	48	-	-	-	-	-	-
	GYM+Br	-	-	-	-	-	79	67	-	-	-	-	-	-
	SCK	-	-	-	-	-	61	45	-	-	-	-	-	-
PS108-85	MA	-	-	-	-	-	100	92	-	-	-	-	-	-
	GYM	20	-	-	-	-	100	99	-	-	-	-	-	-
	GYM+Br	-	-	-	-	-	91	82	-	-	-	-	-	-
	SCK	-	-	-	-	-	53	62	-	-	-	-	-	-
PS108-17	MA	-	-	-	-	-	-	25	-	-	-	-	-	-
	GYM	21	-	-	-	-	-	31	-	-	-	-	-	-
	GYM+Br	36	31	-	-	35	-	43	-	-	-	-	-	23
PS108-54	MA	-	-	39	28	-	100	100	-	-	-	-	-	-
	GYM	-	-	-	-	-	-	54	-	-	-	-	-	-
	GYM+Br	-	-	-	-	-	100	98	-	-	-	-	-	-
PS108-67a	MA	-	-	-	-	-	-	59	-	-	-	-	-	52
	GYM	94	46	-	-	79	-	-	-	-	-	-	-	33
	GYM+Br	99	80	74	70	99	-	40	-	-	-	-	-	-
	SCK	-	-	99	99	-	-	35	-	-	-	-	-	-

PS108-84a	MA	-	-	-	-	-	100	100	-	-	-	-	-	32
	GYM	-	-	-	-	-	100	100	-	-	-	-	-	53
	GYM+Br	-	-	-	-	-	100	100	-	-	-	-	-	-
	SCK	-	-	-	-	-	100	100	-	-	-	-	-	-
PS108-89	MA	-	-	-	-	-	-	-	-	-	-	-	-	29
	GYM	-	-	-	-	-	-	29	-	-	-	-	-	-
	GYM+Br	-	-	-	-	-	-	-	-	-	-	-	-	-
PS108-90	MA	-	-	-	-	-	-	-	-	-	-	-	-	-
	GYM	-	-	-	-	-	-	57	-	-	-	-	-	-
	GYM+Br	-	-	-	-	-	-	51	-	-	-	-	-	-
PS108-94	MA	-	-	-	-	-	27	82	-	-	-	-	-	-
	GYM	24	-	-	-	-	100	100	-	-	-	-	-	28
	GYM+Br	34	-	-	-	-	100	100	-	-	-	-	-	58
PS108-98	MA	-	-	25	-	-	100	100	-	-	-	-	-	-
	GYM	-	-	-	-	-	100	100	-	-	-	-	-	27
	GYM+Br	-	-	-	-	-	100	100	-	-	-	-	-	57
PS108-60	MA	-	-	-	-	-	100	100	-	-	-	-	-	21
	GYM	-	-	-	-	-	-	31	-	-	-	-	-	-
	GYM+Br	-	-	-	-	-	97	93	-	-	-	-	-	21
PS108-65	MA	-	-	-	-	-	95	97	-	-	-	-	-	-
	GYM	-	-	-	-	-	100	97	-	-	-	-	-	-
PS108-66	MA	-	-	-	-	-	33	24	-	-	-	-	-	-
	GYM	-	-	-	-	-	59	95	-	-	-	-	-	-
	GYM+Br	-	-	-	-	-	29	20	-	-	-	-	-	-
PS108-76	MA	-	-	-	-	20	-	-	-	-	-	-	-	-
	GYM	-	-	-	-	-	-	-	-	-	-	-	-	-
PS108-77	MA	-	-	-	-	-	74	46	-	-	-	-	-	-
	GYM	-	-	-	-	-	23	-	-	-	-	-	-	-
	GYM+Br	-	-	-	-	-	70	76	-	-	-	-	-	-
PS108-86	MA	-	-	-	-	-	31	48	-	-	-	-	-	-
	GYM	-	-	-	-	-	78	99	-	-	-	-	-	30
	GYM+Br	-	-	-	-	-	79	99	-	-	-	-	24	-
PS108-91	MA	-	-	-	-	-	100	97	-	-	-	-	-	43
	GYM	-	-	-	-	-	100	99	-	-	-	-	-	-
	GYM+Br	-	-	-	-	-	98	94	-	-	-	-	-	33
PS108-96	MA	-	-	-	-	-	100	100	-	-	-	-	-	-
	GYM	-	-	-	-	-	100	100	-	-	-	-	-	-
	GYM+Br	95	-	-	-	98	100	100	-	-	-	-	-	54
Positive control		89	88	89	88	88	87	95	90	99	99	92	91	100

Table S4. Manual dereplication of the main peaks from the UPLC-MS/MS chromatogram of the GYM and GYM+Br media extracts of *B. licheniformis* PS108-67a.

t_R (min)	Ion formula	Putative compound	Observed *m/z*	Δ ppm	Ref.
10.55	$C_{51}H_{91}N_8O_{12}$	Lichenysin G13	1007.6754	-0.2	[1]
10.74	$C_{55}H_{89}N_8O_{12}$	unknown lichenysin	1053.6635	3.3	
10.96 / 11.03	$C_{52}H_{93}N_8O_{12}$	Lichenysin G14	1021.691	-0.3	[1]
11.31 / 11.43	$C_{53}H_{95}N_8O_{12}$	Lichenysin G15*	1035.707	0.1	[1]
11.69	$C_{54}H_{97}N_8O_{12}$	Lichenysin G8a	1049.7234	1.1	[1]
12.10	$C_{55}H_{93}N_8O_{12}$	unknown lichenysin	1063.7383	-0.3	

*Only dereplicated manually from raw data but not annotated in MN (Figure 2 in the main text)

Table S5. Dereplication of the main peaks from the UPLC-MS/MS chromatogram of the GPY+Br, PDA and rice media extracts of *A. versicolor* PS108-62.

t_R (min)	Ion formula	Compound	Observed *m/z*	Δ ppm	Ref.
2.89	$C_{13}H_{14}N_3$		212.1192	1.9	
3.63	$C_{17}H_{15}N_2O_4$	Cyclopenol	311.1033	1.9	[2]
6.10*	$C_{19}H_{24}N_3O_4$	Protubonine A*	358.1768	0.3	[3,4]
6.42	$C_{19}H_{26}N_3O_4$	Versicomide D	360.1923	-0.3	[5]
7.17	$C_{19}H_{26}N_3O_3$	Versicomide A	344.1975	1.5	[5]
7.26	$C_{19}H_{24}N_3O_3$	Versicomide B	342.1818	0.1	[5]
7.89	$C_{41}H_{72}NO_{12}$	Burnettramic acid A	770.5042	-1.7	[6,7]
7.96 / 8.06 / 8.20	$C_{29}H_{48}NO_6$		506.3488	1.2	
8.77	$C_{19}H_{32}N_{10}ONa$		439.2671	3.0	
10.55	$C_{29}H_{49}NO_5Na$	Versicolide A (new)	514.3511	0.6	
10.76	$C_{22}H_{42}O_6Na$		425.2880	-0.5	
11.53	$C_{18}H_{38}NO$		284.296	0.6	
12.43	$C_{20}H_{42}NO$		312.3268	-0.6	
13.04	$C_{28}H_{44}N$		394.3472	-0.5	
13.70	$C_{43}H_{79}NO_9Na$	Cerebroside deriv.	776.5644	-1.2	[8,9]

*Not observed in Rice and PDA media.

Table S6. Results of DFT-NMR studies of model compounds **1m.**

	RRR-**1n**	*RRS*-**1n**	*RSR*-**1n**	*RSS*-**1n**	*SRR*-**1n**	*SRS*-**1n**	*SSR*-**1n**	*SSS*-**1n**
Unique conformers within 5 kcal/mol force field CFF91	104	53	152	42	103	76	168	58
Unique conformers within 3.5 kcal/mol DFT, optimization B3LYP/6-31G(d,p)	13	19	21	15	51	19	45	20
Conformers populated >1% at 298 K DFT, single point B3LYP/6-31G(d,p)/SMD	9	12	10	11	18	8	19	13
RMSD vs. **1** of predicted ^{13}C NMR chemical shifts mPW1PW91/6-311+G(2d,p)/PCM(MeOH)	2.52	2.61	2.58	2.09	2.86	3.58	2.52	3.15
RMSD vs. **1** of predicted ^{1}H NMR chemical shifts mPW1PW91/6-311+G(2d,p)/PCM(MeOH)	0.198	0.190	0.131	0.091	0.176	0.236	0.169	0.147
DP4+ probability scaled shifts	0.00%	0.00%	0.01%	99.98%	0.00%	0.00%	0.01%	0.00%

Table S7. Predicted NMR chemical shift and DP4+ analysis for diastereomeric model compounds **1m.**

Functional	Solvent?	Basis Set?	Type of Data
mPW1PW91	PCM	6-311+G(d,p)	Scaled Shifts

			s DP4+	0.00%	0.00%	0.01%	99.98%	0.00%	0.00%	0.01%	0.00%
			DP4+	-	-	-	-	-	-	-	-
	Nuclei	sp2?	Experimental	RRR-1m	RRS-1m	RSR-1m	RSS-1m	SRR-1m	SRS-1m	SSR-1m	SSS-1m
C-1	C	x	172.1	175.1	173.3	172.8	173.9	174.0	174.3	173.0	172.9
C-2	C		51.5	52.4	52.8	51.5	51.9	54.7	55.7	53.9	56.8
C-4	C	x	170.3	168.2	168.7	166.7	167.4	170.8	170.9	170.2	170.3
C-5	C		56.9	58.1	54.0	57.4	59.9	56.9	51.4	57.6	57.0
C-6	C	x	212.8	212.8	221.2	219.6	214.2	219.8	222.0	219.9	222.5
C-7	C		46.2	50.9	47.8	42.9	46.3	50.5	51.3	47.8	50.7
C-8	C		44.7	44.5	46.6	47.4	45.3	43.2	46.0	43.6	44.8
C-9	C	x	134.9	141.73	137.21	138.17	140.59	138.12	136.71	140.21	138.35
C-10	C	x	130.6	132.46	131.97	132.00	131.02	130.50	131.28	130.11	132.67
C-11	C		35.9	38.32	36.82	37.53	37.44	37.13	36.61	37.08	36.78
C-12	C		87.0	84.68	84.28	83.18	84.12	83.84	84.35	83.64	84.55
C-20	C		40.1	41.52	38.45	39.91	40.43	39.45	39.27	40.93	40.36
C-21	C		26.0	28.02	27.54	27.53	27.94	27.85	27.97	27.70	27.83
C-22	C		23.0	20.60	20.30	20.77	20.93	20.47	20.34	20.84	20.78
C-23	C		22.5	20.96	21.38	21.08	20.89	20.78	20.84	20.34	20.36
C-24	C		14.8	12.73	17.33	11.85	12.48	19.83	21.39	13.96	17.87
C-25	C		18.8	20.11	17.53	19.42	18.50	16.52	15.97	19.39	18.55
C-26	C		16.1	17.30	15.12	15.38	15.57	18.33	15.11	18.17	15.50
C-27	C		17.4	16.89	16.74	16.80	16.77	17.04	16.62	17.11	16.60
H-2	H		4.44	4.63	3.89	4.49	4.56	4.06	3.79	4.42	4.02
H-5	H		3.73	3.70	3.51	3.37	3.84	3.69	3.69	3.20	3.59
H-7	H		3.24	2.75	3.04	3.31	3.48	3.13	2.85	3.11	3.21
H-8a	H		2.54	2.51	2.40	2.42	2.57	2.54	2.33	2.42	2.59
H-8b	H		1.84	1.96	2.21	2.07	1.92	2.39	2.30	2.10	1.94
H-10	H		4.80	4.98	4.96	4.90	4.86	4.80	4.94	4.71	5.04
H-11	H		2.69	2.72	2.76	2.74	2.77	2.72	2.79	2.79	2.86
H-12	H		4.51	4.97	4.46	4.68	4.60	4.39	4.30	4.70	4.40
H-20a	H		1.61	1.49	1.60	1.50	1.51	1.57	1.51	1.53	1.82
H-20b	H		1.52	1.44	1.58	1.47	1.40	1.50	1.49	1.48	1.59
H-21	H		1.53	1.59	1.65	1.57	1.51	1.59	1.57	1.60	1.50
H3-22	H		0.91	0.91	0.86	0.86	0.87	0.95	0.96	0.95	0.91
H3-23	H		0.88	0.92	0.89	0.88	0.90	0.89	0.89	0.93	0.91
H3-24	H		1.29	1.17	1.38	1.43	1.20	1.36	1.35	1.44	1.32
H3-25	H		1.07	1.06	1.15	1.09	1.06	1.22	1.21	1.10	1.14
H3-26	H		1.68	1.37	1.65	1.61	1.70	1.68	1.70	1.60	1.78
H3-27	H		0.76	0.76	0.71	0.71	0.75	0.78	0.72	0.81	0.75

Table S8. Results of DFT-NMR studies of compounds **1** and tris-*epi*-**1**.

**Compound**	**1**	tris-*epi*-**1**
Conformers populated >1% at 298 K<br>DFT, optimization B3LYP/6-31G(d,p)/SMD	16	15
RMSD vs. experimental chemical shifts of **1** of predicted ^{13}C NMR chemical shifts<br>mPW1PW91/6-311+G(2d,p)/PCM(MeOH)	1.88	2.07
RMSD vs. experimental chemical shifts of **1** of predicted ^{1}H NMR chemical shifts<br>mPW1PW91/6-311+G(2d,p)/PCM(MeOH)	0.092	0.108
DP4+ probability<br>scaled shifts	99.93%	0.07%

Table S9. Predicted NMR chemical shift and DP4+ analysis for compounds **1** and tris-*epi*-**1**.

Functional	Solvent?	Basis Set?	Type of Data
mPW1PW91	PCM	6-311+G(d,p)	Scaled Shifts

	Nuclei	sp2?	Experimental	Isomer 1	Isomer 2	Isomer 3	Isomer 4	Isomer 5
			sDP4+	99.93%	0.07%	-	-	-
			DP4+	-	-	-	-	-
C-1	C	x	172.1	173.7	173.7			
C-2	C		51.5	51.1	50.8			
C-4	C	x	170.3	167.3	167.3			
C-5	C		56.9	59.3	59.4			
C-6	C	x	212.8	215.9	215.8			
C-7	C		46.2	46.9	46.8			
C-8	C		44.7	45.0	45.0			
C-9	C	x	134.9	140.04	139.94			
C-10	C	x	130.6	131.77	132.11			
C-11	C		35.9	36.83	37.34			
C-12	C		87.0	87.16	87.10			
C-13	C	x	133.2	135.37	135.62			
C-14	C	x	134.7	138.79	139.26			
C-15	C		36.5	38.38	38.98			
C-16	C		79.5	78.85	78.64			
C-17	C		38.7	39.33	38.60			
C-18	C		27.3	27.77	28.49			
C-19	C		11.8	11.25	11.70			
C-20	C		40.1	39.66	39.30			
C-21	C		26.0	27.36	27.28			
C-22	C		23.0	21.31	20.80			
C-23	C		22.5	21.10	21.52			
C-24	C		14.8	12.74	12.54			
C-25	C		18.8	18.58	18.51			
C-26	C		16.1	15.87	15.87			
C-27	C		17.4	16.74	16.69			
C-28	C		11.8	11.94	12.19			
C-29	C		17.9	17.03	16.47			
C-30	C		14.5	11.74	10.54			
H-2	H		4.44	4.58	4.63			
H-5	H		3.73	3.87	3.85			
H-7	H		3.24	3.43	3.43			
H-8a	H		2.54	2.62	2.61			
H-8b	H		1.84	1.91	1.90			
H-10	H		4.80	4.89	4.89			
H-11	H		2.69	2.82	2.79			
H-12	H		4.51	4.40	4.32			
H-14	H		5.51	5.52	5.49			
H-15	H		2.64	2.54	2.50			
H-16	H		3.20	3.01	3.03			
H-17	H		1.44	1.46	1.54			
H-18a	H		1.42	1.40	1.45			
H-18b	H		1.12	1.24	1.34			
H3-19	H		0.87	0.90	0.93			
H-20a	H		1.61	1.51	1.48			
H-20b	H		1.52	1.40	1.44			
H-21	H		1.53	1.55	1.54			
H3-22	H		0.91	0.87	0.89			
H3-23	H		0.88	0.89	0.92			
H3-24	H		1.29	1.19	1.19			
H3-25	H		1.07	1.08	1.08			
H3-26	H		1.68	1.71	1.72			
H3-27	H		0.76	0.79	0.83			
H3-28	H		1.61	1.67	1.66			
H3-29	H		0.94	0.94	0.90			
H3-30	H		0.91	0.88	0.83			

Table S10. Comparison of the 1D NMR data of **3** and (+)-versicomide A (both in CD_3OD)

**Position**	**Versicomide A*** **δ_H, Mult. (*J* in Hz)**	**3** **δ_H, Mult. (*J* in Hz)**	**Versicomide A*** **δ_C**	**3** **δ_C**
1	-	-	170.5, C	170.6, C
2	-	-	-	-
3	4.72, d (2.3)	4.72, d (2.2)	59.8, CH	59.7, CH
4	-	-	149.7, C	149.6, C
5	-	-	-	-
6	-	-	143.1, C	143.0, C
7	7.65, d (8.9)	7.64, d (8.9)	130.3, CH	130.3, CH
8	7.45, dd (8.9, 2.9)	7.44, dd (8.9, 2.9)	125.9, CH	126.0, CH
9	-	-	160.3, C	160.3, C
10	7.63, d (2.9)	7.62, d (2.9)	107.5, CH	107.1, CH
11	-	-	122.1, C	121.9, C
12	-	-	162.7, C	162.7, C
13	-	-	-	-
14	5.18, d (8.2)	5.17, d (8.3)	62.3, CH	62.2, CH
15	2.33, m	2.32, m	32.2, CH	32.6, CH
16	0.99, d (6.8)	0.98, d (6.8)	20.5, CH_3	20.3, CH_3
17	1.13, d (6.7)	1.12, d (6.7)	19.5, CH_3	19.3, CH_3
18	2.74, m	2.73, m	37.9, CH	37.7, CH
19	1.21, d (7.2)	1.20, d (7.2)	15.8, CH_3	15.6, CH_3
20	1.49, m, 1.36 m	1.49, m, 1.36, m	24.7, CH_2	24.6, CH_2
21	0.96, t (7.4)	0.96, t (7.5)	12.9, CH_3	12.8, CH_3
22	3.94, s	3.92, s	56.4, CH_3	56.3, CH_3

* Data reported for versicomide A in the literature [10]

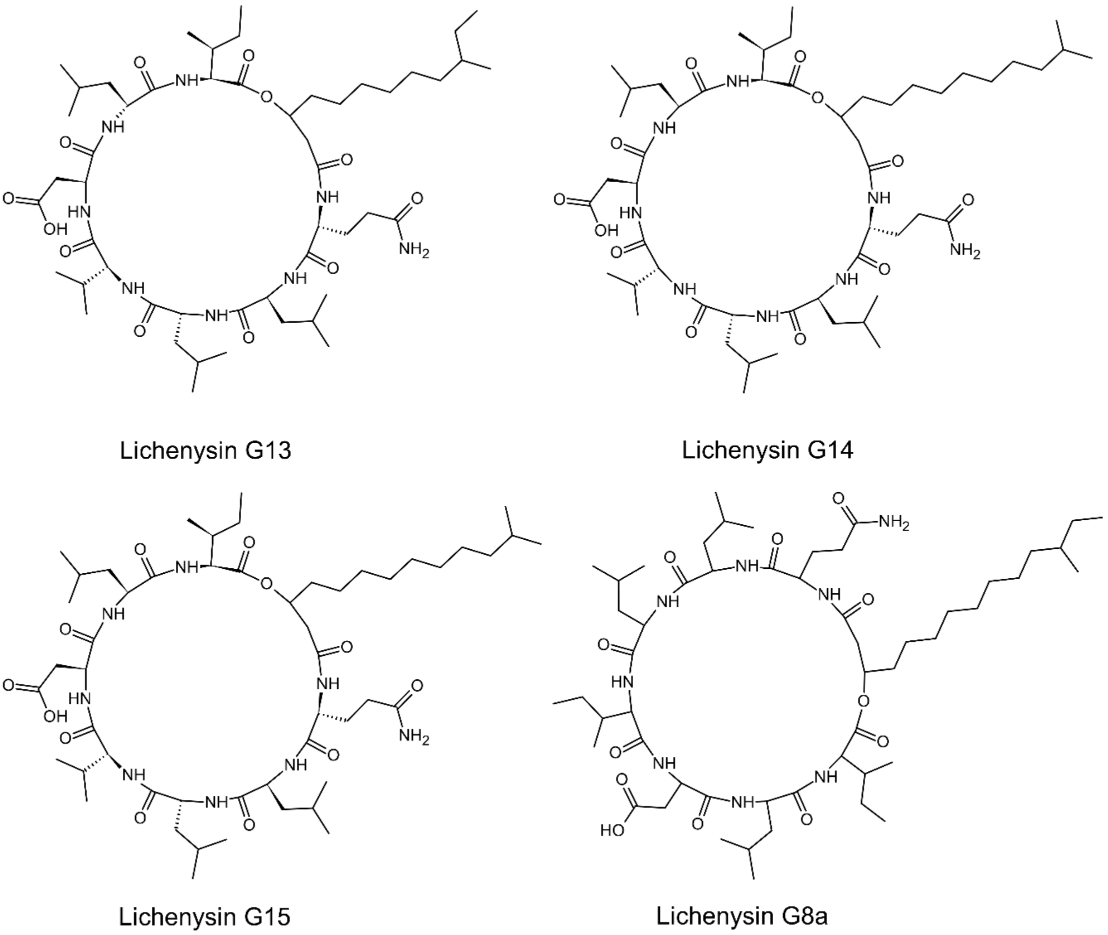

Figure S1. Structures of lichenysins manually dereplicated from the GYM and GYM+Br media extracts of *B. licheniformis* PS108-67a.

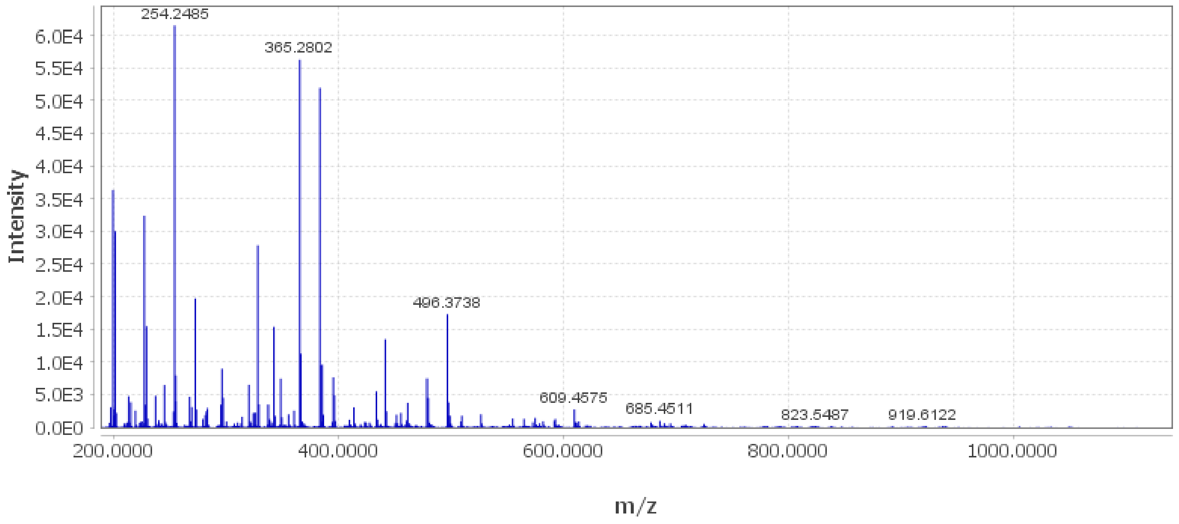

Figure S2. MS/MS spectrum of lichenysin G8a ($C_{54}H_{97}N_8O_{12}$, *m/z* 1049.7234 $[M+H]^+$).

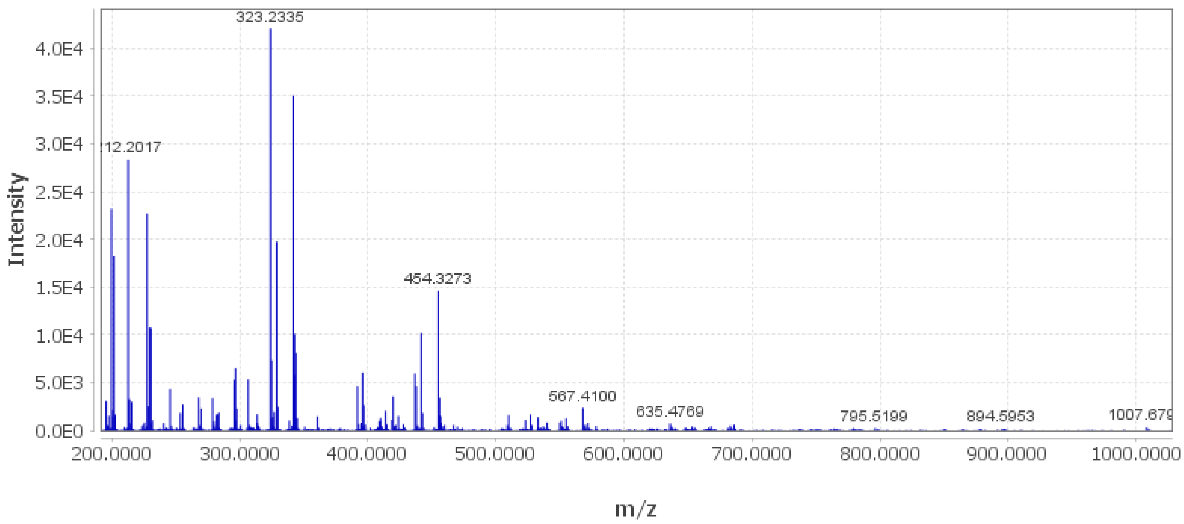

Figure S3. MS/MS spectrum of lichenysin G13 ($C_{51}H_{91}N_8O_{12}$, *m/z* 1007.6754 $[M+H]^+$).

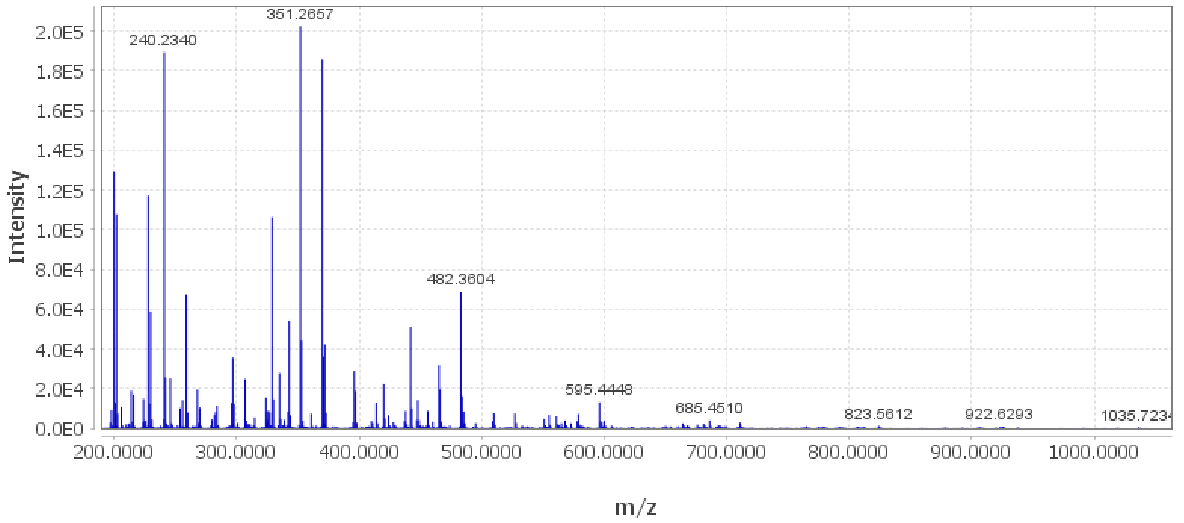

Figure S4. MS/MS spectrum of lichenysin G14 ($C_{52}H_{93}N_8O_{12}$, *m/z* 1021.691 $[M+H]^+$).

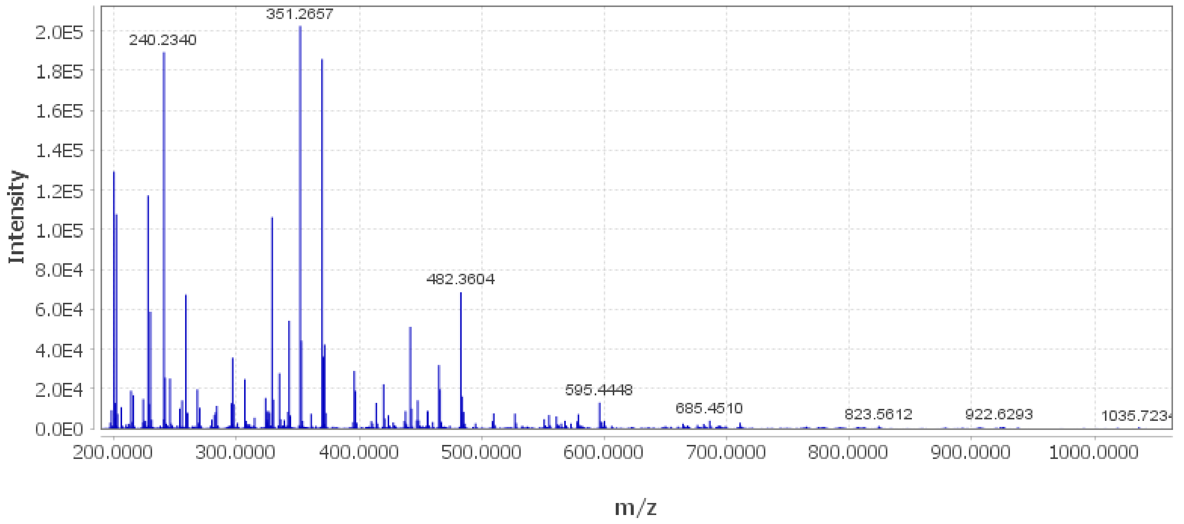

Figure S5. MS/MS spectrum of lichenysin G15 ($C_{53}H_{95}N_8O_{12}$, *m/z* 1035.707 $[M+H]^+$).

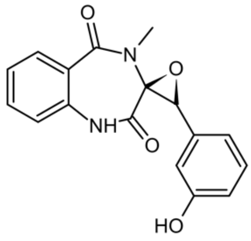

Cyclopenol

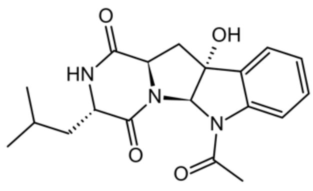

Protubonine A*

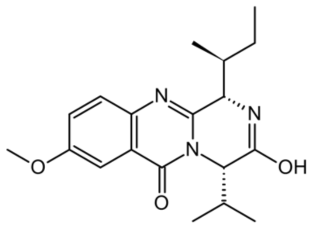

Versicomide A

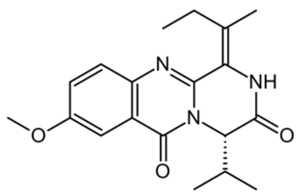

Versicomide B

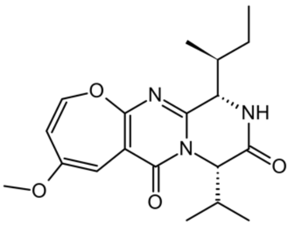

Versicomide D

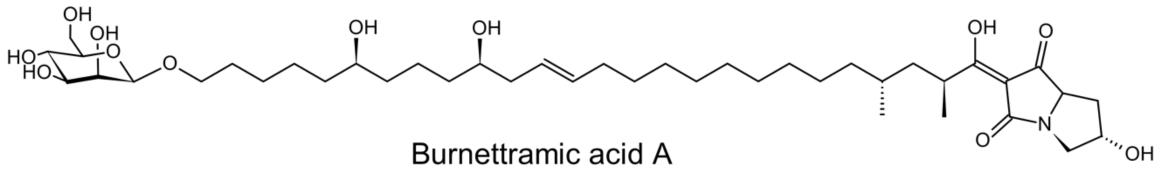

Burnettramic acid A

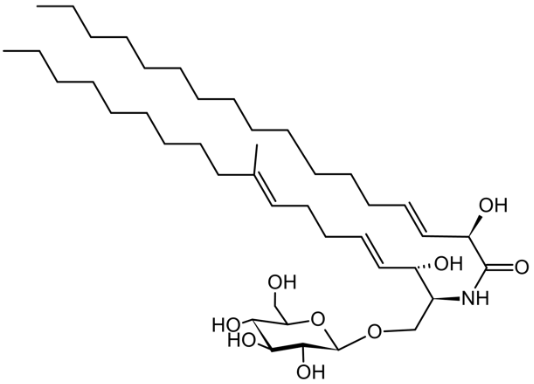

Cerebroside derivative (e.g. Flavuside B)

Figure S6. Structures of the compounds annotated from the GPY+Br, PDA and rice media extracts of the strain *A. versicolor* PS108-62. *Protubonine A was not observed in PDA and Rice media.

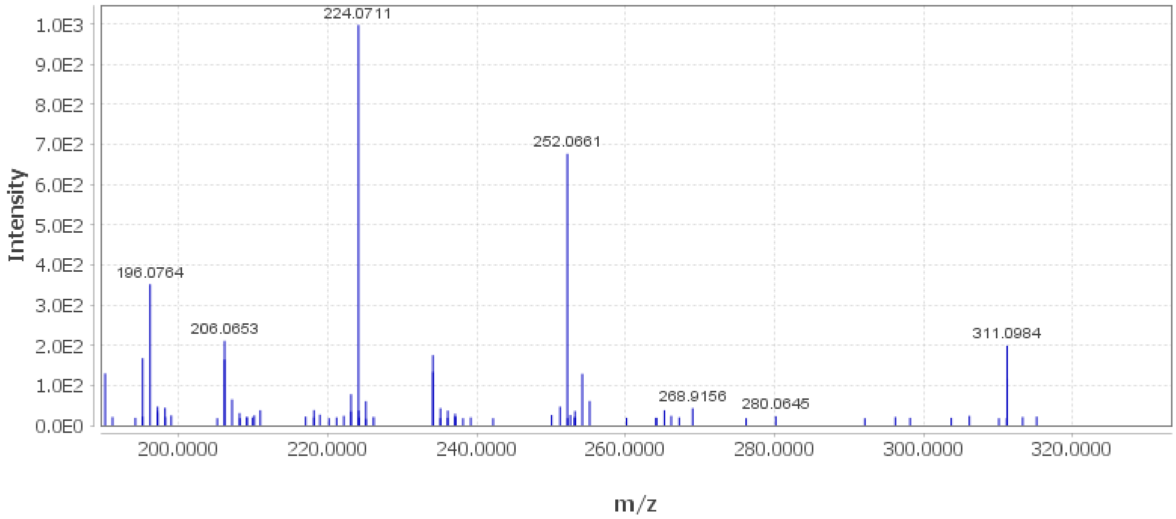

Figure S7. MS/MS spectrum of cyclopenol ($C_{17}H_{15}N_2O_4$, *m/z* 311.1033 $[M+H]^+$).

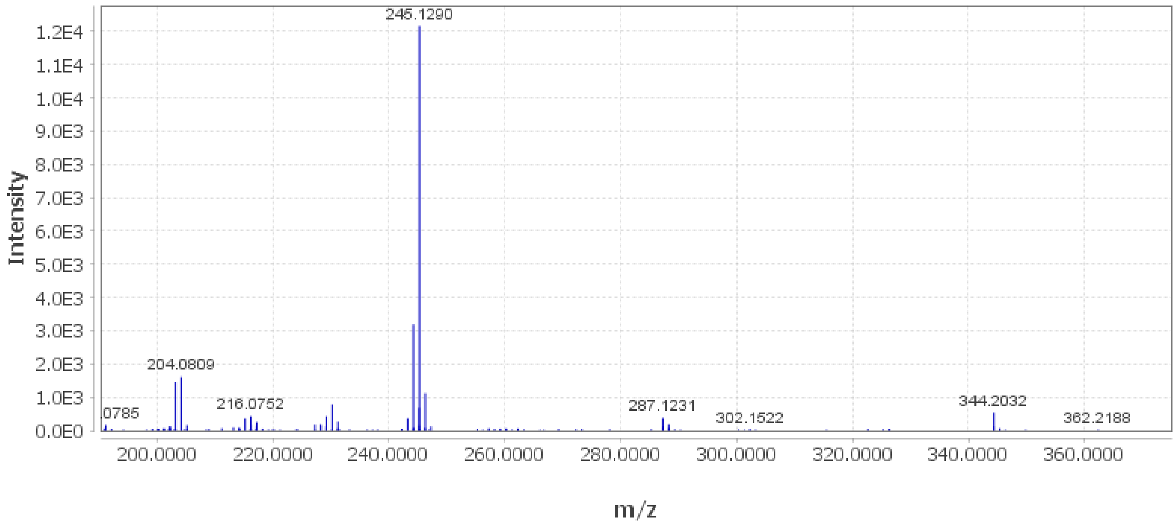

Figure S8. MS/MS spectrum of versicomide A ($C_{19}H_{26}N_3O_3$, *m/z* 344.1975 $[M+H]^+$).

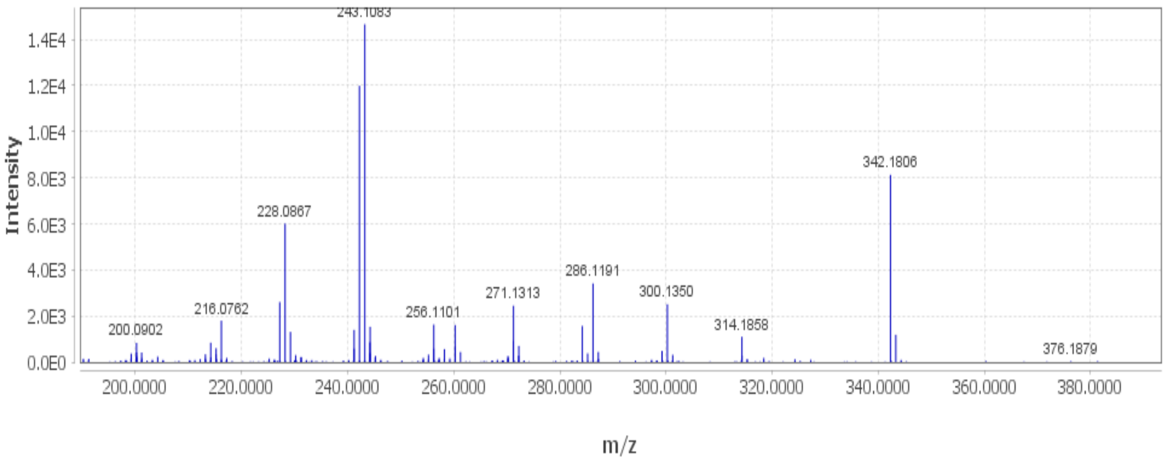

Figure S9. MS/MS spectrum of versicomide B ($C_{19}H_{24}N_3O_3$ *m/z* 342.1818 $[M+H]^+$).

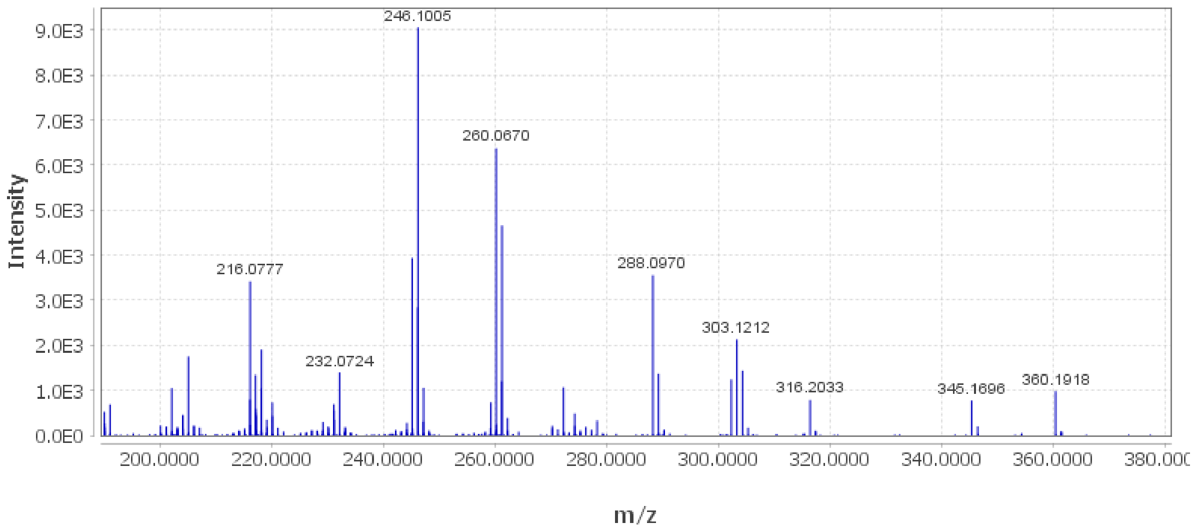

Figure S10. MS/MS spectrum of versicomide D ($C_{19}H_{26}N_3O_4$ *m/z* 360.1923 $[M+H]^+$).

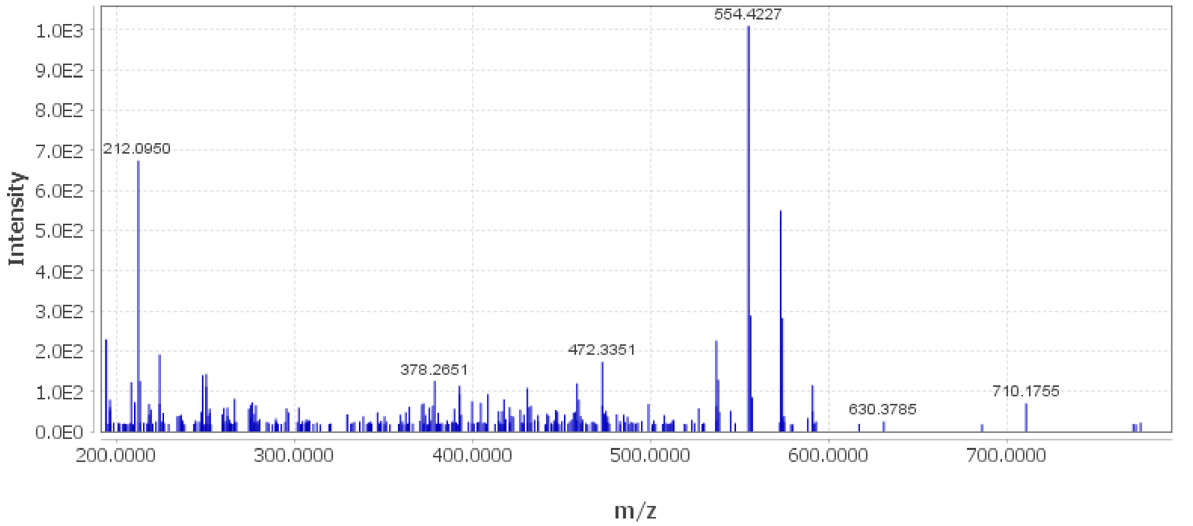

Figure S11. MS/MS spectrum of burnettramic acid A ($C_{41}H_{72}NO_{12}$ *m/z* 770.5042 $[M+H]^+$).

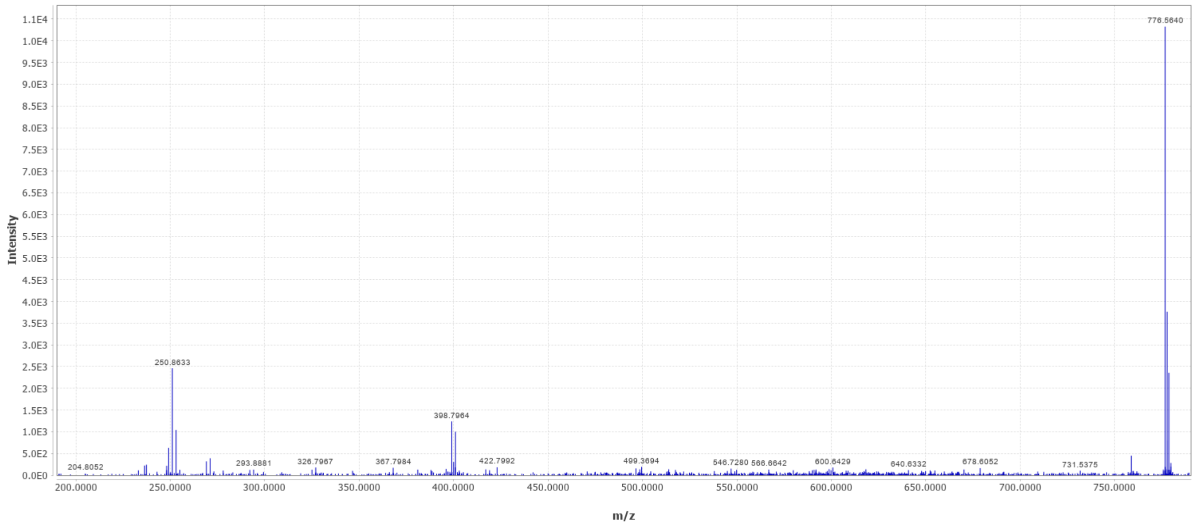

Figure S12. MS/MS spectrum of cerebroside derivative ($C_{43}H_{79}NO_9$ *m/z* 776.5644 $[M+Na]^+$).

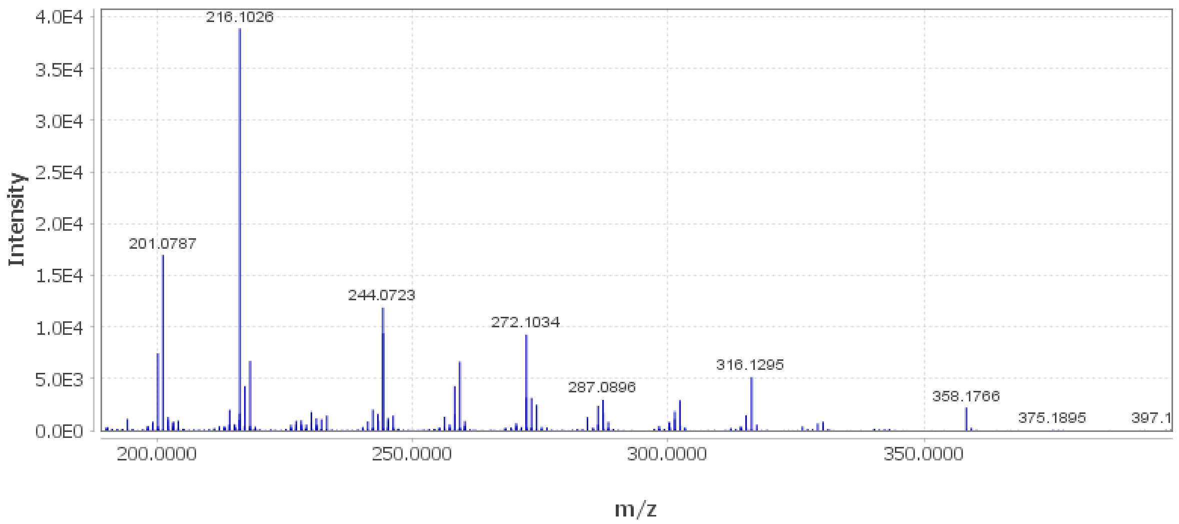

Figure S13. MS/MS spectrum of protubonine A ($C_{19}H_{24}N_3O_4$ *m/z* 358.1768 $[M+H]^+$).

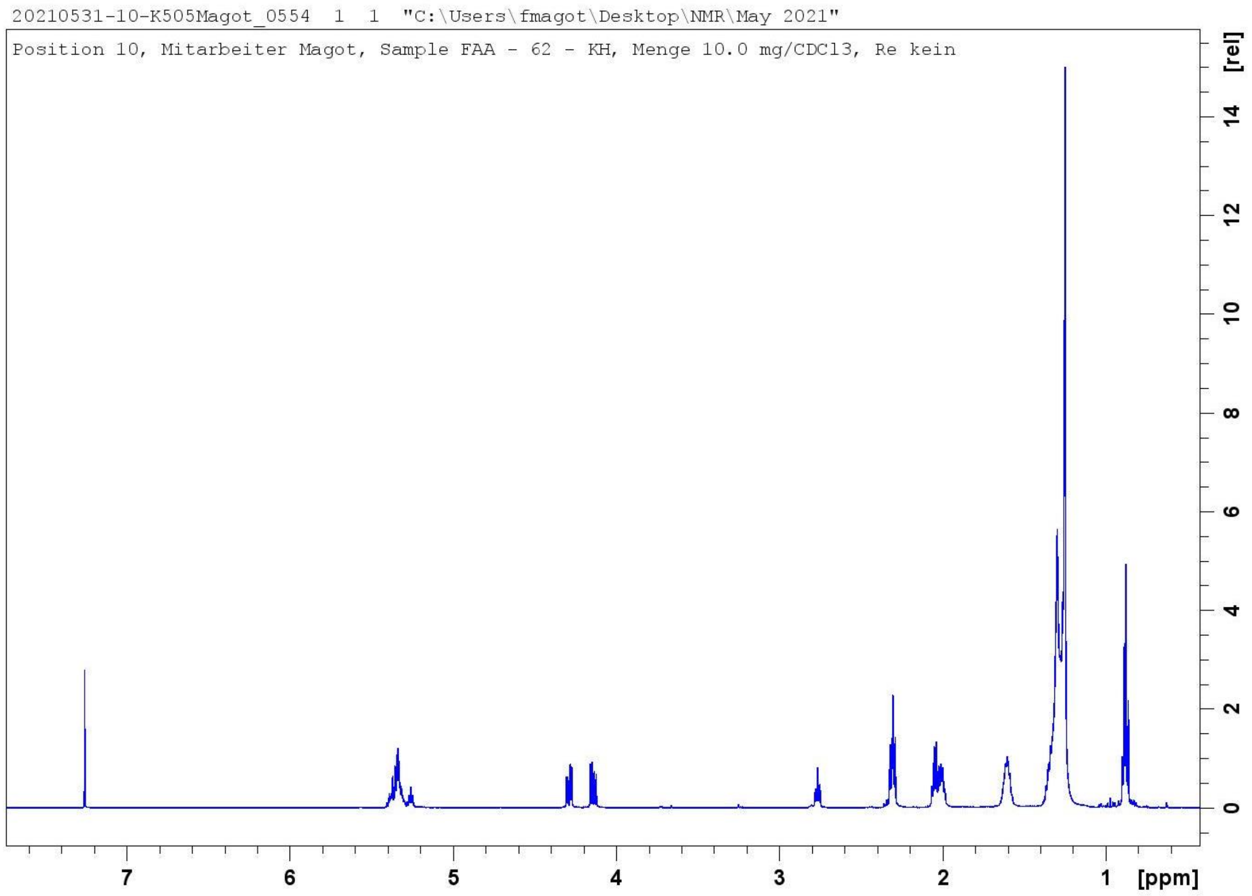

Figure S14. ^{1}H NMR spectrum of the Kh subextract of *A. versicolor* PS108-62 ($CDCl_3$, 500 MHz).

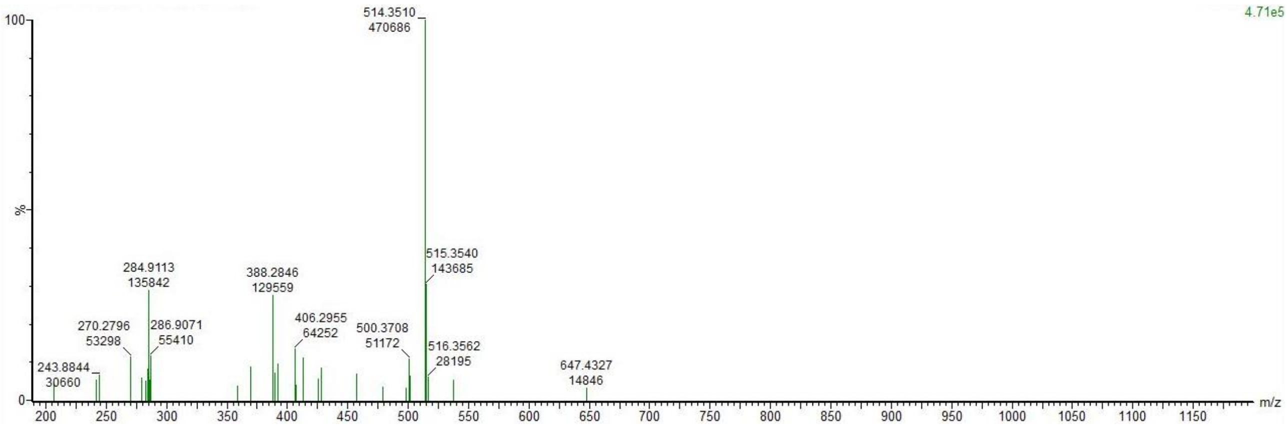

Figure S15. The HR-ESIMS spectrum of versicolide A (**1**).

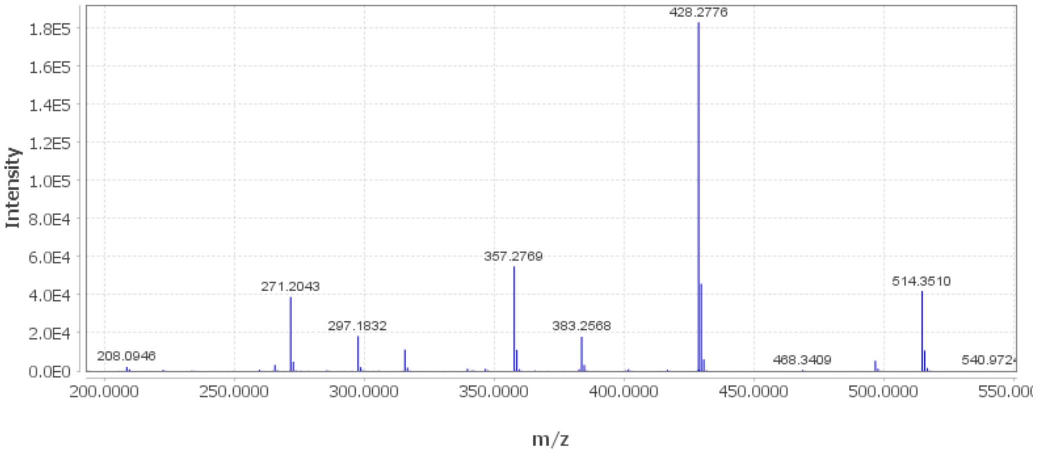

Figure S16. MS/MS spectrum of versicolide A (**1**) ($C_{29}H_{49}NO_5$, *m/z* 514.3510 $[M+Na]^+$).

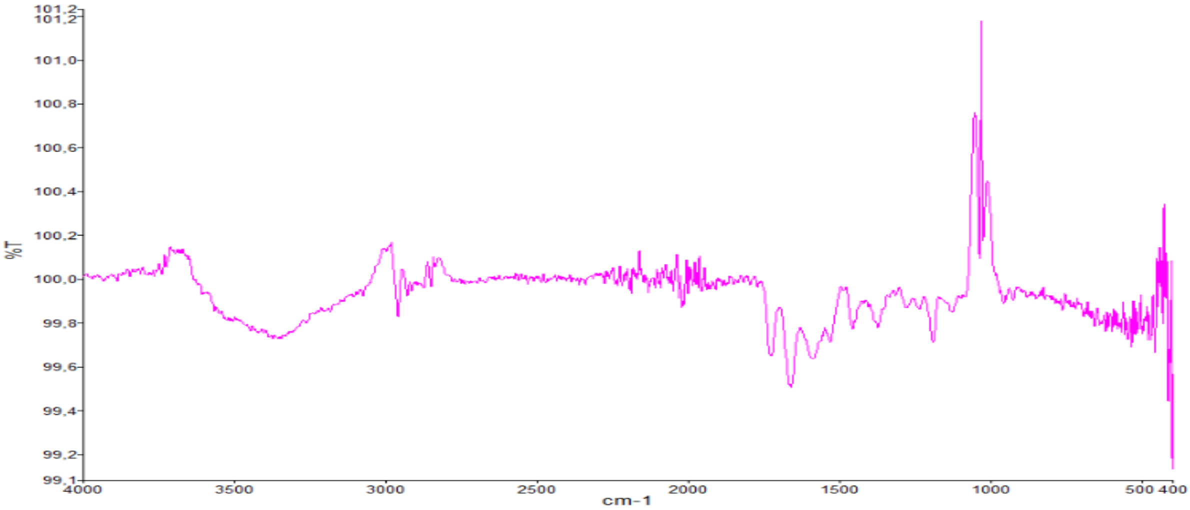

Figure S17. FT-IR spectrum of versicolide A (**1**).

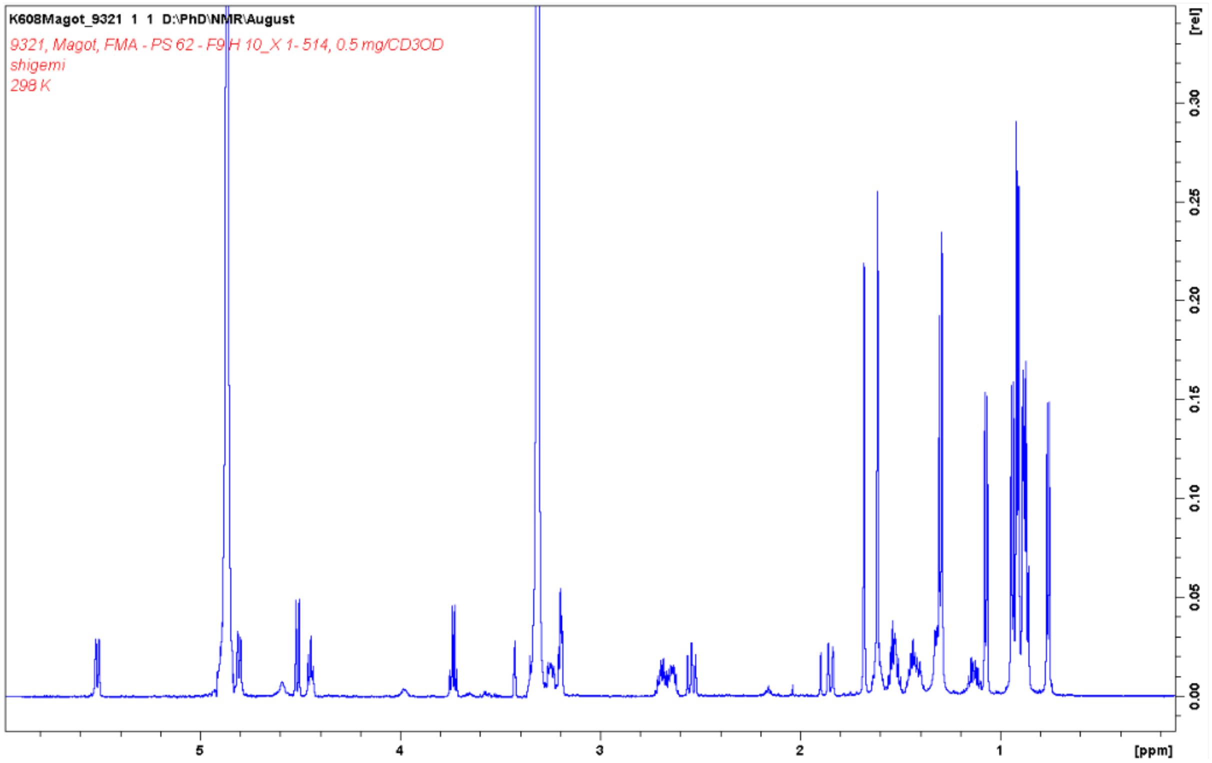

Figure S18. ^{1}H NMR spectrum of versicolide A (**1**) (MeOD, 600 MHz).

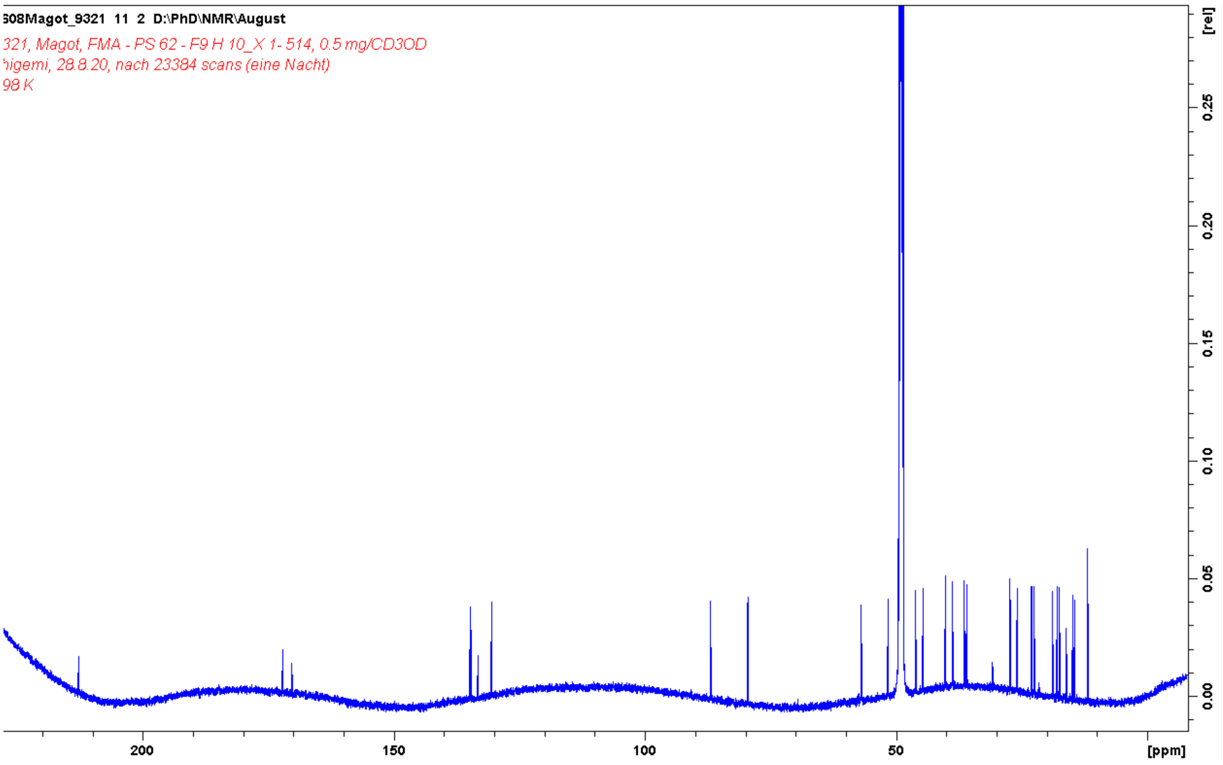

Figure S19. ^{13}C NMR spectrum of versicolide A (**1**) (MeOD, 150 MHz).

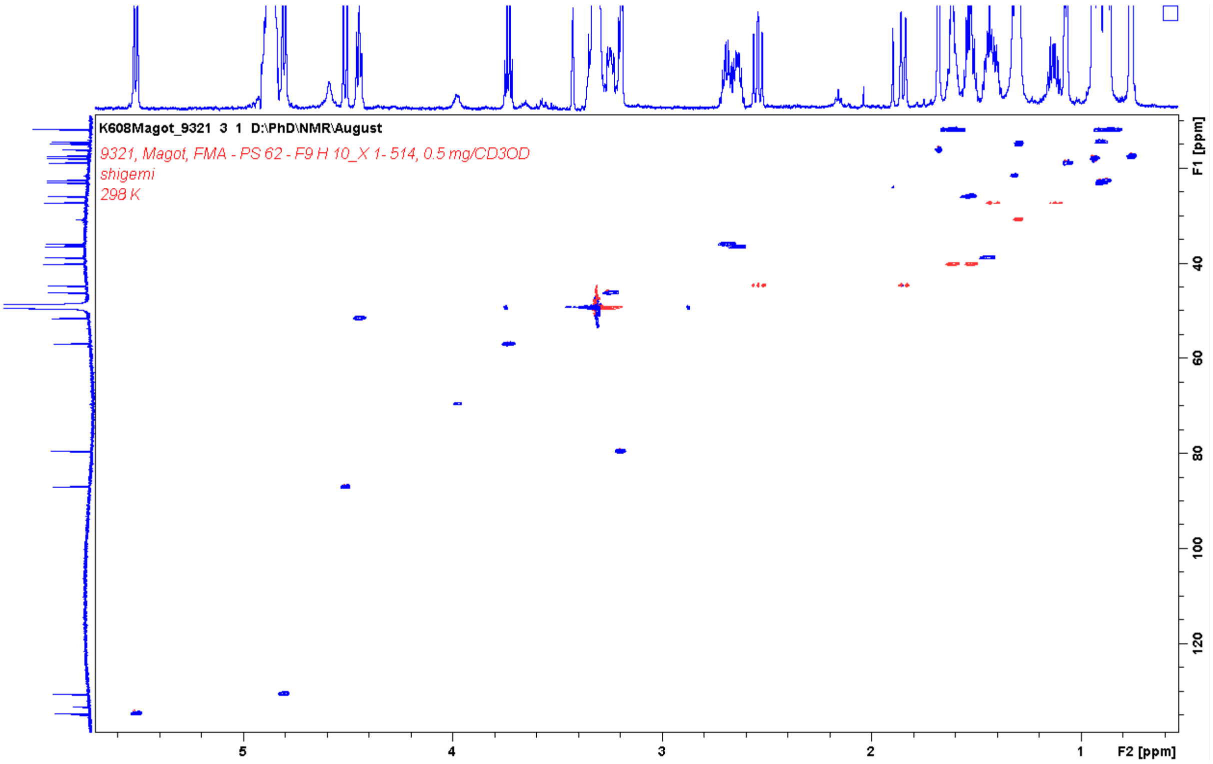

Figure S20. DEPT-HSQC spectrum of versicolide A (**1**) (MeOD, 600/150 MHz).

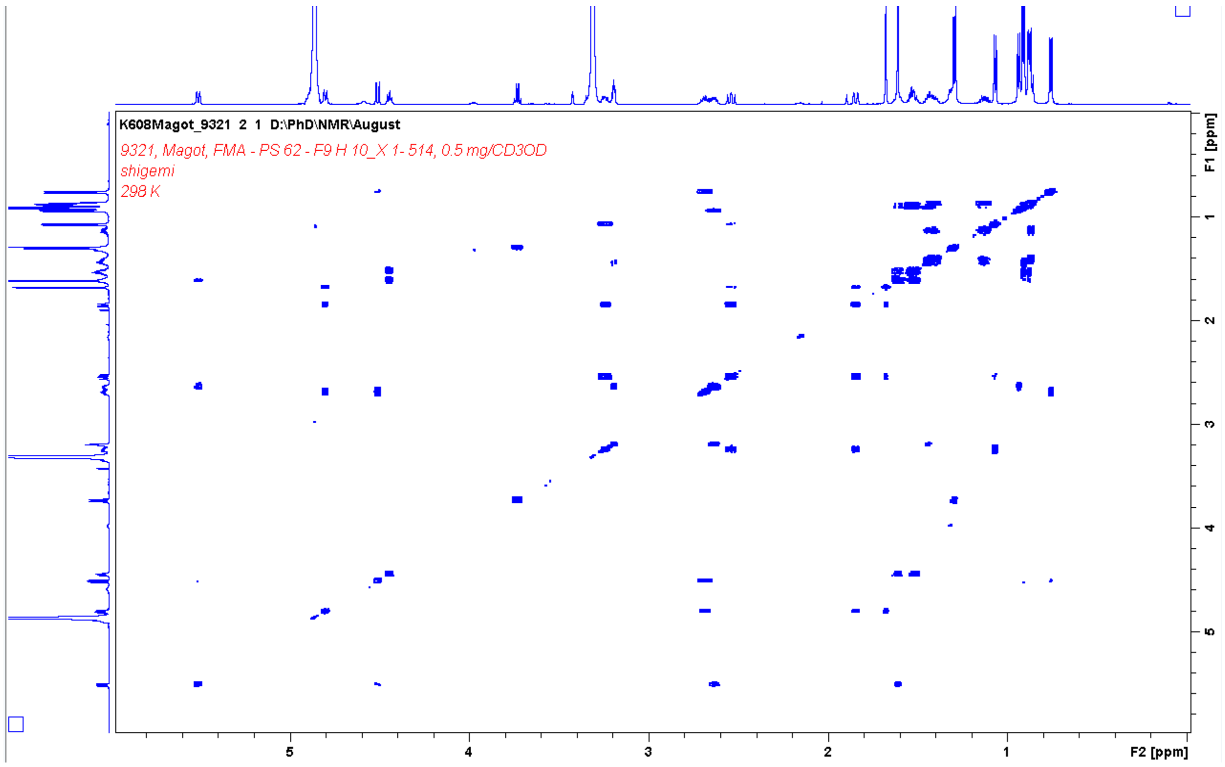

Figure S21. ^{1}H-^{1}H COSY NMR spectrum of versicolide A (**1**) (MeOD, 600 MHz).

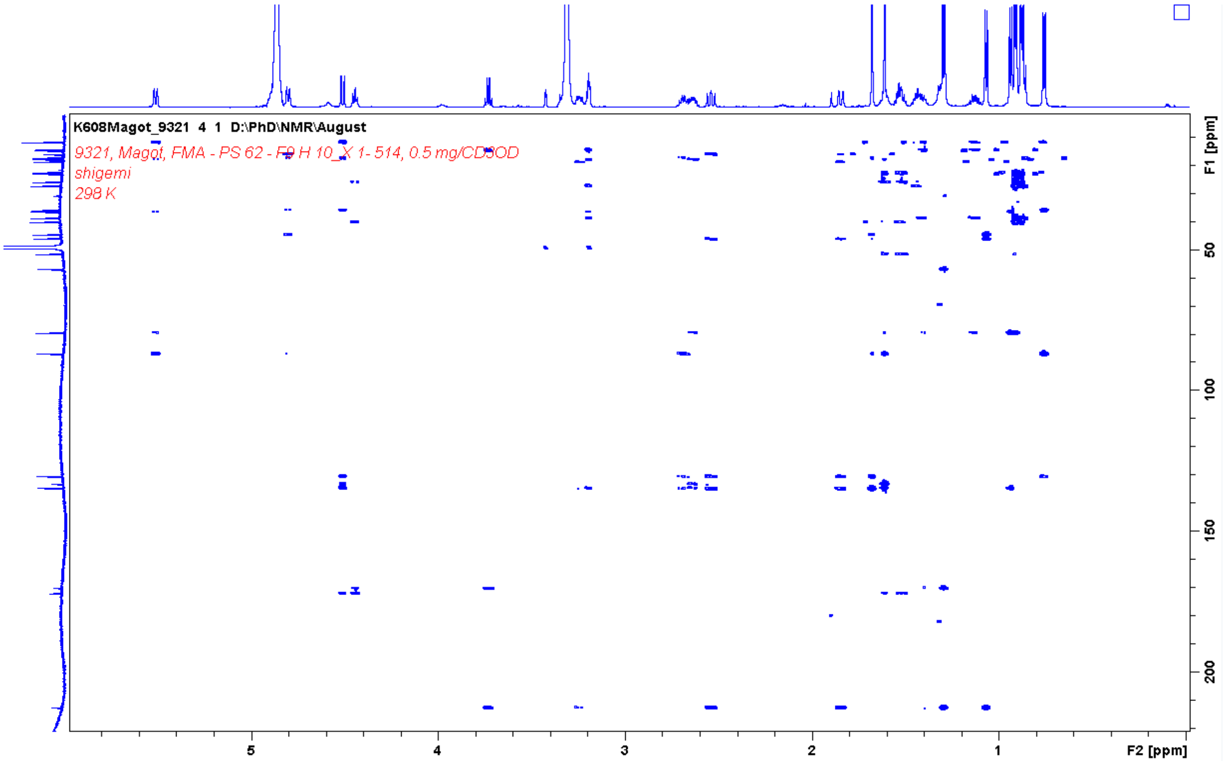

Figure S22. HMBC spectrum of versicolide A (**1**) (MeOD, 600/150 MHz).

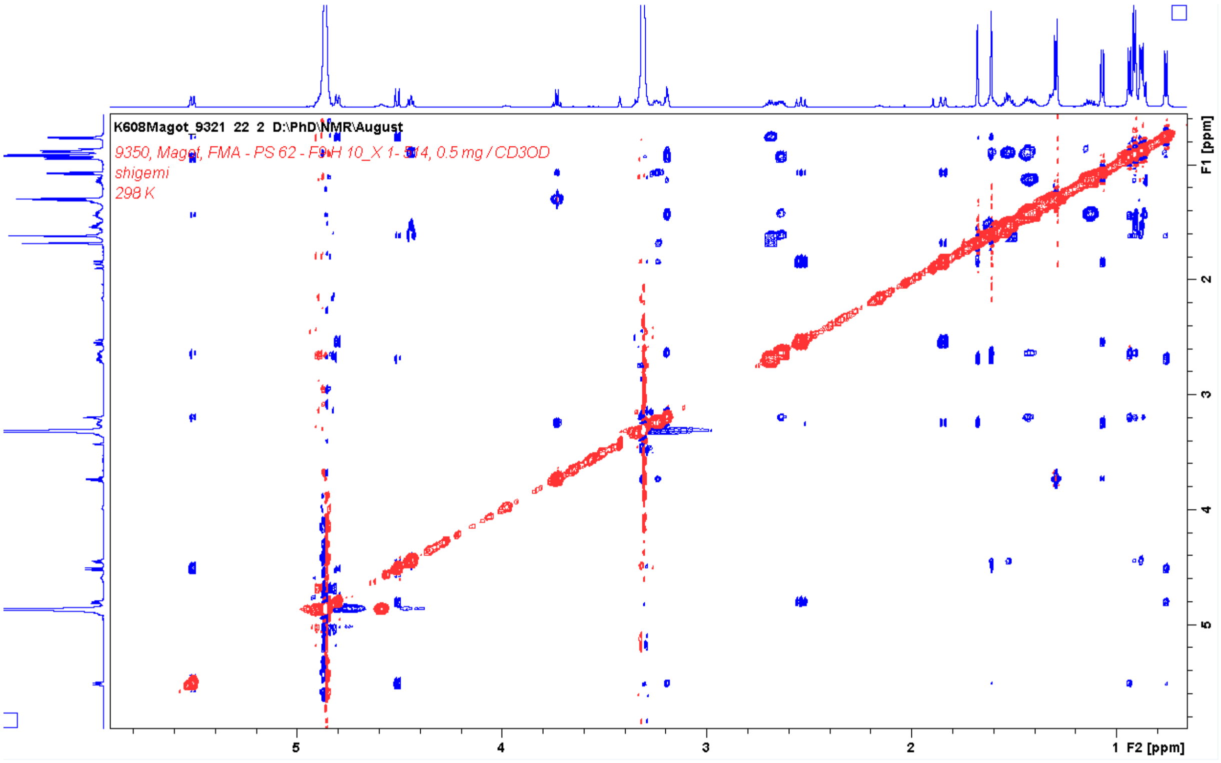

Figure S23. NOESY spectrum of versicolide A (**1**) (MeOD, 600MHz).

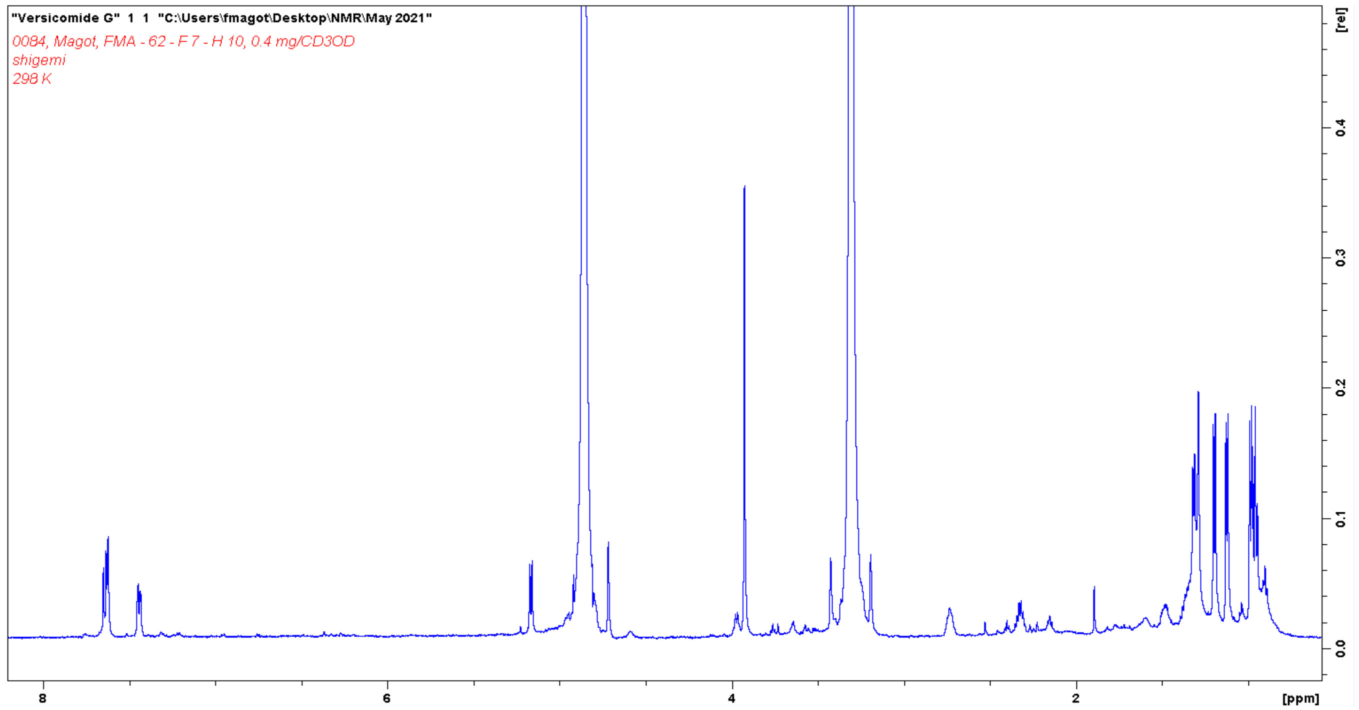

Figure S24. ^{1}H NMR spectrum of (-)-isoversicomide A (**3**) (MeOD, 600 MHz).

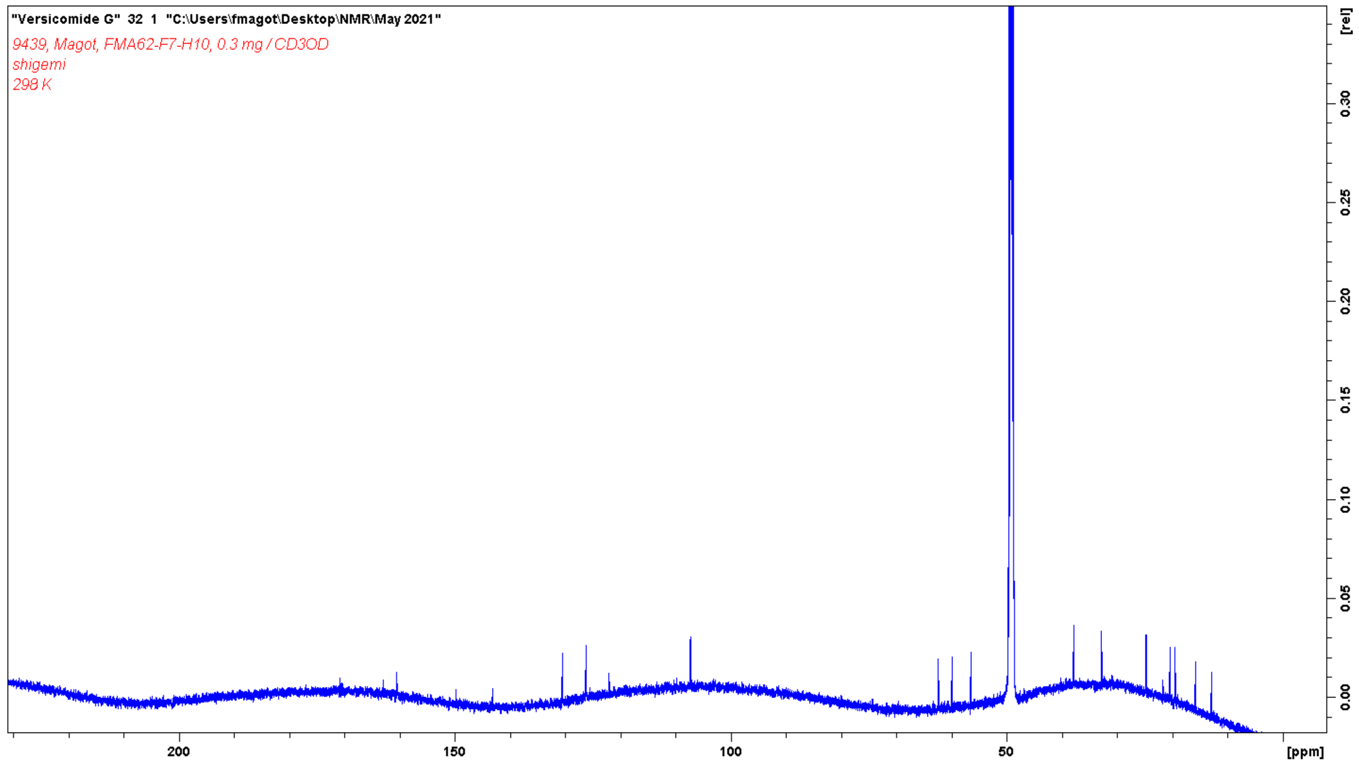

Figure S25. ^{13}C NMR spectrum of (-)-isoversicomide A (**3**) (MeOD, 150 MHz).

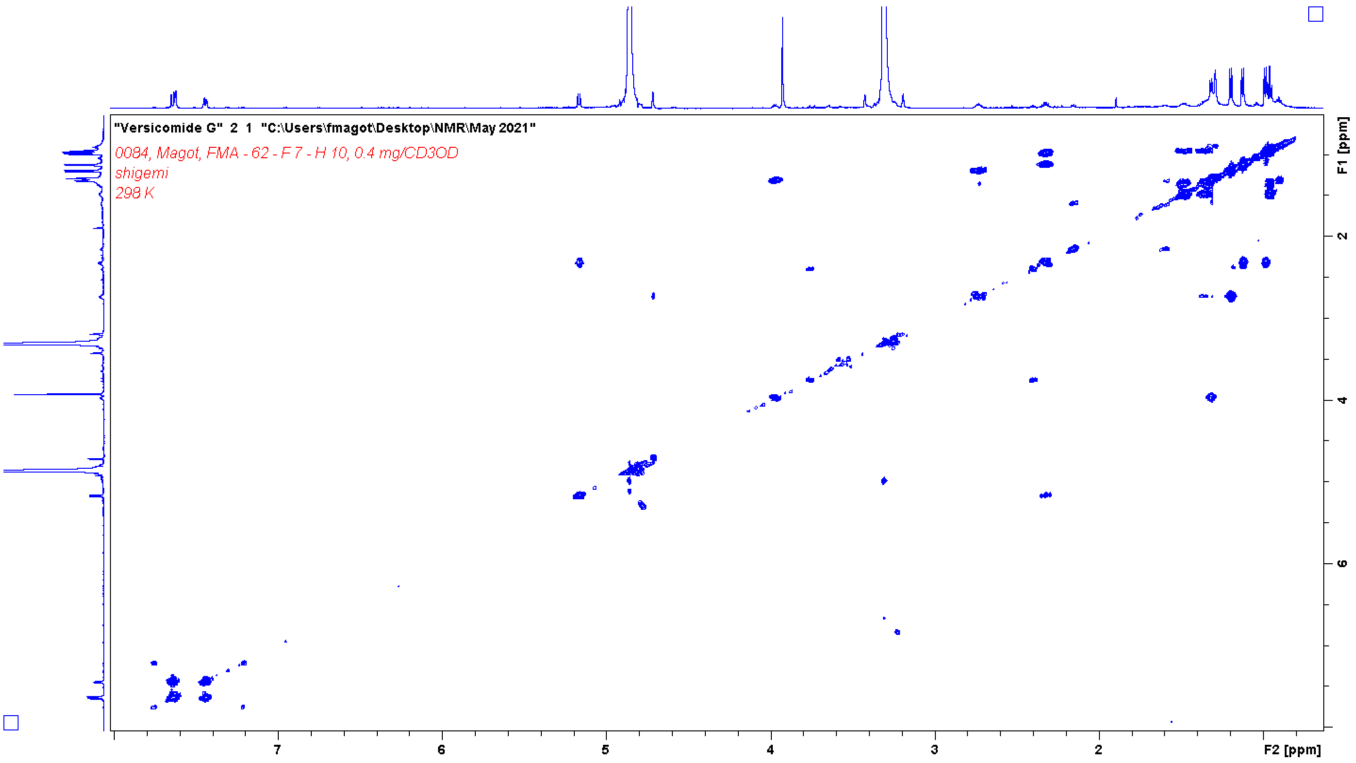

Figure S26. ^1H-^1H COSY NMR spectrum of (-)-isoversicomide A (**3**) (MeOD, 600 MHz).

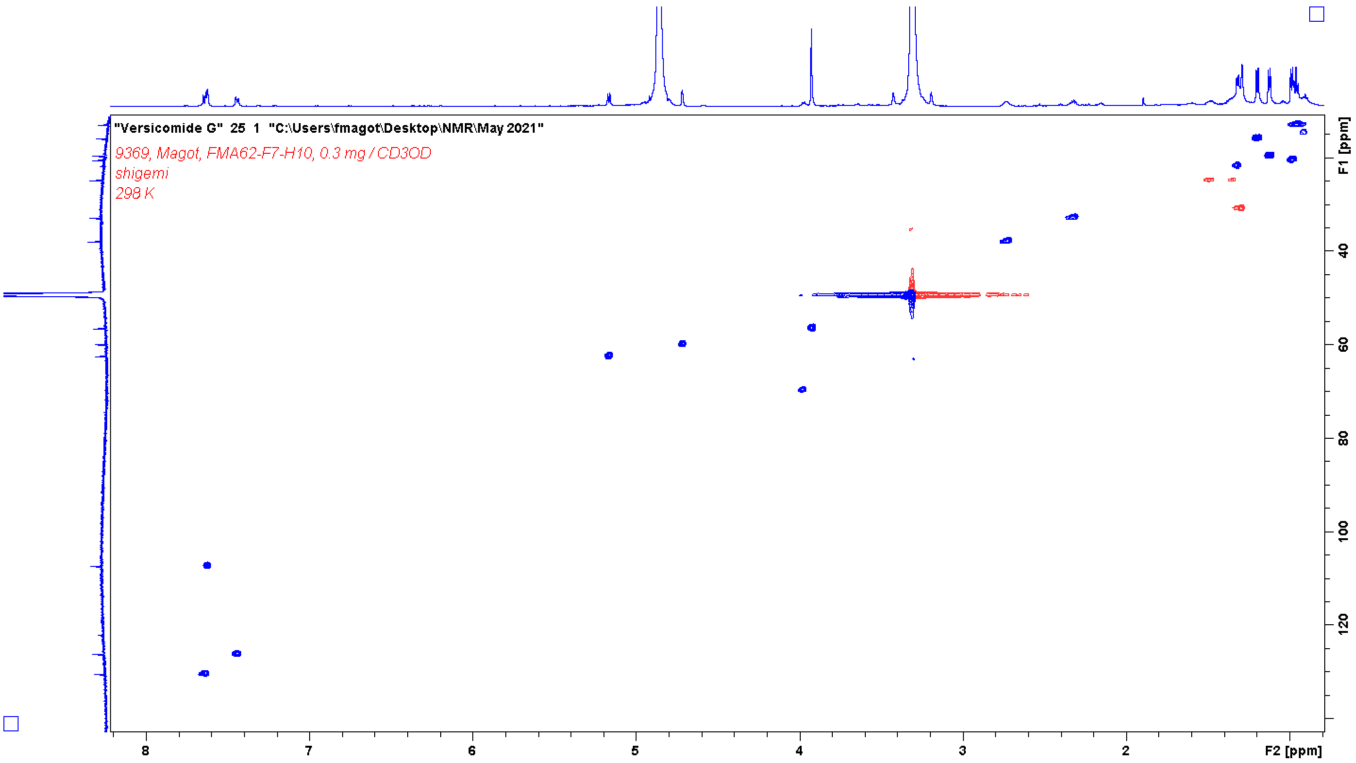

Figure S27. DEPT-HSQC spectrum of (-)-isoversicomide A (**3**) (MeOD, 600/150 MHz).

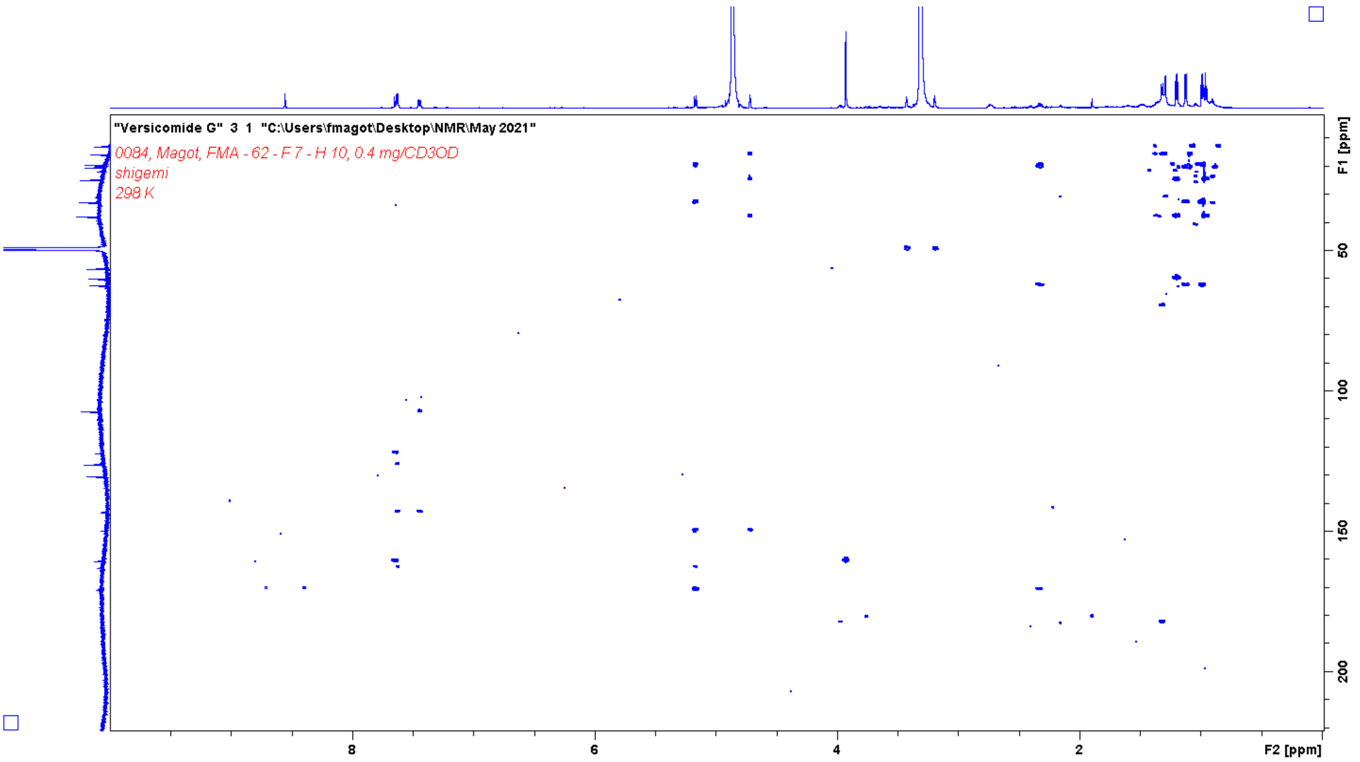

Figure S28. HMBC spectrum of (-)-isoversicomide A (**3**) (MeOD, 600/150 MHz).

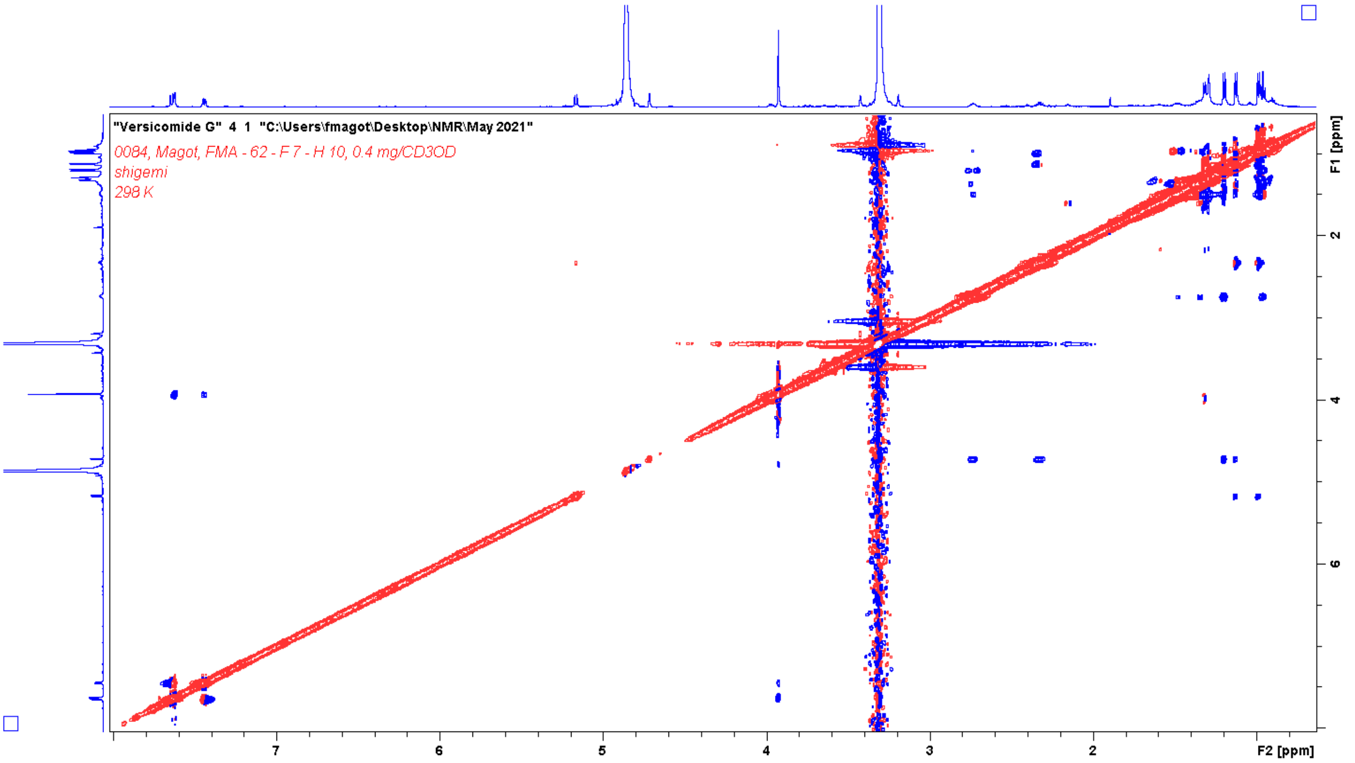

Figure S29. NOESY spectrum of (-)-isoversicomide A (**3**) (MeOD, 600 MHz).

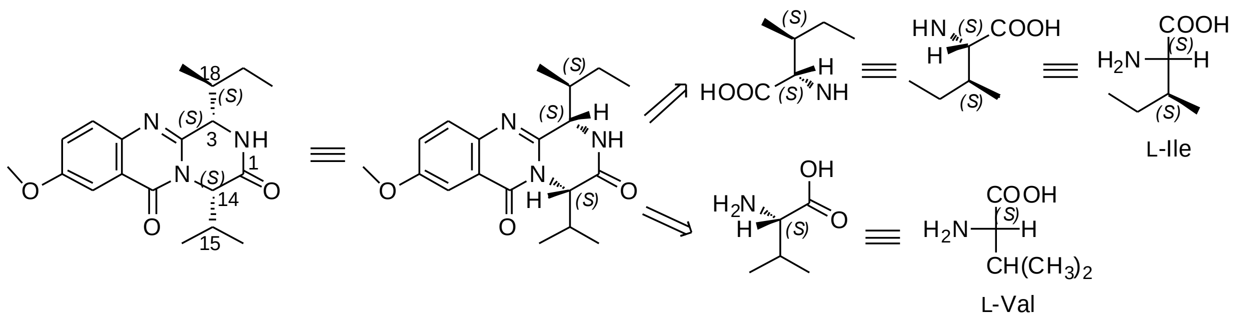

Figure S30. Detailed analysis of (+)-versicomide A drawn by Pan et al. [10] for its amino acid constituents

References

1. Grangemard, I.; Bonmatin, J.-M.; Bernillon, J.; Das, B.C.; Peypoux, F. Lichenysins G, a novel family of lipopeptide biosurfactants from *Bacillus licheniformis* IM 1307: Production, isolation and structural evaluation by NMR and mass spectrometry. *J. Antibiot. (Tokyo).* **1999**, *52*, 363–373.
2. Mohammed, Y.S., Luckner M. The structure of cyclopenin and cyclopenol, metabolic products fron *Penicillium cyclopium* Westling and *Penicilium viridicatum* Westling. *Tetrahedron Lett.* **1963**, *4*, 1953–1958.
3. Lorenzo, P.; Álvarez, R.; De Lera, Á.R. Total synthesis and structural revision of (-)-protubonine A and (-)-protubonine B. *European J. Org. Chem.* **2014**, *2014*, 2557–2564,
4. Lee, S.U.; Asami, Y.; Lee, D.; Jang, J.-H.; Ahn, J.S.; Oh, H. Protuboxepins A and B and protubonines A and B from the marine-derived fungus *Aspergillus* sp. SF-5044. *J. Nat. Prod.* **2011**, *74*, 1284–1287.
5. Pan, C.; Shi, Y.; Chen, X.; Chen, C.-T.A.; Tao, X.; Wu, B. New compounds from a hydrothermal vent crab-associated fungus *Aspergillus versicolor* XZ-4. *Org. Biomol. Chem.* **2017**, *15*, 1155–1163.
6. Li, J.; Chen, M.; Hao, X.; Li, S.; Li, F.; Yu, L.; Xiao, C.; Gan, M. Structural revision and absolute configuration of burnettramic acid A. *Org. Lett.* **2019**, *22*, 98–101.
7. Li, H.; Gilchrist, C.L.M.; Lacey, H.J.; Crombie, A.; Vuong, D.; Pitt, J.I.; Lacey, E.; Chooi, Y.-H.; Piggott, A.M. Discovery and heterologous biosynthesis of the burnettramic acids: rare PKS-NRPS-derived bolaamphiphilic pyrrolizidinediones from an Australian fungus, *Aspergillus burnettii*. *Org. Lett.* **2019**, *21*, 1287–1291.
8. Jiang, T.; Li, T.; Li, J.; Fu, H.-Z.; Pei, Y.-H.; Lin, W.-H. Cerebroside analogues from marine-derived fungus *Aspergillus flavipes*. *J. Asian Nat. Prod. Res.* **2004**, *6*, 249–257.
9. Yang, G.; Sandjo, L.; Yun, K.; Leutou, A.S.; Kim, G.-D.; Choi, H.D.; Kang, J.S.; Hong, J.; Son, B.W. Flavusides A and B, antibacterial cerebrosides from the marine-derived fungus *Aspergillus flavus*. *Chem. Pharm. Bull.* **2011**, *59*, 1174–1177.
10. Pan, C.; Shi, Y.; Chen, X.; Chen, C.-T.A.; Tao, X.; Wu, B. New compounds from a hydrothermal vent crab-associated fungus *Aspergillus versicolor* XZ-4. *Org. Biomol. Chem.* **2017**, *15*, 1155–1163.
